# Gut Microbiota and White Matter Integrity: A Two-Sample Mendelian Randomization Analysis

**DOI:** 10.1523/ENEURO.0586-24.2025

**Published:** 2025-08-29

**Authors:** Lan Zhang, Xiao-Wei Pang, Lu-Yang Zhang, Li-Fang Zhu, Wan-Ning Li, Yun-Hui Chu, Luo-Qi Zhou, Dai-Shi Tian, Chuan Qin, Lian Chen

**Affiliations:** ^1^Department of Neurology, Tongji Hospital, Tongji Medical College and State Key Laboratory for Diagnosis and Treatment of Severe Zoonotic infectious Diseases, Huazhong University of Science and Technology, Wuhan 430030, China; ^2^Hubei Key Laboratory of Neural Injury and Functional Reconstruction, Huazhong University of Science and Technology, Wuhan 430030, China; ^3^Key Laboratory of Vascular Aging, Ministry of Education, Tongji Hospital of Tongji Medical College, Huazhong University of Science and Technology, Wuhan 430030, People’s Republic of China

**Keywords:** gut microbiota; Mendelian randomization, white matter connectivity, white matter hyperintensities, white matter microstructure

## Abstract

The causal relationship between gut microbiota (GM) and white matter injury and communication remains unclear. We aimed to scrutinize the plausible causal impact of GM on white matter hyperintensities (WMHs), white matter microstructure, white matter connectivity, and multiple neurological diseases via Mendelian randomization study. We identified four WMH-related bacterial taxa, including class *Melainabacteria*, order *Gastranaerophilales*, family *Alcaligenaceae*, and genus *Ruminiclostridium 6*. In addition, three bacterial taxa were discovered that have consistent effect on multiple aspects of white matter microstructure. Furthermore, we found 12 strong associations between genetic liability in GM and white matter connectivity. Among these bacterial taxa, the family *Clostridiaceae 1* demonstrated a protective effect against ischemic stroke (IS). The genus *Barnesiella* showed protective effect on IS and small vessel stroke while posed a risk effect on neuromyelitis optica spectrum disorder (NMOSD), as well as on aquaporin-4 immunoglobulin G-positive neuromyelitis optica spectrum disorder (AQP4-IgG+ NMOSD). The order *Desulfovibrionales* and family *Desulfovibrionaceae* showed protective effect against cardioembolic stroke, and the genus *Ruminococcus gnavus group* showed a protective effect on amyotrophic lateral sclerosis. In terms of the mapped genes of statistically significant bacterial taxa, genes such as *CPNE1*, *PIGU*, *MED22*, *SURF6*, *DOCK10*, and *COPS3* exhibited a significant causal correlation with the corresponding white matter connectivity. This study demonstrated a genetically predicted causal relationship between GM and WMH, white matter microstructure, white matter connectivity, and multiple neurological diseases, based on GWAS data from mixed-sex cohorts without sex-stratified summary statistics. These findings highlight the potential role of GM in influencing brain structural integrity.

## Significance Statement

This is the first Mendelian randomization (MR) study to establish a relationship between the gut microbiota (GM) and brain white matter, identifying specific bacterial taxa with genetic responsibility for causal relationships with brain white matter. Our results indicate that 17 bacterial taxa associated with several white matter integrity and communication-related indexes. The MR study also identifies potential effects of GM in multiple neurological diseases, especially family *Clostridiaceae 1*, order *Desulfovibrionales*, and *family Desulfovibrionaceae*. This is the first study to utilize MR analysis in combination with functional mapping and annotation analysis to explore the causal relationship between GM and brain white matter. Genes such as *CPNE1*, *PIGU*, *MED22*, *SURF6*,* DOCK10*, and *COPS3* exhibit a significant causal correlation with white matter connectivity.

## Introduction

The gut microbiota (GM), a complex community of microorganisms residing in the human gastrointestinal tract, plays a crucial role in human health (Grenham et al., 2011). Recent studies have acknowledged the bidirectional communication between the brain and the gastrointestinal system (Gawey et al., 2025). The gut–brain axis has proved to be a crucial pathway by which GM exert substantial influence on host brain function principally operating via three primary pathways: immune modulation, neuroendocrine signaling, and vagus nerve communication (Collins et al., 2012; Morais et al., 2021).

White matter, consisting mainly of axons, oligodendrocytes, microglia, and astrocytes, is fundamental for information processing and communication within the central nervous system (Xu et al., 2023). The processes of axonal degeneration, demyelination, and neuroinflammation are often identified as primary contributors to white matter injury (Wang et al., 2015; Dombrowski et al., 2017). Growing evidence indicates that GM and its metabolites significantly influence oligodendrocyte generation and myelination processes, potentially contributing to the onset of neurological disorders such as multiple sclerosis (MS), ischemic stroke (IS), Alzheimer's disease (AD), and Parkinson's disease (PD; Cryan et al., 2020; Dong et al., 2022). Advancements in diffusion magnetic resonance imaging (dMRI), including techniques like diffusion tensor imaging (DTI), neurite orientation dispersion and density imaging (NODDI), and tractography, have enabled noninvasive in vivo quantification of white matter microstructure and connectivity, providing critical insights into brain function and underlying pathophysiological mechanisms. However, the connection between GM and white matter remains poorly understood (Catani et al., 2012; Aung et al., 2013).

Mendelian randomization (MR) has become a key tool in epidemiology for drawing causal inferences. It leverages genetic variations, which are randomly distributed at conception, as instrumental variables (IVs) to estimate the causal effect of exposures on outcomes. By minimizing the effects of reverse causation and confounding factors, MR provides more robust and reliable estimates of causality compared with conventional observational studies (Sekula et al., 2016; Emdin et al., 2017).

This study employs MR to explore the potential causal relationships between GM and white matter hyperintensities (WMHs), white matter microstructure, white matter connectivity, and multiple neurological diseases. By leveraging genetic variants as IVs, the influence of specific bacterial taxa on white matter was inferred, offering new insights into how GM might affect brain health and contribute to the pathophysiology of neurological disorders. Besides, the functional mapping and annotation (FUMA) method is a commonly used computational tool that can map genetic variants to genes based on their genomic locations. This approach enables the identification of key genetic variants and the exploration of their potential biological mechanism (Watanabe et al., 2017). In terms of the mapped genes of statistically significant bacterial taxa, genes such as* CPNE1*, *PIGU*, *MED22*, *SURF6*, *DOCK10*, and *COPS3*, exhibited a significant causal correlation with the corresponding white matter connectivity.

## Materials and Methods

### Study designs

**Figure 1.**
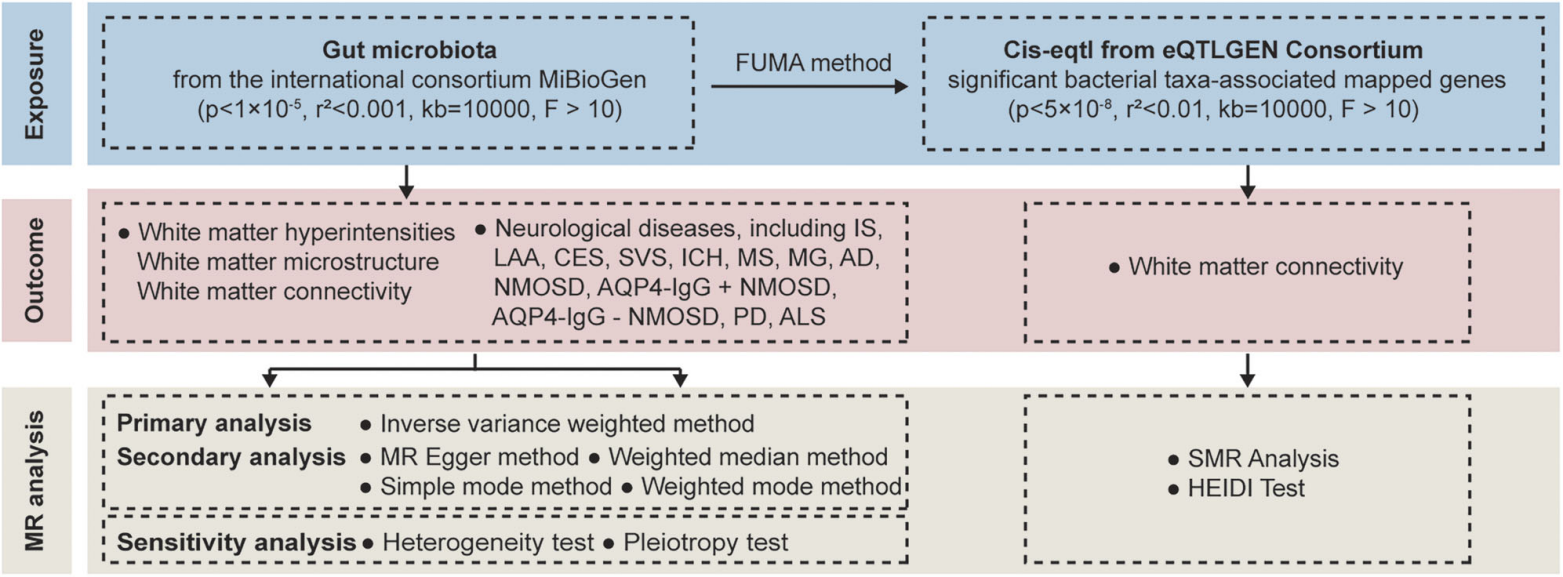
Flow chart. FUMA, functional mapping and annotation; SMR, summary Mendelian randomization.

The flowchart of this study was shown in Figure 1. MR analysis was used to investigate the causal association between GM and WMH, white matter microstructure, white matter connectivity, and multiple neurological diseases. Reliable results were based on the following three assumptions of MR analysis: (1) the strong relationship between the IVs and exposure; (2) IVs should be independent, ensuring no relation with confounding factors; and (3) IVs influence outcome through exposure rather than other factors.

### Data source

Genome-wide association study (GWAS) data for GM phenotypes (*N* = 18,340) were obtained from the international consortium MiBioGen (Kurilshikov et al., 2021). The data encompassed 211 bacterial taxa, including 119 genera, 32 families, 20 orders, 16 classes, and 9 phyla, and 15 unidentified special traits were excluded. GWAS of WMH was collected from brain MRI genotype data of 50,970 participants (*N* = 48,454 Europeans and 2,516 African-Americans), originating from the Cohorts for Heart and Aging Research in Genomic Epidemiology (CHARGE) and the UK Biobank (Sargurupremraj et al., 2020). GWAS summary statistics of white matter microstructure were obtained from a genetic study on IDPs of the brain of 31,356 individuals of European ancestry from UK Biobank (Smith et al., 2021) and GWAS summary statistics of white matter connectivity from 26,333 UK Biobank participants with structural and diffusion MRI scans (Wainberg et al., 2024). Summary statistics data of 13 neurological diseases were obtained from public labeled references (Table 1). The GWAS summary statistics were derived from mixed-sex cohorts, and sex-stratified data were not provided by the original studies.

**Table 1.** Summary table of disease data sources

Phenotype	Sample size	*N* case/*N* control	Population	PMID/website
IS	440,328	34,217/406,111	European	29531354
LAA	150,765	4,373/406,111	European	29531354
CES	211,763	7,193/406,111	European	29531354
SVS	198,048	5,386/192,662	European	29531354
ICH	375,773	4,056/371,717	European	https://www.finngen.fi/en
MS	115,803	47,429/68,374	European	31604244
MNOSD	546	86/460	European	29769526
AQP4-IgG+ NMOSD	526	66/460	European	29769526
AQP4-IgG− NMOSD	480	20/460	European	29769526
MG	38,243	1,873/36,370	European	35074870
AD	487,511	85,934/401,577	European	35379992
PD	482,730	33,674/449,056	European	31701892
ALS	80,610	20,806/59,804	European	29566793

IS, ischemic stroke; LAA, large artery atherosclerosis; CES, cardioembolism; SVS, small vessel stroke; ICH, intracerebral hemorrhage; MS, multiple sclerosis; MG, myasthenia Gravis; AD, Alzheimer’s disease; NMOSD, neuromyelitis optica spectrum disorder; AQP4-IgG+ NMOSD, aquaporin-4 immunoglobulin G-positive neuromyelitis optica spectrum disorder; AQP4-IgG− NMOSD, aquaporin-4 immunoglobulin G-negative neuromyelitis optica spectrum disorder; PD, Parkinson’s disease; ALS, amyotrophic lateral sclerosis.

### Instrumental variable selection

To ensure the reliability and accuracy of the study results, single nucleotide polymorphisms (SNPs) that strongly associated with exposure were carefully selected based on the threshold of *p* value (*p* < 1 × 10^−5^; Sanna et al., 2019), which ensures that SNPs are filtered to an appropriate quantity. To avoid bias from independent IVs, SNPs with linkage disequilibrium were clumped using a threshold of *R*^2^ < 0.001 within a 10,000 kb window. The *F* statistic for each SNP (*F* = beta^2^/se^2^) was used to evaluate, and strong instrument variables with *F* > 10 remained (Burgess et al., 2011; Harroud et al., 2021).

### Statistical analysis

The inverse-variance weighted (IVW) method was primarily utilized to assess the potential causal relationship between GM and WMH, white matter microstructure, white matter connectivity, multiple neurological diseases. The Wald ratio was used when there was one SNP. In addition, the weighted median, MR-Egger regression, weighted mode, and simple mode method were also performed as secondary methods. To evaluate heterogeneity, we calculated Cochran's Q statistic. If significant heterogeneity (*p* < 0.05) was observed, the random-effects model was automatically applied by the MR method; otherwise, the fixed-effects model was used, ensuring the accuracy and robustness of the results. Pleiotropy was assessed using the MR-Egger method and MR-PRESSO. Bonferroni correction was implemented for multiple testing and screening for statistically significant bacterial taxa and WMH, white matter microstructure, and white matter connectivity with *p* value less than 2.55 × 10^−4^ (0.05/196), while special bacterial taxa and multiple neurological diseases with *p* value less than 2.94 × 10^−3^ (0.05/17). Association with *p* value less than 0.05 but greater than this corrected threshold was regarded as nominally significant. The analyses were primarily carried out using the “TwoSampleMR” and “MRPRESSO” within R software (version 4.4.0).

### Mapping SNPs to genes and transcriptomic MR analysis

To further investigate the biologically meaningful links between GM and brain white matter, we selected IVs from statistically significant bacterial taxa. These IVs, representing genetic variants, were then mapped to genes using the SNPGENE tool from FUMA method (Watanabe et al., 2017). For the mapped genes, cis-eQTL data were sourced from the eQTLGEN Consortium, which includes 16,987 genes from 31,684 blood samples of healthy European individuals across 37 datasets (http://eqtlgen.org). Subsequently, we performed a transcriptomic MR analysis. To avoid excluding potentially causal variants, we used an LD-based clumping method with an *R*^2^ < 0.01 for the eQTLs within a 10,000 kb window. The *F* statistic (*F* = beta^2^/se^2^) was calculated for each SNP, and only strong IVs with *F* > 10 were retained for the MR analysis to assess their relationship with brain white matter. Statistical significance was assessed using Bonferroni correction, with a threshold of *p* < 0.05/*n* (where *n* is the number of genes associated with each bacterium).

### SMR analysis and HEIDI test

In comparison with other methods for integrating GWAS and eQTL data, the summary Mendelian randomization (SMR) and Heterogeneity in Dependent Instruments (HEIDI) approach is distinctive in its ability to distinguish pleiotropic effects and genetic linkage (Zhu et al., 2016). In this study, SMR analysis was used as an additional tool to assess and validate the causal relationship between gene expression and brain white matter. The SMR method, implemented in the SMR software (version 1.3.1), enabled a robust exploration of these causal associations. To eliminate the potential influence of genetic linkage, the HEIDI test was applied. A significant SMR result was determined after Bonferroni correction for multiple testing, where a *p* < 0.05/*n* (with *n* representing the number of significant genes associated with each outcome in the MR analysis) was considered significant. Additionally, a HEIDI *p* greater than 0.05 indicated that the observed association was likely due to a shared genetic variant. This complementary analysis strengthened the causal relationships identified in the main MR analysis, providing more reliable evidence for the association between gene expression and brain white matter.

## Result

### Association between genetically predicted GM, WMH, and white matter microstructure

**Figure 2.**
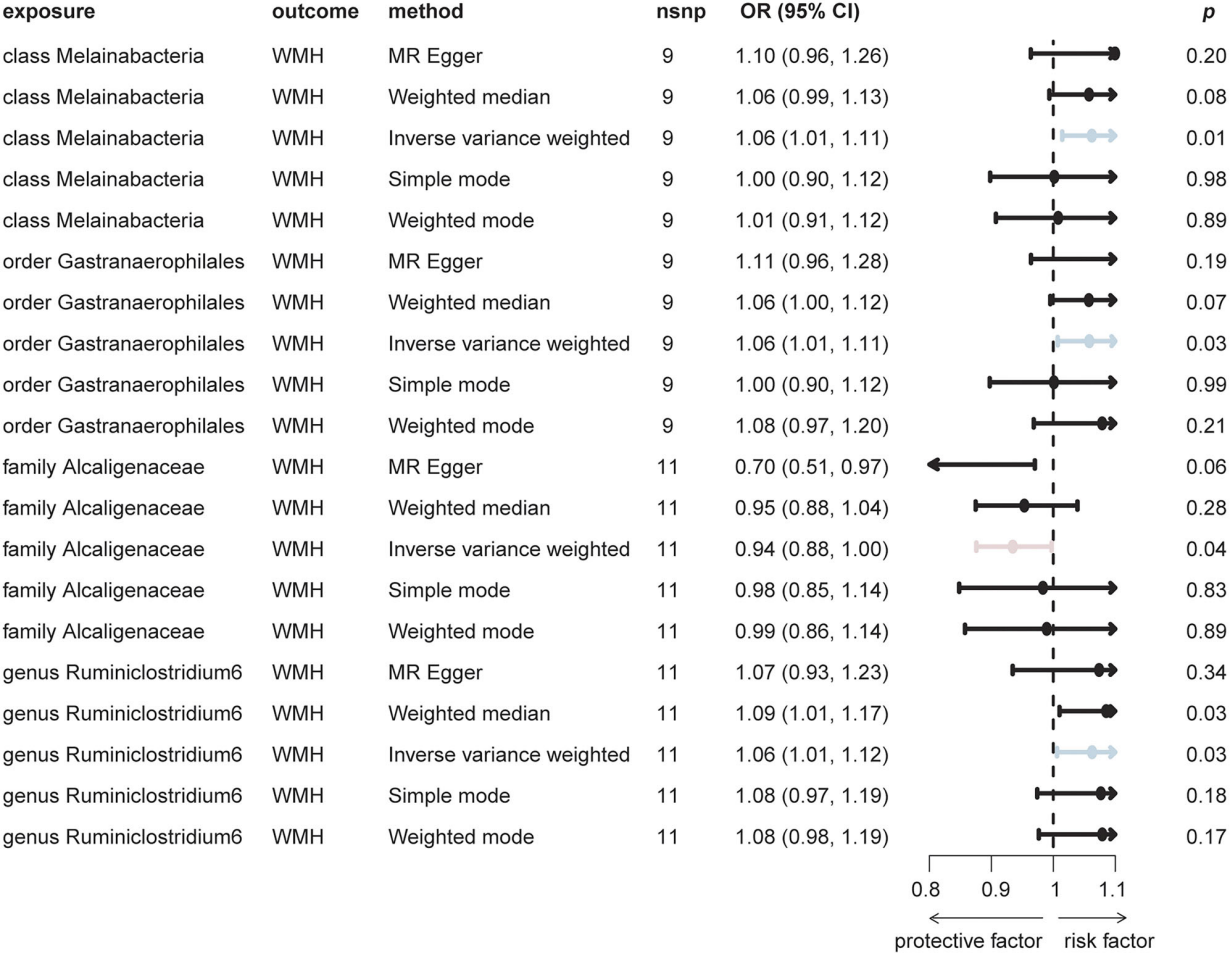
Forest plot showing the association between GM and WMH using MR method. Nominally significant associations (*p* < 0.05) are observed. Extended Data Figure 2-1 provides details of heterogeneity and horizontal pleiotropy tests evaluating the GM and WMH causal relationship. nsnp, number of SNP; CI, confidence interval; IVW, inverse-variance weighted; WMH, white matter hyperintensity.

MR analysis was carried out to investigate the causal effects of 196 genetically predicted bacterial taxa on WMH and white matter microstructure. The analysis revealed several associations; however, none of the results reached statistical significance. Additionally, nine results showed potential pleiotropy. After further analysis using MR-PRESSO, it was found that four of these associations lacked statistical significance. For WMH (Fig. 2, Extended Data Fig. 2-1), we found that higher levels of the class *Melainabacteria* (*β* = 0.06, OR [95% CI] = 1.06 [1.01, 1.11], *p* = 0.01), order *Gastranaerophilales* (*β* = 0.06, OR [95% CI] = 1.06 [1.01, 1.11], *p* = 0.03), and genus *Ruminiclostridium 6* (*β* = 0.06, OR [95% CI] = 1.06 [1.01, 1.12], *p* = 0.03) were associated with an increased risk of WMH. In contrast, the family *Alcaligenaceae* (*β* = −0.07, OR [95% CI] = 0.94 [0.88, 1.00], *p* = 0.04) was associated with a protective effect against WMH.

10.1523/ENEURO.0586-24.2025.f2-1 Figure 2-1 Tests for heterogeneity and pleiotropy in the causal effect of GM on WMH. Download Figure 2-1, DOC file.

**Figure 3.**
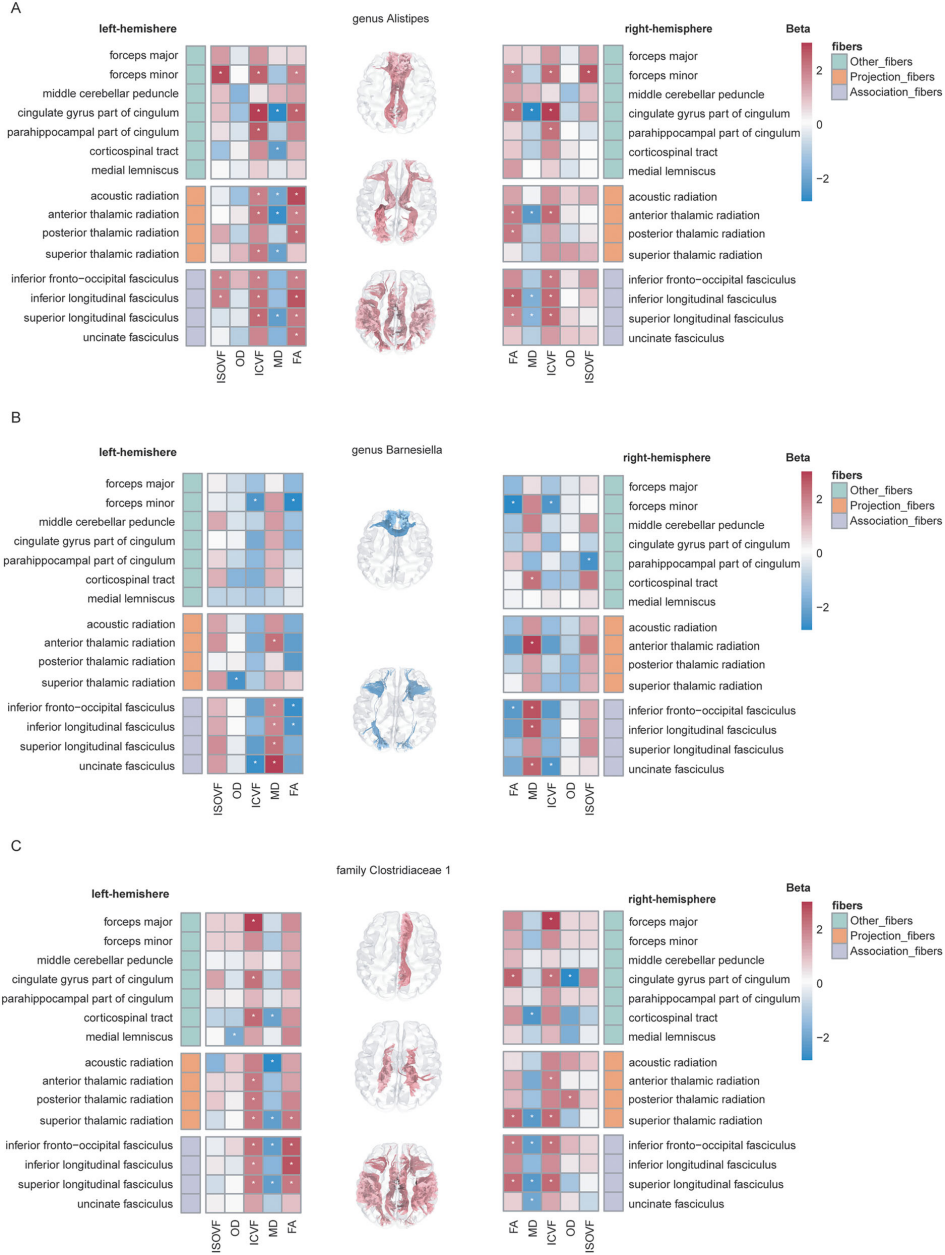
Association between three specific bacterial taxa and white matter tracts. The fiber tracts displayed in the brain maps represent those with FA values exhibiting a *p* *<* 0.05. Significant associations (*p* < 0.05) are marked with asterisks (*). Extended Data Figures 3-1 and 3-2 present the MR estimates for causal effects of three specific bacterial taxa on white matter microstructure and the corresponding heterogeneity and pleiotropy tests, respectively. FA, fractional anisotropy; MD, mean diffusivity; ICV, intracellular volume fraction; OD, orientation dispersion; ISOVF, isotropic volume fraction. ***A***, genus *Alistipes*; ***B***, genus *Barnesiella*; ***C***, family *Clostridiaceae 1*.

Fractional anisotropy (FA), mean diffusivity (MD), intracellular volume fraction (ICVF), isotropic volume fraction (ISOVF), and orientation dispersion (OD) across 27 major whiter matter tracts from DTI and NODDI metrics were used as white matter microstructure indexes, and DTI is commonly used to assess white matter microstructural integrity, with lower FA and higher MD values typically indicating compromised integrity. Specific bacterial taxa were found to have either protective or detrimental effects on white matter tracts. Our finding showed that the genetically predicted genus *Alistipes* was nominally associated with increased FA or decreased MD in 16 white matter tracts, while the family *Clostridiaceae 1* showed to be protective for 12 white matter tracts. Conversely, the genus *Barnesiella* was nominally linked to decreased FA or increased MD in 11 white matter tracts, suggesting destructive for white matter microstructural integrity (Fig. 3, Extended Data Figs. 3-1, 3-2).

10.1523/ENEURO.0586-24.2025.f3-1 Figure 3-1 Mendelian randomization estimates the causal effect of GM and white matter microstructure. Download Figure 3-1, DOC file.

10.1523/ENEURO.0586-24.2025.f3-2 Figure 3-2 Tests for heterogeneity and pleiotropy in the causal effect of GM on white matter microstructure. Download Figure 3-2, DOC file.

### Causal effect of GM on white matter connectivity

**Figure 4.**
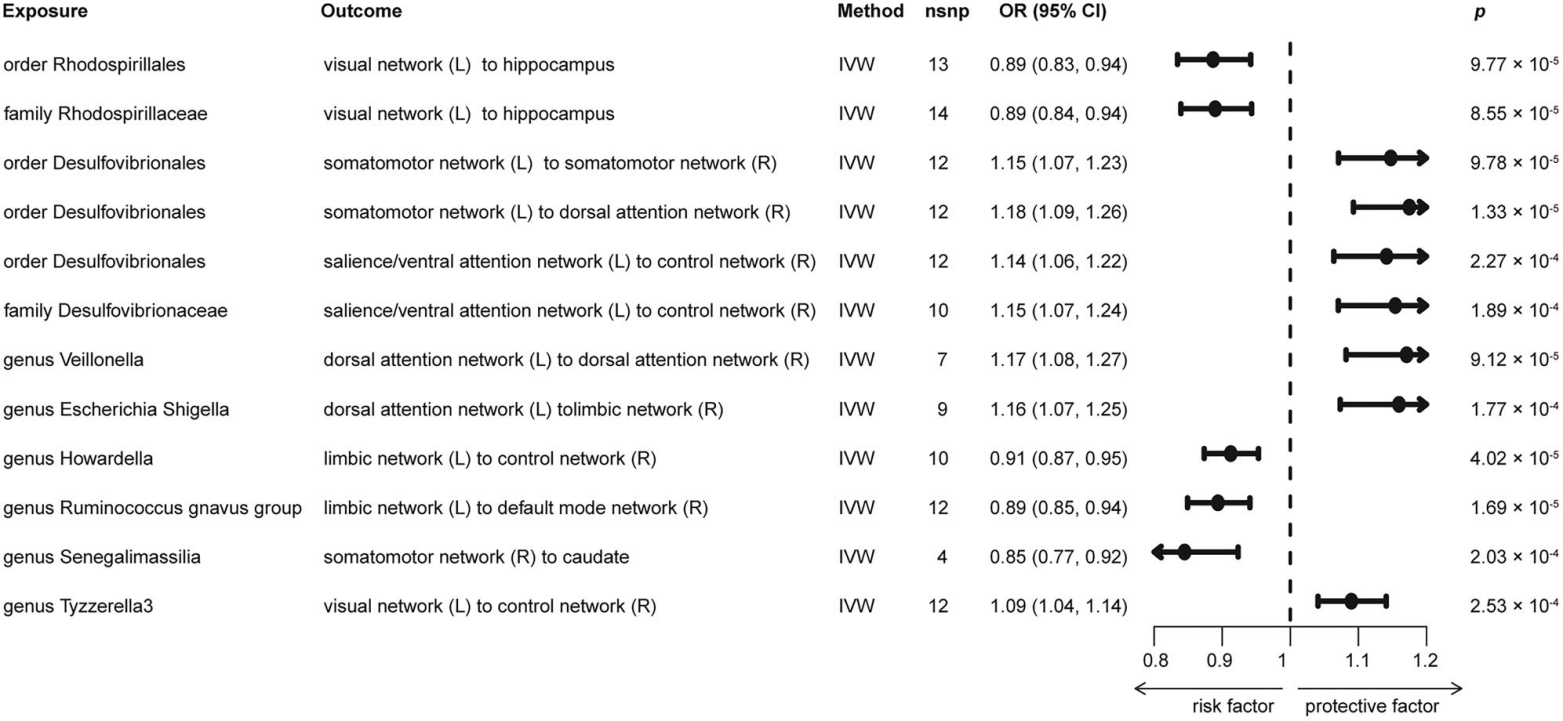
The MR results displaying the association between GM and white matter structural connectivity, adjusted for multiple testing using Bonferroni’s correction. Extended Data Figure 4-1 provides details of heterogeneity and horizontal pleiotropy tests evaluating the GM and WMH causal relationship. L, left-hemisphere; R, right-hemisphere.

We next explored the causal impact of genetically predicted 196 bacterial taxa on 206 white matter connectivity measures. In total, 1,676 nominally significant causal associations and 12 strong associations between genetic liability in the GM and white matter connectivity were identified through Bonferroni correction (Fig. 4, Extended Data Fig. 4-1). However, among these, 15 results showed potential pleiotropy. After further analysis using MR-PRESSO, it was found that 10 of these associations lacked statistical significance. Specifically, the order *Rhodospirillales* (*β* = −0.12, OR [95% CI] = 0.89 [0.83, 0.94], *p* = 9.77 × 10^−5^) and family *Rhodospirillaceae* (*β* = −0.12, OR [95% CI] = 0.89 [0.84, 0.94], *p* = 8.55 × 10^−5^) were associated with a risk effect on the connectivity between the left-hemisphere visual network and hippocampus. Additionally, the genus *Tyzzerella 3* (*β* = 0.09, OR [95% CI] = 0.89 [0.83, 0.94], *p* = 9.77 × 10^−5^) showed a risk effect on the connectivity between the left-hemisphere visual network and right-hemisphere control network, and the genus *Howardella* (*β* = −0.09, OR [95% CI] = 0.91 [0.87, 0.95], *p* = 1.77 × 10^−4^) was associated with a risk effect on the connectivity between the left-hemisphere limbic network and right-hemisphere control network. The genus *Ruminococcus gnavus group* (*β* = −0.11, OR [95% CI] = 0.89 [0.85, 0.94], *p* = 1.68 × 10^−5^) was also linked to a risk effect on the connectivity between the left-hemisphere limbic network and right-hemisphere default mode network. In contrast, the order *Desulfovibrionales* was consistently linked with an increased protection for white matter connectivity alterations across multiple measures, including connections between the left-hemisphere somatomotor network and both the right-hemisphere somatomotor network (*β* = 0.14, OR [95% CI] = 1.15 [1.07, 1.23], *p* = 9.77 × 10^−5^) and the right-hemisphere dorsal attention network (*β* = 0.16, OR [95% CI] = 1.18 [1.09, 1.26], *p* = 1.32 × 10^−5^), as well as between the left-hemisphere salience/ventral attention network and right-hemisphere control network (*β* = 0.13, OR [95% CI] = 1.14 [1.06, 1.22], *p* = 2.27 × 10^−4^). Additionally, the family *Desulfovibrionaceae* (*β* = 0.14, OR [95% CI] = 1.15 [1.07, 1.24], *p* = 1.88 × 10^−4^) was identified as a protective factor for connectivity between the left-hemisphere salience/ventral attention network and right-hemisphere control network, while the genus *Veillonella* (*β* = 0.16, OR [95% CI] = 1.17 [1.08, 1.27], *p* = 9.12 × 10^−5^) was linked to alterations in connectivity between the left-hemisphere dorsal attention network and right-hemisphere dorsal attention network. The genus *Escherichia-Shigella* (*β* = 0.148, OR [95% CI] = 1.16 [1.07, 1.25], *p* = 1.77 × 10^−4^) was associated with changes in connectivity between the left-hemisphere dorsal attention network and right-hemisphere limbic network, and the genus *Senegalimassilia* (*β* = −0.17, OR [95% CI] = 0.85 [0.77, 0.92], *p* = 2.02 × 10^−4^) was linked to connectivity alterations between the right-hemisphere somatomotor network and caudate. These findings help in understanding the complex and diverse roles that different gut taxa may play in modulating white matter connectivity.

10.1523/ENEURO.0586-24.2025.f4-1 Figure 4-1 Tests for heterogeneity and pleiotropy in the causal effect of GM on white matter connectivity. Download Figure 4-1, DOC file.

### Causal effect of selected GM and neurological diseases

**Figure 5.**
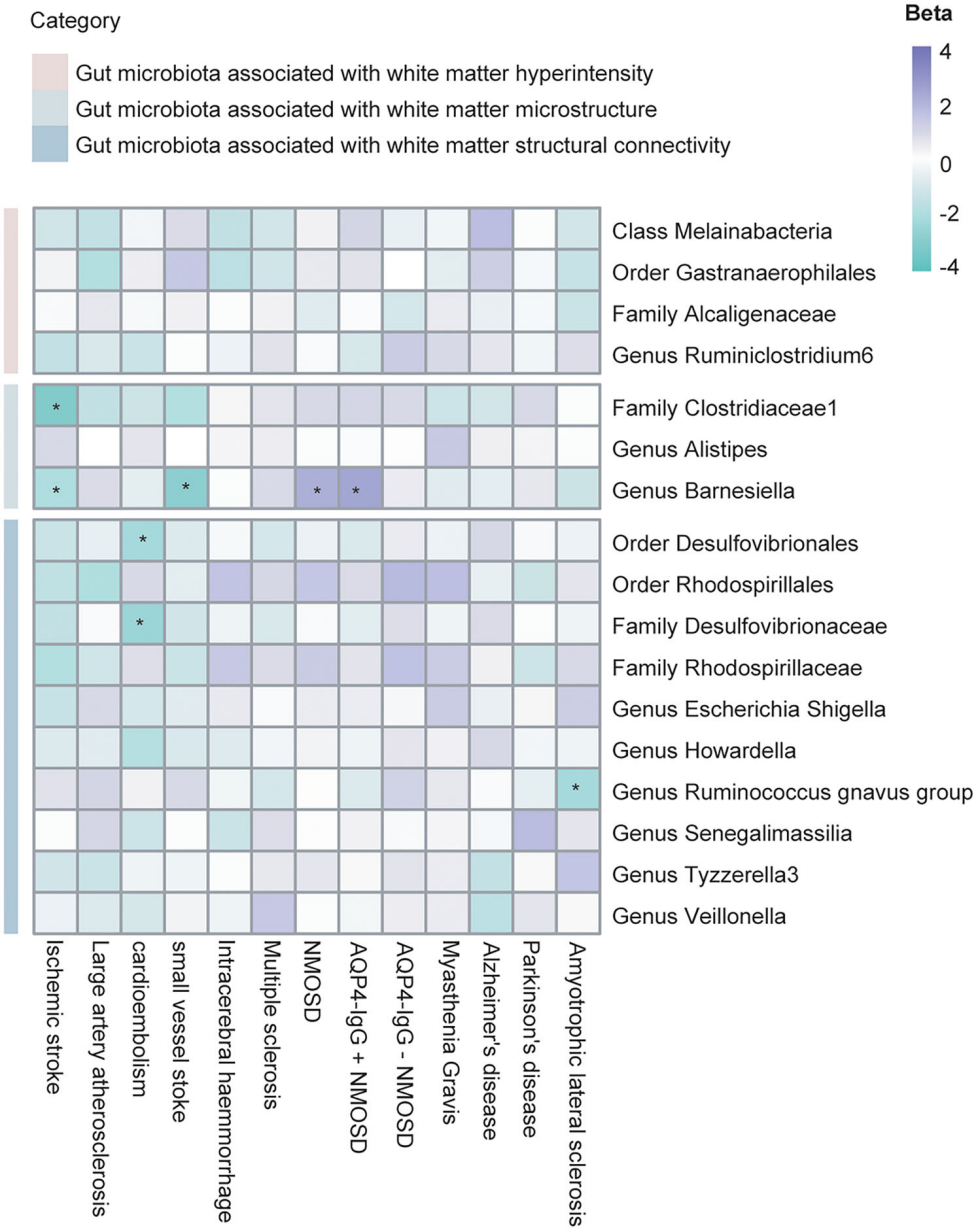
Heatmap displaying key gut microbiota associated with various neurological diseases. Significant associations (*p* < 0.05) are marked with asterisks (*). Extended Data Figures 5-1 and 5-2 illustrate the MR estimates of causal effects and the corresponding heterogeneity and pleiotropy tests for 17 selected bacterial taxa and 13 neurological diseases.

We identified four WMH-related bacterial taxa. In addition, three bacterial taxa were discovered that have consistent effect on multiple aspects of white matter microstructure, and 10 bacterial taxa were strongly associated with white matter connectivity. We next explored the causal impact of genetically predicted aforementioned 17 bacterial taxa on 13 neurological diseases. In total, 13 significant positive results were observed, with none showing pleiotropy (Fig. 5, Extended Data Figs. 5-1, 5-2). Among these bacterial taxa, the family *Clostridiaceae 1* demonstrated a protective effect against IS (*β* = −0.18, OR [95% CI] = 0.84 [0.75, 0.94], *p* = 2.03 × 10^−3^). The genus *Barnesiella* showed protective effect both in IS (*β* = −0.11, OR [95% CI] = 0.90 [0.81, 1.00], *p* = 0.04) and small vessel stroke (SVS; *β* = −0.33, OR [95% CI] = 0.72 [0.58, 0.90], *p* = 4.59 × 10^−3^) but posed a risk effect on neuromyelitis optica spectrum disorder (NMOSD; *β* = 1.08, OR [95% CI] = 2.95 [1.09, 7.98], *p* = 0.03) and aquaporin-4 immunoglobulin G-positive neuromyelitis optica spectrum disorder (AQP4-IgG+ NMOSD; *β* = 1.54, OR [95% CI] = 4.64 [1.34, 16.11], *p* = 0.02). The order *Desulfovibrionales* showed a protective effect against cardioembolic stroke (CES; *β* = −0.23, OR [95% CI] = 0.80 [0.65, 0.98], *p* = 0.03), as well as the family *Desulfovibrionaceae* (*β* = −0.27, OR [95% CI] = 0.76 [0.61, 0.95], *p* = 0.01). The genus *Ruminococcus gnavus group* showed a protective effect on amyotrophic lateral sclerosis (ALS; *β* = −0.12, OR [95% CI] = 0.88 [0.79, 0.99], *p* = 0.03). The order *Desulfovibrionales*, family *Desulfovibrionaceae*, and family *Clostridiaceae 1* have consistent effects on white matter-related indexes and diseases.

10.1523/ENEURO.0586-24.2025.f5-1 Figure 5-1 Mendelian randomization estimates the causal effect of 17 selected bacterial taxa and 13 neurological diseases. Download Figure 5-1, DOC file.

10.1523/ENEURO.0586-24.2025.f5-2 Figure 5-2 Tests for heterogeneity and pleiotropy in the causal effect of 17 selected bacterial taxa on 13 neurological diseases. Download Figure 5-2, DOC file.

### Mapping SNPs to genes and transcriptomic MR analysis

**Figure 6.**
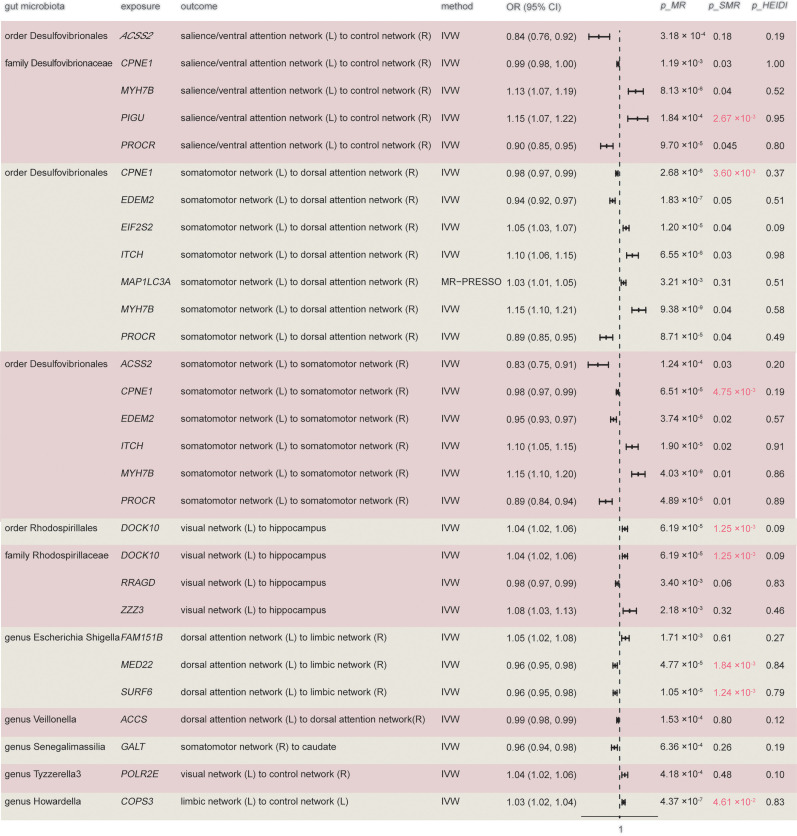
MR and SMR analysis of bacterial taxa-mapped genes and their association with white matter connectivity. Extended Data Figure 6-1 catalogs the genes mapped using the FUMA tool for 10 bacterial taxa. Extended Data Figure 6-2 details the MR-derived causal relationships between bacterial taxa-mapped genes and white matter connectivity, and Extended Data Figure 6-3 presents the corresponding heterogeneity and horizontal pleiotropy analyses. Extended Data Figure 6-4 displays the SMR analysis results of these bacterial taxa-mapped genes and their associations with white matter connectivity. L, left-hemisphere; R, right-hemisphere.

In the previous analysis, only the order *Rhodospirillales*, family *Rhodospirillaceae*, genus *Tyzzerella 3*, genus *Howardella*, genus *Ruminococcus gnavus group*, order *Desulfovibrionales*, family *Desulfovibrionaceae*, genus *Veillonella*, genus *Escherichia-Shigella*, and *genus Senegalimassilia* were identified to be significantly associated with specific white matter connectivity through Bonferroni correction. Next, IVs of these 10 bacterial taxa were used to map the corresponding genes using the FUMA tool. Among the mapped genes, SNPs associated with 273 genes were obtained from the eQTLGen consortium (Extended Data Fig. 6-1). Transcriptomic MR analysis was then performed on the mapped genes of the 10 specific bacterial taxa and the associated white matter connectivity. A total of 20 genes were identified to be associated with white matter connectivity. One of the results exhibited pleiotropy, but after MR-PRESSO analysis, it remained statistically significant (Fig. 6, Extended Data Figs. 6-2, 6-3). To further eliminate the effects of pleiotropy and linkage disequilibrium, we conducted SMR and HEIDI tests to validate the results (Fig. 6, Extended Data Fig. 6-4), Genes such as *PIGU* from the order *Desulfovibrionales* and family *Desulfovibrionaceae*, *CPNE1* from the order *Desulfovibrionales*, *COPS3* from the genus *Howardella*, *MED22* and *SURF6* from the genus *Escherichia-Shigella*, and *DOCK10* from the order *Rhodospirillales* and family *Rhodospirillaceae* were positively associated with white matter connectivity after Bonferroni correction.

10.1523/ENEURO.0586-24.2025.f6-1 Figure 6-1 The genes mapped using the FUMA tool for 10 bacterial taxa. Download Figure 6-1, DOC file.

10.1523/ENEURO.0586-24.2025.f6-2 Figure 6-2 MR analysis of bacterial taxa-mapped genes and their association with white matter connectivity. Download Figure 6-2, DOC file.

10.1523/ENEURO.0586-24.2025.f6-3 Figure 6-3 Tests for heterogeneity and pleiotropy in the causal effect of bacterial taxa-mapped genes and their association with white matter connectivity. Download Figure 6-3, DOC file.

10.1523/ENEURO.0586-24.2025.f6-4 Figure 6-4 SMR analysis of bacterial taxa-mapped genes and their association with white matter connectivity. Download Figure 6-4, DOC file.

## Discussion

In this study, we performed MR analyses to investigate the causal relationships between GM and WMH, white matter microstructure, white matter connectivity, and multiple neurological diseases. We identified specific bacterial taxa that were significantly associated with white matter alterations.

WMHs are associated with multiple neurological disorders, including cognitive decline, dementia, stroke, while specific bacterial taxa associated with WMH are still uncertain (Wardlaw et al., 2015; Graff-Radford et al., 2019; Garnier-Crussard et al., 2023). A cross-sectional study, involving 832 controls and 136 patients, tried to investigate the WMH-related gut dysbiosis, referring to *Proteobacteria*, *Candidate Phylum OD1*, *Firmicutes*, *Actinobacteria*, and *Bacteroidetes* at the phylum level; *Christensenella*, *Barnesiella*, *Ruminococcus*, *Methanobrevibacter*, *Clostridium cc_115*, and *Oscillospira* at the genus level; and *aldenense*, *intestinihominis*, *lactaris*, and *ruminantium* at the species level (Fongang et al., 2023). However, this observational study only suggested a correlational relationship, while we conducted a causal analysis using a larger sample from the UK database and extended three taxa—class *Melainabacteria*, order *Gastranaerophilales*, and genus *Ruminiclostridium 6*—as risk factors for WMH, while the family *Alcaligenaceae* was found to be a protective factor. *Gastranaerophilales*, a major order within the class *Melainabacteria* (Hu and Rzymski, 2022), can synthesize a nonessential neurotoxic amino acid, β-methylamino-ʟ-alanine (BMAA; Cox et al., 2005), which is considered a potential etiological factor in neurodegenerative processes and diseases (Brenner, 2013) and is consistent with our MR analysis results. It is noteworthy that the lack of reports on the family *Alcaligenaceae* and brain white matter and neurological diseases suggests the attentional potential of the family *Alcaligenaceae* in the central nervous system. Our study provides important insights into the association between the GM and WHM, but further studies are needed to replicate the results.

Our studies also demonstrated that the genus *Alistipes* and the family *Clostridiaceae 1* have protective effects on specific white matter tracts. And it was found that the family *Clostridiaceae 1* can reduce the risk of IS in our study. The genus *Barnesiella* revealed detrimental effects on specific white matter tracts. In poststroke depression patients, the genus *Barnesiella* was significantly increased and positively correlated with higher depression scores (Yao et al., 2023). It showed a protective effect on IS and SVS but posed a risk effect on NMOSD and AQP4-IgG+ NMOSD. This highlights the complexity of GM, as the same genus may play different roles in various diseases.

Meanwhile, the order *Rhodospirillales*, family *Rhodospirillaceae*, genus *Tyzzerella 3*, genus *Howardella*, genus *Ruminococcus gnavus group*, order *Desulfovibrionales*, family *Desulfovibrionaceae*, genus *Veillonella*, genus *Escherichia-Shigella*, and genus *Senegalimassilia* were causally associated with white matter connectivity. The family *Desulfovibrionaceae* is a group within the order *Desulfovibrionales*. These bacterial taxa are known for their ability to reduce sulfate to hydrogen sulfide (H2S) through the sulfate reduction pathway, playing a crucial role in the sulfur cycle (Carbonero et al., 2012). The H2S produced by these bacterial taxa can have toxic effects on intestinal epithelial cells, potentially contributing to or exacerbating inflammation (Verstreken et al., 2012). Previous study found that the abundant of *Desulfovibrio* in fecal samples were increased in PD and MG (Moris et al., 2018; Murros et al., 2021). However, the MR analysis revealed that *Desulfovibrionales* is associated with a reduced risk of CES and as a protective factor for white matter connectivity in this study. A recent study suggests that *Desulfovibrio* may be an effective short-chain fatty acid (SCFA)-producing bacterium that can alleviate nonalcoholic fatty liver disease in mice (Hong et al., 2021). *Escherichia-Shigella* species are Gram-negative pro-inflammatory pathogens that disrupt the intestinal mucosal barrier. This disruption facilitates the entry of lipopolysaccharides (LPSs) into the bloodstream, triggering inflammatory signaling pathways. Consequently, the release of pro-inflammatory cytokines is amplified, the blood–brain barrier's permeability increases, and neuroinflammation is activated (Cattaneo et al., 2017; Lin et al., 2019).

In this study, it was found that the genera *Ruminiclostridium 6*, *Tyzzerella 3*, *Veillonella*, *Ruminococcus gnavus group*, *Barnesiella*, and *Alistipes* and the family *Clostridiaceae 1*, all producers of SCFAs, are associated with white matter integrity. SCFAs can activate regulatory T cells (Tregs), suppress the activity of Th1 and Th17 cells, and reduce the release of inflammatory cytokines such as TNF-α and IL-1β, thereby alleviating systemic inflammation (Haghikia et al., 2016). Furthermore, SCFAs can cross the blood–brain barrier, reducing microglial activation and the secretion of pro-inflammatory cytokines, thus mitigating neuroinflammation (Haase et al., 2020; Deng et al., 2025). They also influence astrocytes, promoting the secretion of brain-derived neurotrophic factor (BDNF) and vascular endothelial growth factor (VEGF), which helps inhibit demyelination, improves the microenvironment for oligodendrocyte precursor cell (OPC) differentiation, and facilitates myelin repair (Chen et al., 2019; Guo and Bi, 2024). However, in this study, the genera *Ruminiclostridium6*, *Barnesiella*, and *Ruminococcus gnavus group* were found to be detrimental to white matter microstructure or white matter connectivity. The LPS exposed on the surface of *Ruminococcus gnavus group* promote the secretion of pro-inflammatory cytokines via TLR4 (Henke et al., 2019). Previous studies have shown that *Barnesiella* is significantly positively correlated with LPS, apoptosis index,and IL-1β (Qin et al., 2024). Therefore, whether *Barnesiella*, *Ruminiclostridium6*, and *Ruminococcus gnavus group* are beneficial bacterial taxa or pathogens remains to be further investigated. The exact mechanism by why the GM affect white matter has not been determined. Therefore, a mechanistic analysis of our results is required for further investigation.

Beyond the mechanisms previously described, FUMA method provides new genetic-level insights. It identified that *CPNE1*, *PIGU*, *MED22*, *SURF6*, *DOCK10*, and *COPS3* are associated with GM and white matter connectivity. *DOCK10*, a member of the DOCK-D family, functions as an atypical guanine nucleotide exchange factor, playing a key role in regulating the activity of Rho GTPases. In the EAE model, *DOCK10*^−/−^ mice showed reduced disease severity, which was linked to the inhibition of neuroinflammation through impaired microglial migration and suppressed CCL2 upregulation in astrocytes following inflammatory stimulation (Namekata et al., 2020). *PIGU* is a key component of the glycosylphosphatidylinositol (GPI) transamidase complex, which is involved in attaching GPI anchors to proteins. Mutations in *PIGU* disrupt this process, leading to severe intellectual disability, epilepsy, and brain anomalies (Knaus et al., 2019). *CPNE1* is a well-known phospholipid-binding protein that mediates neuronal differentiation through the Akt signaling pathway (Kim et al., 2018), and it regulates neural stem cell functions during brain development. In a chronic cerebral hypoperfusion rat model, the CPNE1-NF-κB pathway ameliorated white matter injury by regulating microglia and astrocytes (Zhao et al., 2022). While the effects of *MED22*, *SURF6*, and *COPS3* risk genes on the underlying mechanism between GM and white matter connectivity remain uncertain, the results of this study offer valuable insights for future mechanistic investigations. These genes may play a role in the pathophysiological basis of GM-induced white matter connectivity.

To the best of our knowledge, this is the first MR study to establish a relationship between the GM and brain white matter, identifying special bacterial taxa with genetic responsibility for causal relationships with brain white matter. Furthermore, this study combines MR analysis with FUMA analysis to explore the causal relationship between GM and brain white matter.

Our study has some limitations. Firstly, the research focuses on participants of European ancestry, which limits the extrapolation of results; therefore, it is necessary to conduct GWAS studies about GM in other ethnic groups to verify our results. Secondly, the state of white matter is not static, and the dynamic nature of GM must be taken into account. Thirdly, our analyses rely on publicly available GWAS summary statistics, which may introduce potential biases due to differences in sample characteristics and phenotype definitions across datasets. Lastly, experimental validation is lacking and will be essential to confirm the causal relationships and elucidate the underlying biological mechanisms implied by our results.

### Conclusion

In conclusion, this two-sample MR study had established the causal relationship between GM and brain white matter and identified specific white matter-related GM. In terms of the mapped genes of statistically significant bacterial taxa, genes such as *CPNE1*, *PIGU*, *MED22*, *SURF6*, *DOCK10*, and *COPS3* exhibited a significant causal correlation with white matter connectivity. Further research is needed to better understand the underlying mechanisms between GM and brain white matter and explore whether GM modulation could be a promising therapeutic target for improving brain health and mitigating white matter damage in neurological conditions.

## Data Availability

The datasets utilized in this study are publicly accessible summary datasets, which can be located in the referenced papers.

## Synthesis

Reviewing Editor: Eran Perlson, Tel Aviv University Decisions are customarily a result of the Reviewing Editor and the peer reviewers coming together and discussing their recommendations until a consensus is reached. When revisions are invited, a fact-based synthesis statement explaining their decision and outlining what is needed to prepare a revision will be listed below. The following reviewer(s) agreed to reveal their identity: Siddharth Nanguneri.

The manuscript uses a novel approach to process data which have been deposited in the public repositories. The study's insights suggest experiments which can be conducted to further validate the efficacy of the method. As a result the study can be published as purely a simulation study. Following the concerns from the earlier review the authors have identified genes which play role in neurodegeneration. Given the predictions a biologist would still like to validate the them. As an example, the protein folding software ALphaFold does not do a great job at synthesizing a model of an antibody against an epitope. This suggests that powerful software analyses have their limitations and as a result their predictions need to be validated extensively experimentally.

Although the analysis proving causality agrees with experimental data on many occasions there are domains in which it goes in the opposite direction. Points made in Lines 293 to 299 provide a good example where the analysis has proven contrary to the experimental data. This evidence argues for a need to support any form of analysis with experimental data, though the authors have done a good job coming with an extensive set of analysis, at the end of the day it still needs support from experimental data.

Authors have done a great deal of work to address many concerns put forth in the previous review. They have provided a list genes using FUMA.

The authors rightly point out in the conclusion that "Further research is needed to better understand the underlying mechanisms between GM and brain white matter, and explore whether GM modulation could be a promising therapeutic target for improving brain health and mitigating white matter damage in neurological conditions.

There are many grammatical errors.

Lines 49 to 52

The gut-brain-axis was proved to one crucial pathway that permitting GM exert substantial influence on host brain function, operating via three primary pathways: immune modulation, neuroendocrine signaling, and vagus nerve communication(Collins SM et al., 2012;Morais LH et al., 2021).

More appropriately:

The gut-brain-axis has proved to be a crucial pathway by which GM exert substantial influence on host brain function principally operating via three primary pathways: immune modulation, neuroendocrine signaling, and vagus nerve communication(Collins SM et al., 2012;Morais LH et al., 2021).

Lines 57 to 59:

Growing evidence indicates that oligodendrocyte generation and 58 Alzheimer's disease (AD), Parkinson's disease (PD) (Cryan JF et al., 2020;Dong S et al., 59 2022;Zhao Y et al., 2015).

The above sentence is incomplete.

Lines 47 to 49

A connection between enteric nervous system and the central nervous system, commonly known as the 49 "gut-brain axis" (Gawey BJ et al., 2025)

Incomplete statement

There are quite a few incomplete sentences and grammatical errors scattered throughout the paper which require attention.
