# Supplementary material for: Gut Microbiota and White Matter Integrity: A Two-Sample Mendelian Randomization Analysis
Source: eNeuro. 2025 Aug 29;12(9):ENEURO.0586-24.2025. doi: 10.1523/ENEURO.0586-24.2025 (PMC12418065; doi:10.1523/ENEURO.0586-24.2025)
Supplement: Figure 2-1 — Tests for heterogeneity and pleiotropy in the causal effect of GM on WMH. Download Figure 2-1, DOC file. [file eneuro-12-ENEURO.0586-24.2025-s004.doc]

Figure 2-1

Tests for heterogeneity and pleiotropy in the causal effect of gut microbiome on WMH

| Mendelian randomization | | Sensitivity analysis | |
| --- | --- | --- | --- |
| Exposure | Outcome | *p* (Heterogeneity test) | *p* (Pleiotropy test) |
| class Melainabacteria | WMH | 0.32 | 0.60 |
| family Alcaligenaceae | WMH | 0.81 | 0.11 |
| genus Ruminiclostridium 6 | WMH | 0.70 | 0.87 |
| order Gastranaerophilales | WMH | 0.22 | 0.50 |
