# Supplementary material for: Gut Microbiota and White Matter Integrity: A Two-Sample Mendelian Randomization Analysis
Source: eNeuro. 2025 Aug 29;12(9):ENEURO.0586-24.2025. doi: 10.1523/ENEURO.0586-24.2025 (PMC12418065; doi:10.1523/ENEURO.0586-24.2025)
Supplement: Figure 3-1 — Mendelian randomization estimates the causal effect of GM and white matter microstructure. Download Figure 3-1, DOC file. [file eneuro-12-ENEURO.0586-24.2025-s005.doc]

Figure 3-1

Mendelian randomization estimates the causal effect of GM and white matter microstructure

| Exposure | Outcome | Method | *p* | OR (95% CI) |
| --- | --- | --- | --- | --- |
| genus Alistipes | FA ar l | MR Egger | 0.60 | 1.15 (0.69, 1.92) |
| genus Alistipes | FA ar l | Weighted median | 0.14 | 1.12 (0.97, 1.29) |
| genus Alistipes | FA ar l | IVW | 3.05 × 10^-3^ | 1.17 (1.06, 1.31) |
| genus Alistipes | FA ar l | Simple mode | 0.53 | 1.09 (0.85, 1.40) |
| genus Alistipes | FA ar l | Weighted mode | 0.60 | 1.07 (0.84, 1.35) |
| genus Alistipes | FA atr l | MR Egger | 0.26 | 1.46 (0.78, 2.74) |
| genus Alistipes | FA atr l | Weighted median | 0.23 | 1.10 (0.94, 1.30) |
| genus Alistipes | FA atr l | IVW | 0.04 | 1.14 (1.00, 1.30) |
| genus Alistipes | FA atr l | Simple mode | 0.39 | 1.12 (0.87, 1.46) |
| genus Alistipes | FA atr l | Weighted mode | 0.48 | 1.10 (0.85, 1.41) |
| genus Alistipes | FA atr r | MR Egger | 0.25 | 1.49 (0.78, 2.84) |
| genus Alistipes | FA atr r | Weighted median | 0.23 | 1.10 (0.94, 1.27) |
| genus Alistipes | FA atr r | IVW | 0.02 | 1.17 (1.02, 1.33) |
| genus Alistipes | FA atr r | Simple mode | 0.44 | 1.10 (0.87, 1.40) |
| genus Alistipes | FA atr r | Weighted mode | 0.43 | 1.10 (0.88, 1.37) |
| genus Alistipes | FA cgc l | MR Egger | 0.67 | 1.12 (0.67, 1.87) |
| genus Alistipes | FA cgc l | Weighted median | 0.01 | 1.20 (1.04, 1.39) |
| genus Alistipes | FA cgc l | IVW | 0.01 | 1.15 (1.03, 1.28) |
| genus Alistipes | FA cgc l | Simple mode | 0.07 | 1.30 (1.00, 1.70) |
| genus Alistipes | FA cgc l | Weighted mode | 0.07 | 1.30 (1.01, 1.68) |
| family Clostridiaceae1 | FA cgc r | MR Egger | 0.11 | 1.32 (0.98, 1.77) |
| family Clostridiaceae1 | FA cgc r | Weighted median | 0.07 | 1.14 (0.99, 1.33) |
| family Clostridiaceae1 | FA cgc r | IVW | 0.01 | 1.15 (1.03, 1.27) |
| family Clostridiaceae1 | FA cgc r | Simple mode | 0.30 | 1.14 (0.90, 1.44) |
| family Clostridiaceae1 | FA cgc r | Weighted mode | 0.27 | 1.14 (0.92, 1.41) |
| genus Alistipes | FA cgc r | MR Egger | 0.35 | 1.36 (0.73, 2.52) |
| genus Alistipes | FA cgc r | Weighted median | 3.93× 10^-3^ | 1.24 (1.07, 1.44) |
| genus Alistipes | FA cgc r | IVW | 0.02 | 1.17 (1.03, 1.32) |
| genus Alistipes | FA cgc r | Simple mode | 0.06 | 1.28 (1.01, 1.62) |
| genus Alistipes | FA cgc r | Weighted mode | 0.07 | 1.28 (1.00, 1.63) |
| genus Alistipes | FA fmi | MR Egger | 0.95 | 1.02 (0.59, 1.77) |
| genus Alistipes | FA fmi | Weighted median | 0.07 | 1.15 (0.99, 1.33) |
| genus Alistipes | FA fmi | IVW | 0.04 | 1.12 (1.01, 1.25) |
| genus Alistipes | FA fmi | Simple mode | 0.21 | 1.16 (0.93, 1.45) |
| genus Alistipes | FA fmi | Weighted mode | 0.21 | 1.16 (0.93, 1.44) |
| genus Barnesiella | FA fmi | MR Egger | 0.30 | 1.18 (0.87, 1.59) |
| genus Barnesiella | FA fmi | Weighted median | 0.23 | 0.93 (0.82, 1.05) |
| genus Barnesiella | FA fmi | IVW | 0.02 | 0.90 (0.82, 0.98) |
| genus Barnesiella | FA fmi | Simple mode | 0.78 | 0.97 (0.78, 1.20) |
| genus Barnesiella | FA fmi | Weighted mode | 0.87 | 0.98 (0.80, 1.20) |
| family Clostridiaceae1 | FA ifo l | MR Egger | 0.12 | 1.30 (0.97, 1.76) |
| family Clostridiaceae1 | FA ifo l | Weighted median | 0.10 | 1.13 (0.98, 1.31) |
| family Clostridiaceae1 | FA ifo l | IVW | 0.01 | 1.15 (1.04, 1.28) |
| family Clostridiaceae1 | FA ifo l | Simple mode | 0.59 | 1.07 (0.85, 1.34) |
| family Clostridiaceae1 | FA ifo l | Weighted mode | 0.39 | 1.10 (0.90, 1.34) |
| genus Alistipes | FA ifo l | MR Egger | 0.48 | 1.22 (0.72, 2.06) |
| genus Alistipes | FA ifo l | Weighted median | 0.15 | 1.12 (0.96, 1.30) |
| genus Alistipes | FA ifo l | IVW | 0.04 | 1.12 (1.01, 1.24) |
| genus Alistipes | FA ifo l | Simple mode | 0.15 | 1.23 (0.94, 1.60) |
| genus Alistipes | FA ifo l | Weighted mode | 0.20 | 1.20 (0.92, 1.57) |
| genus Barnesiella | FA ifo l | MR Egger | 0.44 | 1.13 (0.84, 1.52) |
| genus Barnesiella | FA ifo l | Weighted median | 0.08 | 0.89 (0.79, 1.01) |
| genus Barnesiella | FA ifo l | IVW | 0.03 | 0.90 (0.82, 0.99) |
| genus Barnesiella | FA ifo l | Simple mode | 0.27 | 0.88 (0.70, 1.09) |
| genus Barnesiella | FA ifo l | Weighted mode | 0.31 | 0.88 (0.70, 1.12) |
| family Clostridiaceae1 | FA ifo r | MR Egger | 0.08 | 1.36 (1.01, 1.83) |
| family Clostridiaceae1 | FA ifo r | Weighted median | 0.09 | 1.14 (0.98, 1.31) |
| family Clostridiaceae1 | FA ifo r | IVW | 0.03 | 1.12 (1.01, 1.25) |
| family Clostridiaceae1 | FA ifo r | Simple mode | 0.14 | 1.22 (0.96, 1.54) |
| family Clostridiaceae1 | FA ifo r | Weighted mode | 0.15 | 1.20 (0.96, 1.50) |
| genus Barnesiella | FA ifo r | MR Egger | 0.38 | 1.15 (0.85, 1.55) |
| genus Barnesiella | FA ifo r | Weighted median | 0.19 | 0.91 (0.80, 1.05) |
| genus Barnesiella | FA ifo r | IVW | 0.05 | 0.91 (0.84, 1.00) |
| genus Barnesiella | FA ifo r | Simple mode | 0.18 | 0.84 (0.67, 1.06) |
| genus Barnesiella | FA ifo r | Weighted mode | 0.31 | 0.88 (0.70, 1.11) |
| family Clostridiaceae1 | FA ilf l | MR Egger | 0.12 | 1.31 (0.97, 1.76) |
| family Clostridiaceae1 | FA ilf l | Weighted median | 0.03 | 1.17 (1.01, 1.35) |
| family Clostridiaceae1 | FA ilf l | IVW | 0.01 | 1.16 (1.04, 1.29) |
| family Clostridiaceae1 | FA ilf l | Simple mode | 0.21 | 1.18 (0.93, 1.51) |
| family Clostridiaceae1 | FA ilf l | Weighted mode | 0.12 | 1.20 (0.97, 1.48) |
| genus Alistipes | FA ilf l | MR Egger | 0.56 | 1.17 (0.70, 1.97) |
| genus Alistipes | FA ilf l | Weighted median | 0.12 | 1.13 (0.97, 1.31) |
| genus Alistipes | FA ilf l | IVW | 4.35 × 10^-3^ | 1.17 (1.05, 1.30) |
| genus Alistipes | FA ilf l | Simple mode | 0.68 | 1.06 (0.80, 1.40) |
| genus Alistipes | FA ilf l | Weighted mode | 0.64 | 1.07 (0.82, 1.39) |
| genus Barnesiella | FA ilf l | MR Egger | 0.43 | 1.13 (0.84, 1.53) |
| genus Barnesiella | FA ilf l | Weighted median | 0.07 | 0.89 (0.79, 1.01) |
| genus Barnesiella | FA ilf l | IVW | 0.04 | 0.91 (0.84, 1.00) |
| genus Barnesiella | FA ilf l | Simple mode | 0.13 | 0.83 (0.67, 1.04) |
| genus Barnesiella | FA ilf l | Weighted mode | 0.15 | 0.84 (0.67, 1.05) |
| genus Alistipes | FA ilf r | MR Egger | 0.29 | 1.40 (0.77, 2.53) |
| genus Alistipes | FA ilf r | Weighted median | 0.25 | 1.10 (0.94, 1.28) |
| genus Alistipes | FA ilf r | IVW | 0.01 | 1.17 (1.04, 1.32) |
| genus Alistipes | FA ilf r | Simple mode | 0.66 | 1.06 (0.82, 1.37) |
| genus Alistipes | FA ilf r | Weighted mode | 0.64 | 1.06 (0.83, 1.35) |
| genus Alistipes | FA ptr l | MR Egger | 0.32 | 1.32 (0.78, 2.23) |
| genus Alistipes | FA ptr l | Weighted median | 0.02 | 1.19 (1.02, 1.39) |
| genus Alistipes | FA ptr l | IVW | 0.02 | 1.14 (1.02, 1.27) |
| genus Alistipes | FA ptr l | Simple mode | 0.15 | 1.26 (0.94, 1.68) |
| genus Alistipes | FA ptr l | Weighted mode | 0.12 | 1.25 (0.96, 1.63) |
| genus Alistipes | FA ptr r | MR Egger | 0.27 | 1.36 (0.81, 2.28) |
| genus Alistipes | FA ptr r | Weighted median | 0.32 | 1.08 (0.93, 1.25) |
| genus Alistipes | FA ptr r | IVW | 0.02 | 1.13 (1.02, 1.26) |
| genus Alistipes | FA ptr r | Simple mode | 0.53 | 1.09 (0.83, 1.43) |
| genus Alistipes | FA ptr r | Weighted mode | 0.52 | 1.09 (0.85, 1.39) |
| family Clostridiaceae1 | FA slf l | MR Egger | 0.16 | 1.27 (0.94, 1.71) |
| family Clostridiaceae1 | FA slf l | Weighted median | 0.06 | 1.16 (0.99, 1.35) |
| family Clostridiaceae1 | FA slf l | IVW | 0.04 | 1.12 (1.00, 1.24) |
| family Clostridiaceae1 | FA slf l | Simple mode | 0.22 | 1.16 (0.93, 1.45) |
| family Clostridiaceae1 | FA slf l | Weighted mode | 0.16 | 1.17 (0.96, 1.44) |
| genus Alistipes | FA slf l | MR Egger | 0.28 | 1.41 (0.78, 2.57) |
| genus Alistipes | FA slf l | Weighted median | 0.57 | 1.05 (0.90, 1.22) |
| genus Alistipes | FA slf l | IVW | 0.03 | 1.14 (1.01, 1.29) |
| genus Alistipes | FA slf l | Simple mode | 0.77 | 1.04 (0.80, 1.35) |
| genus Alistipes | FA slf l | Weighted mode | 0.80 | 1.03 (0.81, 1.31) |
| family Clostridiaceae1 | FA slf r | MR Egger | 0.12 | 1.37 (0.96, 1.95) |
| family Clostridiaceae1 | FA slf r | Weighted median | 0.02 | 1.19 (1.03, 1.38) |
| family Clostridiaceae1 | FA slf r | IVW | 0.02 | 1.16 (1.02, 1.32) |
| family Clostridiaceae1 | FA slf r | Simple mode | 0.26 | 1.17 (0.90, 1.52) |
| family Clostridiaceae1 | FA slf r | Weighted mode | 0.11 | 1.21 (0.98, 1.49) |
| genus Alistipes | FA slf r | MR Egger | 0.50 | 1.22 (0.69, 2.18) |
| genus Alistipes | FA slf r | Weighted median | 0.60 | 1.04 (0.89, 1.21) |
| genus Alistipes | FA slf r | IVW | 0.04 | 1.13 (1.00, 1.26) |
| genus Alistipes | FA slf r | Simple mode | 0.81 | 1.03 (0.82, 1.30) |
| genus Alistipes | FA slf r | Weighted mode | 0.81 | 1.03 (0.83, 1.26) |
| family Clostridiaceae1 | FA str l | MR Egger | 0.58 | 1.11 (0.78, 1.57) |
| family Clostridiaceae1 | FA str l | Weighted median | 0.16 | 1.12 (0.96, 1.30) |
| family Clostridiaceae1 | FA str l | IVW | 0.02 | 1.15 (1.02, 1.29) |
| family Clostridiaceae1 | FA str l | Simple mode | 0.32 | 1.14 (0.90, 1.45) |
| family Clostridiaceae1 | FA str l | Weighted mode | 0.26 | 1.13 (0.93, 1.37) |
| family Clostridiaceae1 | FA str r | MR Egger | 0.61 | 1.09 (0.79, 1.52) |
| family Clostridiaceae1 | FA str r | Weighted median | 0.37 | 1.07 (0.92, 1.24) |
| family Clostridiaceae1 | FA str r | IVW | 0.03 | 1.13 (1.01, 1.26) |
| family Clostridiaceae1 | FA str r | Simple mode | 0.13 | 1.23 (0.96, 1.58) |
| family Clostridiaceae1 | FA str r | Weighted mode | 0.52 | 1.07 (0.87, 1.32) |
| genus Alistipes | FA unc l | MR Egger | 0.54 | 1.22 (0.66, 2.26) |
| genus Alistipes | FA unc l | Weighted median | 0.06 | 1.16 (0.99, 1.36) |
| genus Alistipes | FA unc l | IVW | 0.02 | 1.15 (1.02, 1.30) |
| genus Alistipes | FA unc l | Simple mode | 0.24 | 1.17 (0.91, 1.51) |
| genus Alistipes | FA unc l | Weighted mode | 0.24 | 1.17 (0.91, 1.50) |
| family Clostridiaceae1 | MD ar l | MR Egger | 0.39 | 0.87 (0.65, 1.17) |
| family Clostridiaceae1 | MD ar l | Weighted median | 0.01 | 0.84 (0.73, 0.96) |
| family Clostridiaceae1 | MD ar l | IVW | 1.53 × 10^-3^ | 0.84 (0.76, 0.94) |
| family Clostridiaceae1 | MD ar l | Simple mode | 0.20 | 0.86 (0.69, 1.06) |
| family Clostridiaceae1 | MD ar l | Weighted mode | 0.13 | 0.85 (0.69, 1.03) |
| genus Alistipes | MD ar l | MR Egger | 0.87 | 0.96 (0.56, 1.63) |
| genus Alistipes | MD ar l | Weighted median | 0.02 | 0.84 (0.72, 0.97) |
| genus Alistipes | MD ar l | IVW | 0.04 | 0.89 (0.80, 0.99) |
| genus Alistipes | MD ar l | Simple mode | 0.09 | 0.79 (0.62, 1.02) |
| genus Alistipes | MD ar l | Weighted mode | 0.09 | 0.79 (0.61, 1.02) |
| genus Alistipes | MD atr l | MR Egger | 0.69 | 0.89 (0.50, 1.58) |
| genus Alistipes | MD atr l | Weighted median | 0.07 | 0.87 (0.75, 1.01) |
| genus Alistipes | MD atr l | IVW | 0.01 | 0.85 (0.76, 0.96) |
| genus Alistipes | MD atr l | Simple mode | 0.90 | 0.98 (0.74, 1.31) |
| genus Alistipes | MD atr l | Weighted mode | 0.96 | 0.99 (0.76, 1.30) |
| genus Barnesiella | MD atr l | MR Egger | 0.33 | 0.86 (0.63, 1.16) |
| genus Barnesiella | MD atr l | Weighted median | 0.16 | 1.09 (0.97, 1.24) |
| genus Barnesiella | MD atr l | IVW | 0.02 | 1.12 (1.02, 1.24) |
| genus Barnesiella | MD atr l | Simple mode | 0.24 | 1.15 (0.92, 1.42) |
| genus Barnesiella | MD atr l | Weighted mode | 0.28 | 1.14 (0.91, 1.42) |
| genus Alistipes | MD atr r | MR Egger | 0.60 | 0.84 (0.44, 1.59) |
| genus Alistipes | MD atr r | Weighted median | 0.12 | 0.88 (0.75, 1.04) |
| genus Alistipes | MD atr r | IVW | 4.08 × 10^-3^ | 0.83 (0.73, 0.94) |
| genus Alistipes | MD atr r | Simple mode | 0.58 | 0.92 (0.69, 1.23) |
| genus Alistipes | MD atr r | Weighted mode | 0.64 | 0.94 (0.74, 1.20) |
| genus Barnesiella | MD atr r | MR Egger | 0.65 | 0.93 (0.69, 1.25) |
| genus Barnesiella | MD atr r | Weighted median | 0.05 | 1.13 (1.00, 1.29) |
| genus Barnesiella | MD atr r | IVW | 0.01 | 1.13 (1.03, 1.23) |
| genus Barnesiella | MD atr r | Simple mode | 0.20 | 1.16 (0.93, 1.44) |
| genus Barnesiella | MD atr r | Weighted mode | 0.26 | 1.14 (0.92, 1.42) |
| genus Alistipes | MD cgc l | MR Egger | 0.65 | 0.88 (0.51, 1.52) |
| genus Alistipes | MD cgc l | Weighted median | 0.01 | 0.83 (0.71, 0.96) |
| genus Alistipes | MD cgc l | IVW | 3.20 × 10^-3^ | 0.85 (0.76, 0.95) |
| genus Alistipes | MD cgc l | Simple mode | 0.05 | 0.73 (0.54, 0.98) |
| genus Alistipes | MD cgc l | Weighted mode | 0.05 | 0.74 (0.56, 0.97) |
| genus Alistipes | MD cgc r | MR Egger | 0.83 | 0.94 (0.56, 1.58) |
| genus Alistipes | MD cgc r | Weighted median | 0.04 | 0.85 (0.73, 0.99) |
| genus Alistipes | MD cgc r | IVW | 5.08 × 10^-3^ | 0.83 (0.74, 0.92) |
| genus Alistipes | MD cgc r | Simple mode | 0.26 | 0.86 (0.66, 1.11) |
| genus Alistipes | MD cgc r | Weighted mode | 0.27 | 0.86 (0.67, 1.11) |
| family Clostridiaceae1 | MD cst l | MR Egger | 0.38 | 0.85 (0.60, 1.20) |
| family Clostridiaceae1 | MD cst l | Weighted median | 0.02 | 0.83 (0.72, 0.97) |
| family Clostridiaceae1 | MD cst l | IVW | 0.02 | 0.87 (0.78, 0.98) |
| family Clostridiaceae1 | MD cst l | Simple mode | 0.14 | 0.82 (0.65, 1.04) |
| family Clostridiaceae1 | MD cst l | Weighted mode | 0.10 | 0.83 (0.68, 1.01) |
| genus Alistipes | MD cst l | MR Egger | 0.99 | 1.00 (0.56, 1.77) |
| genus Alistipes | MD cst l | Weighted median | 0.06 | 0.87 (0.75, 1.01) |
| genus Alistipes | MD cst l | IVW | 0.02 | 0.87 (0.78, 0.98) |
| genus Alistipes | MD cst l | Simple mode | 0.33 | 0.86 (0.65, 1.15) |
| genus Alistipes | MD cst l | Weighted mode | 0.34 | 0.87 (0.67, 1.14) |
| family Clostridiaceae1 | MD cst r | MR Egger | 0.21 | 0.81 (0.60, 1.10) |
| family Clostridiaceae1 | MD cst r | Weighted median | 0.02 | 0.84 (0.73, 0.98) |
| family Clostridiaceae1 | MD cst r | IVW | 0.01 | 0.88 (0.79, 0.97) |
| family Clostridiaceae1 | MD cst r | Simple mode | 0.16 | 0.84 (0.67, 1.05) |
| family Clostridiaceae1 | MD cst r | Weighted mode | 0.12 | 0.83 (0.68, 1.03) |
| genus Barnesiella | MD cst r | MR Egger | 0.87 | 1.03 (0.76, 1.38) |
| genus Barnesiella | MD cst r | Weighted median | 0.30 | 1.07 (0.94, 1.21) |
| genus Barnesiella | MD cst r | IVW | 0.05 | 1.09 (1.00, 1.20) |
| genus Barnesiella | MD cst r | Simple mode | 0.75 | 1.04 (0.83, 1.30) |
| genus Barnesiella | MD cst r | Weighted mode | 0.84 | 1.02 (0.83, 1.26) |
| family Clostridiaceae1 | MD ifo l | MR Egger | 0.78 | 0.95 (0.67, 1.35) |
| family Clostridiaceae1 | MD ifo l | Weighted median | 0.43 | 0.94 (0.82, 1.09) |
| family Clostridiaceae1 | MD ifo l | IVW | 0.02 | 0.86 (0.76, 0.97) |
| family Clostridiaceae1 | MD ifo l | Simple mode | 0.59 | 0.94 (0.74, 1.18) |
| family Clostridiaceae1 | MD ifo l | Weighted mode | 0.74 | 0.97 (0.80, 1.17) |
| genus Barnesiella | MD ifo l | MR Egger | 0.23 | 0.82 (0.61, 1.11) |
| genus Barnesiella | MD ifo l | Weighted median | 0.49 | 1.04 (0.92, 1.18) |
| genus Barnesiella | MD ifo l | IVW | 0.05 | 1.10 (1.00, 1.21) |
| genus Barnesiella | MD ifo l | Simple mode | 0.87 | 1.02 (0.82, 1.27) |
| genus Barnesiella | MD ifo l | Weighted mode | 0.89 | 1.02 (0.81, 1.27) |
| family Clostridiaceae1 | MD ifo r | MR Egger | 0.42 | 0.87 (0.64, 1.19) |
| family Clostridiaceae1 | MD ifo r | Weighted median | 0.08 | 0.87 (0.75, 1.01) |
| family Clostridiaceae1 | MD ifo r | IVW | 0.03 | 0.89 (0.80, 0.99) |
| family Clostridiaceae1 | MD ifo r | Simple mode | 0.07 | 0.76 (0.59, 0.99) |
| family Clostridiaceae1 | MD ifo r | Weighted mode | 0.09 | 0.80 (0.64, 1.01) |
| genus Barnesiella | MD ifo r | MR Egger | 0.75 | 1.05 (0.78, 1.42) |
| genus Barnesiella | MD ifo r | Weighted median | 0.11 | 1.11 (0.98, 1.25) |
| genus Barnesiella | MD ifo r | IVW | 0.01 | 1.12 (1.02, 1.22) |
| genus Barnesiella | MD ifo r | Simple mode | 0.43 | 1.09 (0.88, 1.36) |
| genus Barnesiella | MD ifo r | Weighted mode | 0.47 | 1.09 (0.87, 1.37) |
| genus Barnesiella | MD ilf l | MR Egger | 0.51 | 0.90 (0.67, 1.22) |
| genus Barnesiella | MD ilf l | Weighted median | 0.21 | 1.08 (0.96, 1.23) |
| genus Barnesiella | MD ilf l | IVW | 0.04 | 1.10 (1.00, 1.20) |
| genus Barnesiella | MD ilf l | Simple mode | 0.52 | 1.07 (0.88, 1.30) |
| genus Barnesiella | MD ilf l | Weighted mode | 0.55 | 1.07 (0.86, 1.33) |
| genus Alistipes | MD ilf r | MR Egger | 0.67 | 0.87 (0.47, 1.62) |
| genus Alistipes | MD ilf r | Weighted median | 0.18 | 0.90 (0.78, 1.05) |
| genus Alistipes | MD ilf r | IVW | 0.03 | 0.88 (0.77, 0.99) |
| genus Alistipes | MD ilf r | Simple mode | 0.08 | 0.74 (0.53, 1.01) |
| genus Alistipes | MD ilf r | Weighted mode | 0.09 | 0.75 (0.55, 1.02) |
| genus Barnesiella | MD ilf r | MR Egger | 0.71 | 1.06 (0.79, 1.43) |
| genus Barnesiella | MD ilf r | Weighted median | 0.15 | 1.09 (0.97, 1.23) |
| genus Barnesiella | MD ilf r | IVW | 0.02 | 1.11 (1.02, 1.21) |
| genus Barnesiella | MD ilf r | Simple mode | 0.38 | 1.09 (0.90, 1.32) |
| genus Barnesiella | MD ilf r | Weighted mode | 0.54 | 1.06 (0.88, 1.28) |
| family Clostridiaceae1 | MD slf l | MR Egger | 0.36 | 0.85 (0.62, 1.18) |
| family Clostridiaceae1 | MD slf l | Weighted median | 0.02 | 0.84 (0.73, 0.98) |
| family Clostridiaceae1 | MD slf l | IVW | 0.01 | 0.87 (0.78, 0.97) |
| family Clostridiaceae1 | MD slf l | Simple mode | 0.16 | 0.83 (0.65, 1.06) |
| family Clostridiaceae1 | MD slf l | Weighted mode | 0.14 | 0.84 (0.68, 1.04) |
| genus Alistipes | MD slf l | MR Egger | 0.66 | 0.86 (0.45, 1.65) |
| genus Alistipes | MD slf l | Weighted median | 0.29 | 0.92 (0.78, 1.08) |
| genus Alistipes | MD slf l | IVW | 0.02 | 0.86 (0.76, 0.98) |
| genus Alistipes | MD slf l | Simple mode | 0.83 | 0.97 (0.71, 1.31) |
| genus Alistipes | MD slf l | Weighted mode | 0.84 | 0.97 (0.76, 1.25) |
| genus Barnesiella | MD slf l | MR Egger | 0.44 | 0.88 (0.66, 1.19) |
| genus Barnesiella | MD slf l | Weighted median | 0.02 | 1.16 (1.02, 1.32) |
| genus Barnesiella | MD slf l | IVW | 0.02 | 1.11 (1.02, 1.22) |
| genus Barnesiella | MD slf l | Simple mode | 0.21 | 1.16 (0.93, 1.46) |
| genus Barnesiella | MD slf l | Weighted mode | 0.24 | 1.16 (0.91, 1.49) |
| family Clostridiaceae1 | MD slf r | MR Egger | 0.17 | 0.79 (0.58, 1.08) |
| family Clostridiaceae1 | MD slf r | Weighted median | 0.16 | 0.90 (0.78, 1.04) |
| family Clostridiaceae1 | MD slf r | IVW | 0.02 | 0.88 (0.79, 0.98) |
| family Clostridiaceae1 | MD slf r | Simple mode | 0.52 | 0.92 (0.71, 1.18) |
| family Clostridiaceae1 | MD slf r | Weighted mode | 0.18 | 0.84 (0.67, 1.06) |
| genus Alistipes | MD slf r | MR Egger | 0.69 | 0.87 (0.46, 1.67) |
| genus Alistipes | MD slf r | Weighted median | 0.29 | 0.92 (0.79, 1.07) |
| genus Alistipes | MD slf r | IVW | 0.04 | 0.87 (0.77, 0.99) |
| genus Alistipes | MD slf r | Simple mode | 0.88 | 0.97 (0.71, 1.34) |
| genus Alistipes | MD slf r | Weighted mode | 0.87 | 0.98 (0.75, 1.27) |
| genus Alistipes | MD str l | MR Egger | 0.51 | 0.82 (0.47, 1.44) |
| genus Alistipes | MD str l | Weighted median | 0.40 | 0.94 (0.80, 1.09) |
| genus Alistipes | MD str l | IVW | 0.04 | 0.89 (0.79, 0.99) |
| genus Alistipes | MD str l | Simple mode | 0.26 | 0.84 (0.63, 1.12) |
| genus Alistipes | MD str l | Weighted mode | 0.47 | 0.90 (0.69, 1.18) |
| family Clostridiaceae1 | MD str r | MR Egger | 0.23 | 0.82 (0.61, 1.10) |
| family Clostridiaceae1 | MD str r | Weighted median | 0.04 | 0.86 (0.74, 0.99) |
| family Clostridiaceae1 | MD str r | IVW | 0.02 | 0.89 (0.80, 0.98) |
| family Clostridiaceae1 | MD str r | Simple mode | 0.14 | 0.84 (0.68, 1.03) |
| family Clostridiaceae1 | MD str r | Weighted mode | 0.09 | 0.83 (0.68, 1.00) |
| genus Barnesiella | MD unc l | MR Egger | 0.60 | 0.92 (0.67, 1.25) |
| genus Barnesiella | MD unc l | Weighted median | 0.08 | 1.12 (0.99, 1.28) |
| genus Barnesiella | MD unc l | IVW | 1.98 × 10^-3^ | 1.17 (1.06, 1.29) |
| genus Barnesiella | MD unc l | Simple mode | 0.70 | 1.05 (0.82, 1.34) |
| genus Barnesiella | MD unc l | Weighted mode | 0.86 | 1.02 (0.82, 1.26) |
| family Clostridiaceae1 | MD unc r | MR Egger | 0.31 | 0.85 (0.63, 1.14) |
| family Clostridiaceae1 | MD unc r | Weighted median | 0.04 | 0.86 (0.75, 0.99) |
| family Clostridiaceae1 | MD unc r | IVW | 0.05 | 0.90 (0.81, 1.00) |
| family Clostridiaceae1 | MD unc r | Simple mode | 0.21 | 0.86 (0.70, 1.07) |
| family Clostridiaceae1 | MD unc r | Weighted mode | 0.16 | 0.86 (0.71, 1.04) |
| genus Barnesiella | MD unc r | MR Egger | 0.87 | 1.03 (0.72, 1.47) |
| genus Barnesiella | MD unc r | Weighted median | 0.58 | 1.04 (0.91, 1.18) |
| genus Barnesiella | MD unc r | IVW | 0.03 | 1.12 (1.01, 1.24) |
| genus Barnesiella | MD unc r | Simple mode | 0.98 | 1.00 (0.80, 1.24) |
| genus Barnesiella | MD unc r | Weighted mode | 0.98 | 1.00 (0.82, 1.21) |
| genus Alistipes | ICVF ar l | MR Egger | 0.87 | 1.06 (0.55, 2.03) |
| genus Alistipes | ICVF ar l | Weighted median | 0.04 | 1.18 (1.00, 1.39) |
| genus Alistipes | ICVF ar l | IVW | 0.04 | 1.15 (1.01, 1.31) |
| genus Alistipes | ICVF ar l | Simple mode | 0.16 | 1.23 (0.94, 1.62) |
| genus Alistipes | ICVF ar l | Weighted mode | 0.20 | 1.21 (0.92, 1.60) |
| family Clostridiaceae1 | ICVF atr l | MR Egger | 0.44 | 1.15 (0.82, 1.59) |
| family Clostridiaceae1 | ICVF atr l | Weighted median | 0.32 | 1.08 (0.93, 1.26) |
| family Clostridiaceae1 | ICVF atr l | IVW | 0.04 | 1.12 (1.01, 1.25) |
| family Clostridiaceae1 | ICVF atr l | Simple mode | 0.09 | 1.29 (0.99, 1.68) |
| family Clostridiaceae1 | ICVF atr l | Weighted mode | 0.88 | 1.02 (0.82, 1.26) |
| genus Alistipes | ICVF atr l | MR Egger | 0.78 | 1.11 (0.55, 2.23) |
| genus Alistipes | ICVF atr l | Weighted median | 0.03 | 1.20 (1.02, 1.40) |
| genus Alistipes | ICVF atr l | IVW | 0.02 | 1.18 (1.03, 1.36) |
| genus Alistipes | ICVF atr l | Simple mode | 0.23 | 1.24 (0.89, 1.71) |
| genus Alistipes | ICVF atr l | Weighted mode | 0.20 | 1.21 (0.92, 1.60) |
| family Clostridiaceae1 | ICVF atr r | MR Egger | 0.67 | 1.07 (0.78, 1.48) |
| family Clostridiaceae1 | ICVF atr r | Weighted median | 0.24 | 1.09 (0.94, 1.25) |
| family Clostridiaceae1 | ICVF atr r | IVW | 0.04 | 1.12 (1.01, 1.25) |
| family Clostridiaceae1 | ICVF atr r | Simple mode | 0.19 | 1.21 (0.93, 1.56) |
| family Clostridiaceae1 | ICVF atr r | Weighted mode | 0.95 | 0.99 (0.80, 1.23) |
| genus Alistipes | ICVF atr r | MR Egger | 0.82 | 1.09 (0.55, 2.15) |
| genus Alistipes | ICVF atr r | Weighted median | 0.04 | 1.18 (1.01, 1.37) |
| genus Alistipes | ICVF atr r | IVW | 0.01 | 1.20 (1.04, 1.37) |
| genus Alistipes | ICVF atr r | Simple mode | 0.22 | 1.24 (0.89, 1.72) |
| genus Alistipes | ICVF atr r | Weighted mode | 0.32 | 1.16 (0.87, 1.55) |
| family Clostridiaceae1 | ICVF cgc l | MR Egger | 0.22 | 1.23 (0.91, 1.65) |
| family Clostridiaceae1 | ICVF cgc l | Weighted median | 0.08 | 1.14 (0.99, 1.33) |
| family Clostridiaceae1 | ICVF cgc l | IVW | 0.02 | 1.13 (1.02, 1.26) |
| family Clostridiaceae1 | ICVF cgc l | Simple mode | 0.17 | 1.20 (0.94, 1.51) |
| family Clostridiaceae1 | ICVF cgc l | Weighted mode | 0.16 | 1.17 (0.96, 1.43) |
| genus Alistipes | ICVF cgc l | MR Egger | 0.93 | 0.98 (0.58, 1.64) |
| genus Alistipes | ICVF cgc l | Weighted median | 0.01 | 1.24 (1.07, 1.44) |
| genus Alistipes | ICVF cgc l | IVW | 1.88 × 10^-3^ | 1.18 (1.06, 1.32) |
| genus Alistipes | ICVF cgc l | Simple mode | 0.06 | 1.36 (1.01, 1.82) |
| genus Alistipes | ICVF cgc l | Weighted mode | 0.07 | 1.34 (1.00, 1.80) |
| family Clostridiaceae1 | ICVF cgc r | MR Egger | 0.13 | 1.29 (0.96, 1.74) |
| family Clostridiaceae1 | ICVF cgc r | Weighted median | 0.05 | 1.15 (1.00, 1.33) |
| family Clostridiaceae1 | ICVF cgc r | IVW | 0.02 | 1.13 (1.02, 1.26) |
| family Clostridiaceae1 | ICVF cgc r | Simple mode | 0.20 | 1.17 (0.94, 1.46) |
| family Clostridiaceae1 | ICVF cgc r | Weighted mode | 0.16 | 1.17 (0.96, 1.43) |
| genus Alistipes | ICVF cgc r | MR Egger | 0.91 | 1.04 (0.57, 1.88) |
| genus Alistipes | ICVF cgc r | Weighted median | 1.22 × 10^-3^ | 1.30 (1.11, 1.52) |
| genus Alistipes | ICVF cgc r | IVW | 9.92 × 10^-4^ | 1.22 (1.09, 1.38) |
| genus Alistipes | ICVF cgc r | Simple mode | 0.03 | 1.42 (1.07, 1.89) |
| genus Alistipes | ICVF cgc r | Weighted mode | 0.04 | 1.40 (1.05, 1.87) |
| genus Alistipes | ICVF cgh l | MR Egger | 0.45 | 0.79 (0.43, 1.43) |
| genus Alistipes | ICVF cgh l | Weighted median | 0.60 | 1.04 (0.89, 1.22) |
| genus Alistipes | ICVF cgh l | IVW | 0.01 | 1.19 (1.05, 1.35) |
| genus Alistipes | ICVF cgh l | Simple mode | 0.92 | 1.01 (0.80, 1.28) |
| genus Alistipes | ICVF cgh l | Weighted mode | 0.92 | 1.01 (0.81, 1.27) |
| genus Alistipes | ICVF cgh r | MR Egger | 0.36 | 0.78 (0.47, 1.30) |
| genus Alistipes | ICVF cgh r | Weighted median | 0.48 | 1.06 (0.91, 1.23) |
| genus Alistipes | ICVF cgh r | IVW | 0.02 | 1.14 (1.02, 1.27) |
| genus Alistipes | ICVF cgh r | Simple mode | 0.94 | 1.01 (0.78, 1.30) |
| genus Alistipes | ICVF cgh r | Weighted mode | 0.91 | 1.01 (0.79, 1.31) |
| family Clostridiaceae1 | ICVF cst l | MR Egger | 0.22 | 1.23 (0.91, 1.65) |
| family Clostridiaceae1 | ICVF cst l | Weighted median | 0.02 | 1.17 (1.02, 1.34) |
| family Clostridiaceae1 | ICVF cst l | IVW | 0.02 | 1.13 (1.02, 1.26) |
| family Clostridiaceae1 | ICVF cst l | Simple mode | 0.11 | 1.21 (0.98, 1.50) |
| family Clostridiaceae1 | ICVF cst l | Weighted mode | 0.12 | 1.19 (0.98, 1.46) |
| family Clostridiaceae1 | ICVF fma | MR Egger | 0.23 | 1.22 (0.90, 1.64) |
| family Clostridiaceae1 | ICVF fma | Weighted median | 0.02 | 1.19 (1.03, 1.37) |
| family Clostridiaceae1 | ICVF fma | IVW | 2.42 × 10^-3^ | 1.18 (1.06, 1.31) |
| family Clostridiaceae1 | ICVF fma | Simple mode | 0.09 | 1.24 (0.99, 1.54) |
| family Clostridiaceae1 | ICVF fma | Weighted mode | 0.12 | 1.20 (0.98, 1.48) |
| genus Alistipes | ICVF fmi | MR Egger | 0.86 | 1.06 (0.58, 1.93) |
| genus Alistipes | ICVF fmi | Weighted median | 0.39 | 1.07 (0.92, 1.25) |
| genus Alistipes | ICVF fmi | IVW | 0.01 | 1.17 (1.04, 1.32) |
| genus Alistipes | ICVF fmi | Simple mode | 0.71 | 1.05 (0.82, 1.33) |
| genus Alistipes | ICVF fmi | Weighted mode | 0.73 | 1.04 (0.84, 1.30) |
| genus Barnesiella | ICVF fmi | MR Egger | 0.63 | 1.08 (0.80, 1.46) |
| genus Barnesiella | ICVF fmi | Weighted median | 0.22 | 0.92 (0.81, 1.05) |
| genus Barnesiella | ICVF fmi | IVW | 0.04 | 0.91 (0.84, 1.00) |
| genus Barnesiella | ICVF fmi | Simple mode | 0.37 | 0.91 (0.74, 1.12) |
| genus Barnesiella | ICVF fmi | Weighted mode | 0.40 | 0.91 (0.74, 1.12) |
| family Clostridiaceae1 | ICVF ifo l | MR Egger | 0.19 | 1.27 (0.92, 1.77) |
| family Clostridiaceae1 | ICVF ifo l | Weighted median | 0.09 | 1.14 (0.98, 1.33) |
| family Clostridiaceae1 | ICVF ifo l | IVW | 0.03 | 1.14 (1.02, 1.27) |
| family Clostridiaceae1 | ICVF ifo l | Simple mode | 0.12 | 1.25 (0.97, 1.62) |
| family Clostridiaceae1 | ICVF ifo l | Weighted mode | 0.21 | 1.16 (0.93, 1.45) |
| genus Alistipes | ICVF ifo l | MR Egger | 0.60 | 1.19 (0.63, 2.25) |
| genus Alistipes | ICVF ifo l | Weighted median | 0.32 | 1.08 (0.93, 1.27) |
| genus Alistipes | ICVF ifo l | IVW | 0.04 | 1.14 (1.01, 1.30) |
| genus Alistipes | ICVF ifo l | Simple mode | 0.73 | 0.95 (0.71, 1.27) |
| genus Alistipes | ICVF ifo l | Weighted mode | 0.71 | 0.95 (0.71, 1.26) |
| family Clostridiaceae1 | ICVF ifo r | MR Egger | 0.12 | 1.32 (0.97, 1.81) |
| family Clostridiaceae1 | ICVF ifo r | Weighted median | 0.04 | 1.17 (1.00, 1.37) |
| family Clostridiaceae1 | ICVF ifo r | IVW | 0.02 | 1.14 (1.02, 1.27) |
| family Clostridiaceae1 | ICVF ifo r | Simple mode | 0.09 | 1.28 (0.99, 1.64) |
| family Clostridiaceae1 | ICVF ifo r | Weighted mode | 0.10 | 1.22 (0.99, 1.51) |
| genus Alistipes | ICVF ifo r | MR Egger | 0.77 | 1.11 (0.57, 2.17) |
| genus Alistipes | ICVF ifo r | Weighted median | 0.39 | 1.07 (0.92, 1.25) |
| genus Alistipes | ICVF ifo r | IVW | 0.03 | 1.16 (1.02, 1.33) |
| genus Alistipes | ICVF ifo r | Simple mode | 0.93 | 0.99 (0.75, 1.31) |
| genus Alistipes | ICVF ifo r | Weighted mode | 0.83 | 0.97 (0.76, 1.25) |
| family Clostridiaceae1 | ICVF ilf l | MR Egger | 0.23 | 1.25 (0.89, 1.74) |
| family Clostridiaceae1 | ICVF ilf l | Weighted median | 0.10 | 1.14 (0.98, 1.34) |
| family Clostridiaceae1 | ICVF ilf l | IVW | 0.05 | 1.12 (1.00, 1.26) |
| family Clostridiaceae1 | ICVF ilf l | Simple mode | 0.10 | 1.26 (0.98, 1.60) |
| family Clostridiaceae1 | ICVF ilf l | Weighted mode | 0.21 | 1.18 (0.93, 1.49) |
| genus Alistipes | ICVF ilf l | MR Egger | 0.60 | 1.18 (0.64, 2.19) |
| genus Alistipes | ICVF ilf l | Weighted median | 0.25 | 1.10 (0.94, 1.29) |
| genus Alistipes | ICVF ilf l | IVW | 0.02 | 1.16 (1.03, 1.31) |
| genus Alistipes | ICVF ilf l | Simple mode | 0.97 | 0.99 (0.74, 1.33) |
| genus Alistipes | ICVF ilf l | Weighted mode | 0.95 | 0.99 (0.76, 1.29) |
| genus Alistipes | ICVF ilf r | MR Egger | 0.67 | 1.15 (0.62, 2.16) |
| genus Alistipes | ICVF ilf r | Weighted median | 0.26 | 1.09 (0.94, 1.28) |
| genus Alistipes | ICVF ilf r | IVW | 0.01 | 1.18 (1.04, 1.33) |
| genus Alistipes | ICVF ilf r | Simple mode | 0.99 | 1.00 (0.74, 1.35) |
| genus Alistipes | ICVF ilf r | Weighted mode | 0.96 | 0.99 (0.76, 1.30) |
| family Clostridiaceae1 | ICVF ptr l | MR Egger | 0.32 | 1.22 (0.85, 1.76) |
| family Clostridiaceae1 | ICVF ptr l | Weighted median | 0.15 | 1.12 (0.96, 1.31) |
| family Clostridiaceae1 | ICVF ptr l | IVW | 0.04 | 1.14 (1.01, 1.29) |
| family Clostridiaceae1 | ICVF ptr l | Simple mode | 0.46 | 1.10 (0.86, 1.40) |
| family Clostridiaceae1 | ICVF ptr l | Weighted mode | 0.36 | 1.10 (0.90, 1.34) |
| family Clostridiaceae1 | ICVF slf l | MR Egger | 0.15 | 1.28 (0.95, 1.72) |
| family Clostridiaceae1 | ICVF slf l | Weighted median | 0.09 | 1.13 (0.98, 1.31) |
| family Clostridiaceae1 | ICVF slf l | IVW | 0.04 | 1.12 (1.01, 1.24) |
| family Clostridiaceae1 | ICVF slf l | Simple mode | 0.16 | 1.21 (0.95, 1.55) |
| family Clostridiaceae1 | ICVF slf l | Weighted mode | 0.15 | 1.19 (0.96, 1.47) |
| genus Alistipes | ICVF slf l | MR Egger | 0.58 | 1.19 (0.65, 2.21) |
| genus Alistipes | ICVF slf l | Weighted median | 0.17 | 1.11 (0.95, 1.30) |
| genus Alistipes | ICVF slf l | IVW | 0.01 | 1.17 (1.03, 1.32) |
| genus Alistipes | ICVF slf l | Simple mode | 0.38 | 1.14 (0.86, 1.52) |
| genus Alistipes | ICVF slf l | Weighted mode | 0.38 | 1.12 (0.88, 1.44) |
| family Clostridiaceae1 | ICVF slf r | MR Egger | 0.13 | 1.30 (0.96, 1.75) |
| family Clostridiaceae1 | ICVF slf r | Weighted median | 0.14 | 1.11 (0.97, 1.28) |
| family Clostridiaceae1 | ICVF slf r | IVW | 0.02 | 1.13 (1.02, 1.26) |
| family Clostridiaceae1 | ICVF slf r | Simple mode | 0.35 | 1.13 (0.89, 1.43) |
| family Clostridiaceae1 | ICVF slf r | Weighted mode | 0.18 | 1.16 (0.95, 1.42) |
| genus Alistipes | ICVF slf r | MR Egger | 0.65 | 1.15 (0.63, 2.11) |
| genus Alistipes | ICVF slf r | Weighted median | 0.16 | 1.12 (0.96, 1.32) |
| genus Alistipes | ICVF slf r | IVW | 0.01 | 1.17 (1.03, 1.31) |
| genus Alistipes | ICVF slf r | Simple mode | 0.33 | 1.14 (0.88, 1.48) |
| genus Alistipes | ICVF slf r | Weighted mode | 0.42 | 1.12 (0.85, 1.48) |
| family Clostridiaceae1 | ICVF str l | MR Egger | 0.12 | 1.32 (0.96, 1.81) |
| family Clostridiaceae1 | ICVF str l | Weighted median | 0.07 | 1.15 (0.99, 1.34) |
| family Clostridiaceae1 | ICVF str l | IVW | 0.04 | 1.13 (1.01, 1.26) |
| family Clostridiaceae1 | ICVF str l | Simple mode | 0.23 | 1.18 (0.92, 1.53) |
| family Clostridiaceae1 | ICVF str l | Weighted mode | 0.16 | 1.18 (0.95, 1.46) |
| genus Alistipes | ICVF str l | MR Egger | 0.63 | 1.18 (0.61, 2.30) |
| genus Alistipes | ICVF str l | Weighted median | 0.09 | 1.15 (0.98, 1.34) |
| genus Alistipes | ICVF str l | IVW | 0.03 | 1.16 (1.02, 1.32) |
| genus Alistipes | ICVF str l | Simple mode | 0.12 | 1.28 (0.96, 1.71) |
| genus Alistipes | ICVF str l | Weighted mode | 0.21 | 1.20 (0.91, 1.59) |
| family Clostridiaceae1 | ICVF str r | MR Egger | 0.13 | 1.30 (0.96, 1.75) |
| family Clostridiaceae1 | ICVF str r | Weighted median | 0.02 | 1.19 (1.03, 1.38) |
| family Clostridiaceae1 | ICVF str r | IVW | 0.02 | 1.13 (1.02, 1.26) |
| family Clostridiaceae1 | ICVF str r | Simple mode | 0.16 | 1.22 (0.95, 1.56) |
| family Clostridiaceae1 | ICVF str r | Weighted mode | 0.14 | 1.22 (0.96, 1.53) |
| genus Barnesiella | ICVF unc l | MR Egger | 0.49 | 1.12 (0.82, 1.52) |
| genus Barnesiella | ICVF unc l | Weighted median | 0.04 | 0.87 (0.76, 1.00) |
| genus Barnesiella | ICVF unc l | IVW | 0.03 | 0.90 (0.82, 0.99) |
| genus Barnesiella | ICVF unc l | Simple mode | 0.14 | 0.82 (0.64, 1.05) |
| genus Barnesiella | ICVF unc l | Weighted mode | 0.20 | 0.83 (0.63, 1.09) |
| genus Barnesiella | ICVF unc r | MR Egger | 0.89 | 1.03 (0.72, 1.46) |
| genus Barnesiella | ICVF unc r | Weighted median | 0.30 | 0.94 (0.82, 1.06) |
| genus Barnesiella | ICVF unc r | IVW | 0.04 | 0.90 (0.81, 0.99) |
| genus Barnesiella | ICVF unc r | Simple mode | 0.48 | 0.92 (0.72, 1.16) |
| genus Barnesiella | ICVF unc r | Weighted mode | 0.51 | 0.93 (0.74, 1.16) |
| family Clostridiaceae1 | OD cgc r | MR Egger | 0.24 | 0.82 (0.61, 1.11) |
| family Clostridiaceae1 | OD cgc r | Weighted median | 0.10 | 0.88 (0.76, 1.02) |
| family Clostridiaceae1 | OD cgc r | IVW | 4.72 × 10^-3^ | 0.86 (0.77, 0.95) |
| family Clostridiaceae1 | OD cgc r | Simple mode | 0.60 | 0.94 (0.77, 1.16) |
| family Clostridiaceae1 | OD cgc r | Weighted mode | 0.46 | 0.93 (0.76, 1.13) |
| family Clostridiaceae1 | OD ml l | MR Egger | 0.74 | 0.95 (0.70, 1.28) |
| family Clostridiaceae1 | OD ml l | Weighted median | 0.14 | 0.90 (0.79, 1.04) |
| family Clostridiaceae1 | OD ml l | IVW | 0.03 | 0.89 (0.80, 0.99) |
| family Clostridiaceae1 | OD ml l | Simple mode | 0.54 | 0.93 (0.75, 1.16) |
| family Clostridiaceae1 | OD ml l | Weighted mode | 0.69 | 0.96 (0.79, 1.17) |
| family Clostridiaceae1 | OD ptr r | MR Egger | 0.73 | 1.06 (0.78, 1.42) |
| family Clostridiaceae1 | OD ptr r | Weighted median | 0.06 | 1.15 (1.00, 1.33) |
| family Clostridiaceae1 | OD ptr r | IVW | 0.04 | 1.12 (1.01, 1.24) |
| family Clostridiaceae1 | OD ptr r | Simple mode | 0.24 | 1.14 (0.93, 1.40) |
| family Clostridiaceae1 | OD ptr r | Weighted mode | 0.19 | 1.15 (0.95, 1.41) |
| genus Barnesiella | OD str l | MR Egger | 0.65 | 0.93 (0.69, 1.26) |
| genus Barnesiella | OD str l | Weighted median | 0.09 | 0.90 (0.79, 1.02) |
| genus Barnesiella | OD str l | IVW | 0.03 | 0.91 (0.83, 0.99) |
| genus Barnesiella | OD str l | Simple mode | 0.15 | 0.84 (0.67, 1.05) |
| genus Barnesiella | OD str l | Weighted mode | 0.14 | 0.85 (0.69, 1.04) |
| genus Barnesiella | ISOVF cgh r | MR Egger | 0.13 | 0.78 (0.58, 1.05) |
| genus Barnesiella | ISOVF cgh r | Weighted median | 0.06 | 0.89 (0.78, 1.01) |
| genus Barnesiella | ISOVF cgh r | IVW | 0.03 | 0.91 (0.83, 0.99) |
| genus Barnesiella | ISOVF cgh r | Simple mode | 0.27 | 0.87 (0.69, 1.10) |
| genus Barnesiella | ISOVF cgh r | Weighted mode | 0.26 | 0.86 (0.67, 1.11) |
| genus Alistipes | ISOVF fmi | MR Egger | 0.65 | 1.13 (0.68, 1.89) |
| genus Alistipes | ISOVF fmi | Weighted median | 0.01 | 1.23 (1.06, 1.43) |
| genus Alistipes | ISOVF fmi | IVW | 3.75 × 10^-3^ | 1.17 (1.05, 1.30) |
| genus Alistipes | ISOVF fmi | Simple mode | 0.11 | 1.24 (0.97, 1.59) |
| genus Alistipes | ISOVF fmi | Weighted mode | 0.12 | 1.23 (0.97, 1.57) |
| genus Alistipes | ISOVF ifo l | MR Egger | 0.90 | 1.03 (0.62, 1.73) |
| genus Alistipes | ISOVF ifo l | Weighted median | 0.04 | 1.15 (1.01, 1.33) |
| genus Alistipes | ISOVF ifo l | IVW | 0.05 | 1.11 (1.00, 1.24) |
| genus Alistipes | ISOVF ifo l | Simple mode | 0.14 | 1.20 (0.96, 1.52) |
| genus Alistipes | ISOVF ifo l | Weighted mode | 0.17 | 1.20 (0.94, 1.54) |
| genus Alistipes | ISOVF ilf l | MR Egger | 0.87 | 0.96 (0.57, 1.60) |
| genus Alistipes | ISOVF ilf l | Weighted median | 0.03 | 1.17 (1.02, 1.36) |
| genus Alistipes | ISOVF ilf l | IVW | 0.04 | 1.12 (1.01, 1.24) |
| genus Alistipes | ISOVF ilf l | Simple mode | 0.20 | 1.18 (0.93, 1.50) |
| genus Alistipes | ISOVF ilf l | Weighted mode | 0.17 | 1.18 (0.94, 1.48) |
