# Supplementary material for: Gut Microbiota and White Matter Integrity: A Two-Sample Mendelian Randomization Analysis
Source: eNeuro. 2025 Aug 29;12(9):ENEURO.0586-24.2025. doi: 10.1523/ENEURO.0586-24.2025 (PMC12418065; doi:10.1523/ENEURO.0586-24.2025)
Supplement: Figure 3-2 — Tests for heterogeneity and pleiotropy in the causal effect of GM on white matter microstructure. Download Figure 3-2, DOC file. [file eneuro-12-ENEURO.0586-24.2025-s006.doc]

Figure 3-2

Tests for heterogeneity and pleiotropy in the causal effect of GM on white matter microstructure

| Mendelian randomization | | Sensitivity analysis | |
| --- | --- | --- | --- |
| Exposure | Outcome | *p* (Heterogeneity test) | *p* (Pleiotropy test) |
| genus Alistipes | FA ar l | 0.67 | 0.94 |
| genus Alistipes | FA atr l | 0.12 | 0.45 |
| genus Alistipes | FA atr r | 0.10 | 0.46 |
| genus Alistipes | FA cgc l | 0.65 | 0.93 |
| family Clostridiaceae1 | FA cgc r | 0.63 | 0.36 |
| genus Alistipes | FA cgc r | 0.17 | 0.63 |
| genus Alistipes | FA fmi | 0.38 | 0.73 |
| genus Barnesiella | FA fmi | 0.79 | 0.08 |
| family Clostridiaceae1 | FA ifo l | 0.62 | 0.41 |
| genus Alistipes | FA ifo l | 0.47 | 0.75 |
| genus Barnesiella | FA ifo l | 0.36 | 0.15 |
| family Clostridiaceae1 | FA ifo r | 0.51 | 0.22 |
| genus Barnesiella | FA ifo r | 0.60 | 0.14 |
| family Clostridiaceae1 | FA ilf l | 0.63 | 0.43 |
| genus Alistipes | FA ilf l | 0.51 | 0.99 |
| genus Barnesiella | FA ilf l | 0.79 | 0.17 |
| genus Alistipes | FA ilf r | 0.22 | 0.57 |
| genus Alistipes | FA ptr l | 0.47 | 0.58 |
| genus Alistipes | FA ptr r | 0.47 | 0.50 |
| family Clostridiaceae1 | FA slf l | 0.53 | 0.40 |
| genus Alistipes | FA slf l | 0.20 | 0.49 |
| family Clostridiaceae1 | FA slf r | 0.19 | 0.36 |
| genus Alistipes | FA slf r | 0.30 | 0.77 |
| family Clostridiaceae1 | FA str l | 0.28 | 0.83 |
| family Clostridiaceae1 | FA str r | 0.36 | 0.85 |
| genus Alistipes | FA unc l | 0.19 | 0.86 |
| family Clostridiaceae1 | MD ar l | 0.92 | 0.82 |
| genus Alistipes | MD ar l | 0.44 | 0.81 |
| genus Alistipes | MD atr l | 0.30 | 0.89 |
| genus Barnesiella | MD atr l | 0.27 | 0.09 |
| genus Alistipes | MD atr r | 0.13 | 0.98 |
| genus Barnesiella | MD atr r | 0.50 | 0.21 |
| genus Alistipes | MD cgc l | 0.39 | 0.90 |
| genus Alistipes | MD cgc r | 0.72 | 0.62 |
| family Clostridiaceae1 | MD cst l | 0.29 | 0.87 |
| genus Alistipes | MD cst l | 0.30 | 0.65 |
| family Clostridiaceae1 | MD cst r | 0.87 | 0.62 |
| genus Barnesiella | MD cst r | 0.51 | 0.66 |
| family Clostridiaceae1 | MD ifo l | 0.23 | 0.58 |
| genus Barnesiella | MD ifo l | 0.34 | 0.07 |
| family Clostridiaceae1 | MD ifo r | 0.44 | 0.91 |
| genus Barnesiella | MD ifo r | 0.59 | 0.68 |
| genus Barnesiella | MD ilf l | 0.66 | 0.20 |
| genus Alistipes | MD ilf r | 0.18 | 0.99 |
| genus Barnesiella | MD ilf r | 0.69 | 0.75 |
| family Clostridiaceae1 | MD slf l | 0.40 | 0.89 |
| genus Alistipes | MD slf l | 0.13 | 1.00 |
| genus Barnesiella | MD slf l | 0.50 | 0.14 |
| family Clostridiaceae1 | MD slf r | 0.40 | 0.47 |
| genus Alistipes | MD slf r | 0.12 | 1.00 |
| genus Alistipes | MD str l | 0.35 | 0.79 |
| family Clostridiaceae1 | MD str r | 0.54 | 0.60 |
| genus Barnesiella | MD unc l | 0.25 | 0.14 |
| family Clostridiaceae1 | MD unc r | 0.58 | 0.69 |
| genus Barnesiella | MD unc r | 0.20 | 0.64 |
| genus Alistipes | ICVF ar l | 0.11 | 0.81 |
| family Clostridiaceae1 | ICVF atr l | 0.36 | 0.90 |
| genus Alistipes | ICVF atr l | 0.05 | 0.86 |
| family Clostridiaceae1 | ICVF atr r | 0.41 | 0.79 |
| genus Alistipes | ICVF atr r | 0.07 | 0.78 |
| family Clostridiaceae1 | ICVF cgc l | 0.62 | 0.58 |
| genus Alistipes | ICVF cgc l | 0.46 | 0.47 |
| family Clostridiaceae1 | ICVF cgc r | 0.88 | 0.38 |
| genus Alistipes | ICVF cgc r | 0.22 | 0.59 |
| genus Alistipes | ICVF cgh l | 0.12 | 0.19 |
| genus Alistipes | ICVF cgh r | 0.38 | 0.16 |
| family Clostridiaceae1 | ICVF cst l | 0.85 | 0.60 |
| family Clostridiaceae1 | ICVF fma | 0.86 | 0.82 |
| genus Alistipes | ICVF fmi | 0.21 | 0.73 |
| genus Barnesiella | ICVF fmi | 0.50 | 0.27 |
| family Clostridiaceae1 | ICVF ifo l | 0.32 | 0.50 |
| genus Alistipes | ICVF ifo l | 0.15 | 0.90 |
| family Clostridiaceae1 | ICVF ifo r | 0.36 | 0.34 |
| genus Alistipes | ICVF ifo r | 0.08 | 0.89 |
| family Clostridiaceae1 | ICVF ilf l | 0.32 | 0.53 |
| genus Alistipes | ICVF ilf l | 0.19 | 0.95 |
| genus Alistipes | ICVF ilf r | 0.16 | 0.95 |
| family Clostridiaceae1 | ICVF ptr l | 0.20 | 0.71 |
| family Clostridiaceae1 | ICVF slf l | 0.51 | 0.38 |
| genus Alistipes | ICVF slf l | 0.19 | 0.94 |
| family Clostridiaceae1 | ICVF slf r | 0.68 | 0.38 |
| genus Alistipes | ICVF slf r | 0.21 | 0.97 |
| family Clostridiaceae1 | ICVF str l | 0.34 | 0.33 |
| genus Alistipes | ICVF str l | 0.09 | 0.96 |
| family Clostridiaceae1 | ICVF str r | 0.69 | 0.37 |
| genus Barnesiella | ICVF unc l | 0.33 | 0.17 |
| genus Barnesiella | ICVF unc r | 0.18 | 0.45 |
| family Clostridiaceae1 | OD cgc r | 0.91 | 0.77 |
| family Clostridiaceae1 | OD ml l | 0.95 | 0.67 |
| family Clostridiaceae1 | OD ptr r | 0.68 | 0.69 |
| genus Barnesiella | OD str l | 0.52 | 0.86 |
| genus Barnesiella | ISOVF cgh r | 0.51 | 0.32 |
| genus Alistipes | ISOVF fmi | 0.63 | 0.90 |
| genus Alistipes | ISOVF ifo l | 0.95 | 0.78 |
| genus Alistipes | ISOVF ilf l | 0.50 | 0.56 |
