# Supplementary material for: Gut Microbiota and White Matter Integrity: A Two-Sample Mendelian Randomization Analysis
Source: eNeuro. 2025 Aug 29;12(9):ENEURO.0586-24.2025. doi: 10.1523/ENEURO.0586-24.2025 (PMC12418065; doi:10.1523/ENEURO.0586-24.2025)
Supplement: Figure 4-1 — Tests for heterogeneity and pleiotropy in the causal effect of GM on white matter connectivity. Download Figure 4-1, DOC file. [file eneuro-12-ENEURO.0586-24.2025-s007.doc]

Figure 4-1

Tests for heterogeneity and pleiotropy in the causal effect of GM on white matter connectivity

| Mendelian randomization | | Sensitivity analysis | |
| --- | --- | --- | --- |
| Exposure | Outcome | *p* (Heterogeneity test) | *p* (Pleiotropy test) |
| family Rhodospirillaceae | GCST90302666 | 0.69 | 0.34 |
| order Rhodospirillales | GCST90302666 | 0.47 | 0.62 |
| order Desulfovibrionales | GCST90302676 | 0.51 | 0.51 |
| order Desulfovibrionales | GCST90302677 | 0.83 | 0.82 |
| genus Veillonella | GCST90302696 | 0.95 | 0.77 |
| genus Escherichia Shigella | GCST90302698 | 0.87 | 0.47 |
| family Desulfovibrionaceae | GCST90302717 | 0.65 | 0.42 |
| order Desulfovibrionales | GCST90302717 | 0.72 | 0.28 |
| genus Howardella | GCST90302727 | 0.56 | 0.54 |
| genus Ruminococcus gnavus group | GCST90302735 | 0.63 | 0.24 |
| genus Senegalimassilia | GCST90302795 | 0.39 | 0.43 |
| genus Tyzzerella3 | GCST90302666 | 0.88 | 0.47 |
