# Supplementary material for: Gut Microbiota and White Matter Integrity: A Two-Sample Mendelian Randomization Analysis
Source: eNeuro. 2025 Aug 29;12(9):ENEURO.0586-24.2025. doi: 10.1523/ENEURO.0586-24.2025 (PMC12418065; doi:10.1523/ENEURO.0586-24.2025)
Supplement: Figure 5-1 — Mendelian randomization estimates the causal effect of 17 selected bacterial taxa and 13 neurological diseases. Download Figure 5-1, DOC file. [file eneuro-12-ENEURO.0586-24.2025-s008.doc]

Figure 5-1

Mendelian randomization estimates the causal effect of 17 selected bacterial taxa and 13 neurological diseases

| Exposure | Outcome | Method | *p* | OR (95% CI) |
| --- | --- | --- | --- | --- |
| genus Ruminococcus gnavus group | Amyotrophic lateral sclerosis | MR Egger | 0.77 | 0.92 (0.54, 1.56) |
| genus Ruminococcus gnavus group | Amyotrophic lateral sclerosis | Weighted median | 0.08 | 0.89 (0.78, 1.01) |
| genus Ruminococcus gnavus group | Amyotrophic lateral sclerosis | IVW | 0.03 | 0.88 (0.79, 0.99) |
| genus Ruminococcus gnavus group | Amyotrophic lateral sclerosis | Simple mode | 0.08 | 0.78 (0.60, 1.01) |
| genus Ruminococcus gnavus group | Amyotrophic lateral sclerosis | Weighted mode | 0.12 | 0.81 (0.63, 1.04) |
| family Desulfovibrionaceae | Cardioembolism | MR Egger | 0.30 | 0.71 (0.39, 1.30) |
| family Desulfovibrionaceae | Cardioembolism | Weighted median | 0.13 | 0.80 (0.59, 1.07) |
| family Desulfovibrionaceae | Cardioembolism | IVW | 0.01 | 0.76 (0.61, 0.95) |
| family Desulfovibrionaceae | Cardioembolism | Simple mode | 0.23 | 0.74 (0.46, 1.17) |
| family Desulfovibrionaceae | Cardioembolism | Weighted mode | 0.41 | 0.84 (0.56, 1.25) |
| order Desulfovibrionales | Cardioembolism | MR Egger | 0.19 | 0.67 (0.38, 1.17) |
| order Desulfovibrionales | Cardioembolism | Weighted median | 0.32 | 0.87 (0.66, 1.14) |
| order Desulfovibrionales | Cardioembolism | IVW | 0.03 | 0.80 (0.65, 0.98) |
| order Desulfovibrionales | Cardioembolism | Simple mode | 0.52 | 0.86 (0.54, 1.36) |
| order Desulfovibrionales | Cardioembolism | Weighted mode | 0.57 | 0.89 (0.60, 1.32) |
| genus Barnesiella | Ischemic stroke | MR Egger | 0.80 | 0.95 (0.66, 1.38) |
| genus Barnesiella | Ischemic stroke | Weighted median | 0.31 | 0.93 (0.80, 1.07) |
| genus Barnesiella | Ischemic stroke | IVW | 0.05 | 0.90 (0.81, 1.00) |
| genus Barnesiella | Ischemic stroke | Simple mode | 0.43 | 0.91 (0.73, 1.14) |
| genus Barnesiella | Ischemic stroke | Weighted mode | 0.43 | 0.91 (0.74, 1.14) |
| family Clostridiaceae1 | Ischemic stroke | MR Egger | 0.09 | 0.71 (0.50, 1.00) |
| family Clostridiaceae1 | Ischemic stroke | Weighted median | 0.01 | 0.82 (0.71, 0.95) |
| family Clostridiaceae1 | Ischemic stroke | IVW | 2.03 × 10^-3^ | 0.84 (0.75, 0.94) |
| family Clostridiaceae1 | Ischemic stroke | Simple mode | 0.13 | 0.83 (0.67, 1.04) |
| family Clostridiaceae1 | Ischemic stroke | Weighted mode | 0.11 | 0.83 (0.68, 1.02) |
| genus Barnesiella | Small vessel stroke | MR Egger | 0.33 | 0.67 (0.31, 1.45) |
| genus Barnesiella | Small vessel stroke | Weighted median | 0.05 | 0.72 (0.52, 1.00) |
| genus Barnesiella | Small vessel stroke | IVW | 4.59 × 10^-3^ | 0.72 (0.58, 0.90) |
| genus Barnesiella | Small vessel stroke | Simple mode | 0.39 | 0.79 (0.46, 1.33) |
| genus Barnesiella | Small vessel stroke | Weighted mode | 0.30 | 0.75 (0.45, 1.26) |
| genus Barnesiella | AQP4-IgG + NMOSD | MR Egger | 0.70 | 2.31 (0.03, 157.85) |
| genus Barnesiella | AQP4-IgG + NMOSD | Weighted median | 0.05 | 5.04 (1.00, 25.44) |
| genus Barnesiella | AQP4-IgG + NMOSD | IVW | 0.02 | 4.64 (1.34, 16.11) |
| genus Barnesiella | AQP4-IgG + NMOSD | Simple mode | 0.26 | 5.75 (0.31, 107.89) |
| genus Barnesiella | AQP4-IgG + NMOSD | Weighted mode | 0.29 | 4.59 (0.30, 69.78) |
| genus Barnesiella | NMOSD | MR Egger | 0.65 | 2.21 (0.08, 62.35) |
| genus Barnesiella | NMOSD | Weighted median | 0.15 | 2.72 (0.69, 10.68) |
| genus Barnesiella | NMOSD | IVW | 0.03 | 2.95 (1.09, 7.98) |
| genus Barnesiella | NMOSD | Simple mode | 0.36 | 3.14 (0.29, 34.11) |
| genus Barnesiella | NMOSD | Weighted mode | 0.73 | 1.44 (0.19, 10.76) |
