# Supplementary material for: Gut Microbiota and White Matter Integrity: A Two-Sample Mendelian Randomization Analysis
Source: eNeuro. 2025 Aug 29;12(9):ENEURO.0586-24.2025. doi: 10.1523/ENEURO.0586-24.2025 (PMC12418065; doi:10.1523/ENEURO.0586-24.2025)
Supplement: Figure 5-2 — Tests for heterogeneity and pleiotropy in the causal effect of 17 selected bacterial taxa on 13 neurological diseases. Download Figure 5-2, DOC file. [file eneuro-12-ENEURO.0586-24.2025-s009.doc]

Figure 5-2

Tests for heterogeneity and pleiotropy in the causal effect of 17 selected bacterial taxa on 13 neurological diseases

| Mendelian randomization | | Sensitivity analysis | |
| --- | --- | --- | --- |
| Exposure | Outcome | *p* (Heterogeneity test) | *p* (Pleiotropy test) |
| genus Ruminococcus gnavus group | Amyotrophic lateral sclerosis | 0.19 | 0.88 |
| family Desulfovibrionaceae | Cardioembolism | 0.37 | 0.82 |
| order Desulfovibrionales | Cardioembolism | 0.39 | 0.52 |
| genus Barnesiella | Ischemic stroke | 0.29 | 0.75 |
| family Clostridiaceae | Ischemic stroke | 0.83 | 0.34 |
| genus Barnesiella | Small vessel stroke | 0.65 | 0.85 |
| genus Barnesiella | AQP4-IgG + NMOSD | 0.96 | 0.74 |
| genus Barnesiella | NMOSD | 0.89 | 0.86 |
