# Supplementary material for: Gut Microbiota and White Matter Integrity: A Two-Sample Mendelian Randomization Analysis
Source: eNeuro. 2025 Aug 29;12(9):ENEURO.0586-24.2025. doi: 10.1523/ENEURO.0586-24.2025 (PMC12418065; doi:10.1523/ENEURO.0586-24.2025)
Supplement: Figure 6-1 — The genes mapped using the FUMA tool for 10 bacterial taxa. Download Figure 6-1, DOC file. [file eneuro-12-ENEURO.0586-24.2025-s010.doc]

Figure 6-1

The genes mapped using the FUMA tool for 10 bacterial taxa

| Gut microbiota | Mapped genes |
| --- | --- |
| order Desulfovibrionales | *FMOD, LAX1, NEU2, INPP5D, STT3B, IMPAD1, TTC39B, ECHDC3, PROSER2, CMTR2, ZNF23, ZNF19, CHST4, AP1G1, HP, RAP1GAP2, EIF2S2, ITCH, DYNLRB1, MAP1LC3A, PIGU, NCOA6, GGT7, ACSS2, GSS, MYH7B, TRPC4AP, EDEM2, PROCR, MMP24, EIF6, UQCC1, CPNE1, SIK1, HSF2BP, RRP1B, PDXK, CSTB* |
| family Desulfovibrionaceae | *FMOD, LAX1, NEU2, INPP5D, IMPAD1, TTC39B, ECHDC3, PROSER2, CMTR2, ZNF23, ZNF19, CHST4, AP1G1, HP, RAP1GAP2, EIF2S2, ITCH, DYNLRB1,*  *MAP1LC3A, PIGU, NCOA6, GGT7, ACSS2, GSS, MYH7B, TRPC4AP, EDEM2, PROCR, MMP24, EIF6, UQCC1, CPNE1, SIK1, HSF2BP, RRP1B, PDXK, CSTB* |
| order Rhodospirillales | *STK39, FAM124B, DOCK10, PIK3R1, PRR16, ADCY8, ZFPM1* |
| family Rhodospirillaceae | *AK5, ZZZ3, GIPC2, PTGFR, CDC7, TGFBR3, STK39, FAM124B, DOCK10, PRR16,*  *CNR1, RNGTT, PNRC1, RRAGD, ADCY8, ZFPM1* |
| genus Escherichia Shigella | *SPATA6, AGAP1, IL5RA, TRNT1, CRBN, FAM151B, ANKRD34B, DHFR, MSH3, ADCYAP1R1, IMPDH1, HILPDA, CALU, OPN1SW, BRAF, RALGDS, SURF6, MED22, SURF1, SURF2, ADAMTS13, CACFD1, SLC2A6, CTNNA3, SIRT1, NAA60, C16orf90, CLUAP1, SLX4, DNASE1, TRAP1, TTC3, DSCR4, KCNJ15* |
| genus Howardella | *NEGR1, TNS1, ANXA10, DDX60, DDX60L, GNGT1, TFPI2, EXOC4, LRGUK, SLC35B4, AKR1B1, INTS4, NDUFC2, ALG8, KCTD21, USP35, NARS2, ARRDC4, PLD6, FLCN, COPS3, NT5M, SLC14A2, SLC14A1, PIGN, ZCCHC2* |
| genus Ruminococcus gnavus group | *TRIM33, BCAS2, DENND2C, AMPD1, TFCP2L1, CLASP1, NIFK, TSN, GTDC1, PLSCR1, PLSCR5, AK8, C9orf9, GFI1B, RALGDS, PRKCQ, TMEM8A, NME4, CAPN15* |
| genus Senegalimassilia | *NRP2, FBN2, SLC27A6, DCAF12, UBAP1,*  *KIF24, NUDT2, KIAA1161, GALT, IL11RA,*  *EGLN3, ST6GALNAC1, MGAT5B* |
| genus Tyzzerella3 | *VRK2, FANCL, FAM134B, FARS2, PCLO,*  *B4GALT1, OR13A1, ALOX5, MARCH8, ZFAND4, FAM21C, FAM25E, AGAP4, FOXN3, C17orf85, CFD, WDR18, GRIN3B, TMEM259, CNN2, ABCA7, POLR2E, GPX4* |
| genus Veillonella | *FAM20B, TOR3A, SOAT1, SNX4, ALG1L, SLC41A3, PRR16, DLC1, SLC22A18AS, SLC22A18, PHLDA2, NAP1L4, CARS, ACCS, EXT2, ALX4, TMEM132D, N6AMT1, LTN1, RWDD2B, CCT8, MAP3K7CL, GRIK1, NUP50, KIAA0930* |

Genes in red font are not found in eqtlgen
