# Supplementary material for: Gut Microbiota and White Matter Integrity: A Two-Sample Mendelian Randomization Analysis
Source: eNeuro. 2025 Aug 29;12(9):ENEURO.0586-24.2025. doi: 10.1523/ENEURO.0586-24.2025 (PMC12418065; doi:10.1523/ENEURO.0586-24.2025)
Supplement: Figure 6-2 — MR analysis of bacterial taxa-mapped genes and their association with white matter connectivity. Download Figure 6-2, DOC file. [file eneuro-12-ENEURO.0586-24.2025-s011.doc]

Figure 6-2

MR analysis of bacterial taxa-mapped genes and their association with white matter connectivity

| Gut microbiota | Exposure | Outcome | Method | *p* | OR (95% CI) |
| --- | --- | --- | --- | --- | --- |
| order Rhodospirillales | *DOCK10* | GCST90302666 | MR Egger | 0.01 | 1.05 (1.02, 1.09) |
| order Rhodospirillales | *DOCK10* | GCST90302666 | Weighted median | 4.76 × 10^-3^ | 1.04 (1.01, 1.07) |
| order Rhodospirillales | *DOCK10* | GCST90302666 | Inverse variance weighted | 6.19 ×10^-5^ | 1.04 (1.02, 1.06) |
| order Rhodospirillales | *DOCK10* | GCST90302666 | Simple mode | 0.32 | 1.02 (0.98, 1.07) |
| order Rhodospirillales | *DOCK10* | GCST90302666 | Weighted mode | 0.02 | 1.04 (1.01, 1.07) |
| family Rhodospirillaceae | *CNR1* | GCST90302666 | MR Egger | 0.29 | 0.98 (0.93, 1.02) |
| family Rhodospirillaceae | *CNR1* | GCST90302666 | Weighted median | 0.03 | 0.97 (0.94, 1.00) |
| family Rhodospirillaceae | *CNR1* | GCST90302666 | Inverse variance weighted | 3.69 × 10^-3^ | 0.97 (0.95, 0.99) |
| family Rhodospirillaceae | *CNR1* | GCST90302666 | Simple mode | 0.68 | 0.99 (0.94, 1.04) |
| family Rhodospirillaceae | *CNR1* | GCST90302666 | Weighted mode | 0.08 | 0.97 (0.94, 1.00) |
| family Rhodospirillaceae | *DOCK10* | GCST90302666 | MR Egger | 0.01 | 1.05 (1.02, 1.09) |
| family Rhodospirillaceae | *DOCK10* | GCST90302666 | Weighted median | 4.03 × 10^-3^ | 1.04 (1.01, 1.07) |
| family Rhodospirillaceae | *DOCK10* | GCST90302666 | Inverse variance weighted | 6.19 ×10^-5^ | 1.04 (1.02, 1.06) |
| family Rhodospirillaceae | *DOCK10* | GCST90302666 | Simple mode | 0.32 | 1.02 (0.98, 1.07) |
| family Rhodospirillaceae | *DOCK10* | GCST90302666 | Weighted mode | 0.03 | 1.04 (1.01, 1.07) |
| family Rhodospirillaceae | *RRAGD* | GCST90302666 | MR Egger | 0.01 | 0.97 (0.95, 0.99) |
| family Rhodospirillaceae | *RRAGD* | GCST90302666 | Weighted median | 0.08 | 0.98 (0.96, 1.00) |
| family Rhodospirillaceae | *RRAGD* | GCST90302666 | Inverse variance weighted | 3.39 × 10^-3^ | 0.98 (0.97, 0.99) |
| family Rhodospirillaceae | *RRAGD* | GCST90302666 | Simple mode | 0.69 | 0.99 (0.96, 1.03) |
| family Rhodospirillaceae | *RRAGD* | GCST90302666 | Weighted mode | 0.08 | 0.98 (0.97, 1.00) |
| family Rhodospirillaceae | *ZZZ3* | GCST90302666 | MR Egger | 0.74 | 1.02 (0.90, 1.17) |
| family Rhodospirillaceae | *ZZZ3* | GCST90302666 | Weighted median | 0.13 | 1.05 (0.99, 1.12) |
| family Rhodospirillaceae | *ZZZ3* | GCST90302666 | Inverse variance weighted | 2.17 × 10^-3^ | 1.08 (1.03, 1.13) |
| family Rhodospirillaceae | *ZZZ3* | GCST90302666 | Simple mode | 0.14 | 1.09 (0.98, 1.21) |
| family Rhodospirillaceae | *ZZZ3* | GCST90302666 | Weighted mode | 0.21 | 1.06 (0.98, 1.14) |
| genus Escherichia Shigella | *ADAMTS13* | GCST90302698 | MR Egger | 0.78 | 1.01 (0.96, 1.05) |
| genus Escherichia Shigella | *ADAMTS13* | GCST90302698 | Weighted median | 0.21 | 1.02 (0.99, 1.06) |
| genus Escherichia Shigella | *ADAMTS13* | GCST90302698 | Inverse variance weighted | 0.04 | 1.03 (1.00, 1.07) |
| genus Escherichia Shigella | *ADAMTS13* | GCST90302698 | Simple mode | 0.59 | 1.02 (0.96, 1.07) |
| genus Escherichia Shigella | *ADAMTS13* | GCST90302698 | Weighted mode | 0.39 | 1.02 (0.98, 1.06) |
| genus Escherichia Shigella | *ANKRD34B* | GCST90302698 | MR Egger | 0.72 | 1.00 (0.99, 1.02) |
| genus Escherichia Shigella | *ANKRD34B* | GCST90302698 | Weighted median | 0.23 | 1.01 (0.99, 1.02) |
| genus Escherichia Shigella | *ANKRD34B* | GCST90302698 | Inverse variance weighted | 0.01 | 1.01 (1.00, 1.02) |
| genus Escherichia Shigella | *ANKRD34B* | GCST90302698 | Simple mode | 0.99 | 1.00 (0.97, 1.03) |
| genus Escherichia Shigella | *ANKRD34B* | GCST90302698 | Weighted mode | 0.28 | 1.01 (0.99, 1.02) |
| genus Escherichia Shigella | *CACFD1* | GCST90302698 | MR Egger | 0.35 | 1.04 (0.96, 1.13) |
| genus Escherichia Shigella | *CACFD1* | GCST90302698 | Weighted median | 0.03 | 1.05 (1.01, 1.11) |
| genus Escherichia Shigella | *CACFD1* | GCST90302698 | Inverse variance weighted | 0.03 | 1.04 (1.00, 1.09) |
| genus Escherichia Shigella | *CACFD1* | GCST90302698 | Simple mode | 0.24 | 1.04 (0.98, 1.11) |
| genus Escherichia Shigella | *CACFD1* | GCST90302698 | Weighted mode | 0.06 | 1.06 (1.00, 1.12) |
| genus Escherichia Shigella | *DHFR* | GCST90302698 | MR Egger | 0.38 | 1.01 (0.99, 1.02) |
| genus Escherichia Shigella | *DHFR* | GCST90302698 | Weighted median | 0.62 | 1.00 (0.99, 1.01) |
| genus Escherichia Shigella | *DHFR* | GCST90302698 | Inverse variance weighted | 0.04 | 1.01 (1.00, 1.01) |
| genus Escherichia Shigella | *DHFR* | GCST90302698 | Simple mode | 0.60 | 1.01 (0.99, 1.02) |
| genus Escherichia Shigella | *DHFR* | GCST90302698 | Weighted mode | 0.49 | 1.00 (0.99, 1.01) |
| genus Escherichia Shigella | *FAM151B* | GCST90302698 | MR Egger | 0.17 | 1.05 (0.98, 1.12) |
| genus Escherichia Shigella | *FAM151B* | GCST90302698 | Weighted median | 0.04 | 1.04 (1.00, 1.08) |
| genus Escherichia Shigella | *FAM151B* | GCST90302698 | Inverse variance weighted | 1.71 × 10^-3^ | 1.05 (1.02, 1.08) |
| genus Escherichia Shigella | *FAM151B* | GCST90302698 | Simple mode | 0.32 | 1.04 (0.96, 1.12) |
| genus Escherichia Shigella | *FAM151B* | GCST90302698 | Weighted mode | 0.21 | 1.03 (0.98, 1.09) |
| genus Escherichia Shigella | *MED22* | GCST90302698 | MR Egger | 0.07 | 0.96 (0.92, 1.00) |
| genus Escherichia Shigella | *MED22* | GCST90302698 | Weighted median | 1.15 × 10^-3^ | 0.96 (0.94, 0.98) |
| genus Escherichia Shigella | *MED22* | GCST90302698 | Inverse variance weighted | 4.77 × 10^-5^ | 0.96 (0.95, 0.98) |
| genus Escherichia Shigella | *MED22* | GCST90302698 | Simple mode | 0.21 | 0.97 (0.93, 1.01) |
| genus Escherichia Shigella | *MED22* | GCST90302698 | Weighted mode | 0.01 | 0.96 (0.93, 0.99) |
| genus Escherichia Shigella | *MSH3* | GCST90302698 | MR Egger | 0.04 | 0.99 (0.97, 1.00) |
| genus Escherichia Shigella | *MSH3* | GCST90302698 | Weighted median | 0.02 | 0.99 (0.98, 1.00) |
| genus Escherichia Shigella | *MSH3* | GCST90302698 | Inverse variance weighted | 0.03 | 0.99 (0.98, 1.00) |
| genus Escherichia Shigella | *MSH3* | GCST90302698 | Simple mode | 0.82 | 1.00 (0.98, 1.02) |
| genus Escherichia Shigella | *MSH3* | GCST90302698 | Weighted mode | 3.73 × 10^-4^ | 0.99 (0.98, 1.00) |
| genus Escherichia Shigella | *NAA60* | GCST90302698 | MR Egger | 0.29 | 0.98 (0.94, 1.02) |
| genus Escherichia Shigella | *NAA60* | GCST90302698 | Weighted median | 0.03 | 0.97 (0.95, 1.00) |
| genus Escherichia Shigella | *NAA60* | GCST90302698 | Inverse variance weighted | 0.04 | 0.98 (0.96, 1.00) |
| genus Escherichia Shigella | *NAA60* | GCST90302698 | Simple mode | 0.39 | 0.98 (0.94, 1.03) |
| genus Escherichia Shigella | *NAA60* | GCST90302698 | Weighted mode | 0.05 | 0.97 (0.95, 1.00) |
| genus Escherichia Shigella | *SURF6* | GCST90302698 | MR Egger | 0.39 | 0.98 (0.95, 1.02) |
| genus Escherichia Shigella | *SURF6* | GCST90302698 | Weighted median | 3.73 × 10^-4^ | 0.96 (0.94, 0.98) |
| genus Escherichia Shigella | *SURF6* | GCST90302698 | Inverse variance weighted | 1.05 × 10^-5^ | 0.96 (0.95, 0.98) |
| genus Escherichia Shigella | *SURF6* | GCST90302698 | Simple mode | 0.03 | 0.96 (0.93, 0.99) |
| genus Escherichia Shigella | *SURF6* | GCST90302698 | Weighted mode | 2.47 × 10^-3^ | 0.96 (0.94, 0.98) |
| genus Escherichia Shigella | *TRNT1* | GCST90302698 | MR Egger | 0.50 | 0.99 (0.96, 1.02) |
| genus Escherichia Shigella | *TRNT1* | GCST90302698 | Weighted median | 0.43 | 0.99 (0.97, 1.01) |
| genus Escherichia Shigella | *TRNT1* | GCST90302698 | Inverse variance weighted | 0.03 | 0.98 (0.96, 1.00) |
| genus Escherichia Shigella | *TRNT1* | GCST90302698 | Simple mode | 0.18 | 0.97 (0.93, 1.01) |
| genus Escherichia Shigella | *TRNT1* | GCST90302698 | Weighted mode | 0.49 | 0.99 (0.97, 1.02) |
| genus Howardella id.2000 | *COPS3* | GCST90302727 | MR Egger | 0.01 | 1.03 (1.01, 1.05) |
| genus Howardella id.2000 | *COPS3* | GCST90302727 | Weighted median | 5.94 ×10^-4^ | 1.03 (1.01, 1.05) |
| genus Howardella id.2000 | *COPS3* | GCST90302727 | Inverse variance weighted | 4.37 ×10^-7^ | 1.03 (1.02, 1.04) |
| genus Howardella id.2000 | *COPS3* | GCST90302727 | Simple mode | 0.12 | 1.03 (0.99, 1.06) |
| genus Howardella id.2000 | *COPS3* | GCST90302727 | Weighted mode | 0.01 | 1.03 (1.01, 1.05) |
| genus Howardella id.2000 | *PLD6* | GCST90302727 | MR Egger | 0.84 | 1.00 (0.98, 1.02) |
| genus Howardella id.2000 | *PLD6* | GCST90302727 | Weighted median | 0.01 | 0.98 (0.96, 0.99) |
| genus Howardella id.2000 | *PLD6* | GCST90302727 | Inverse variance weighted | 0.01 | 0.99 (0.97, 1.00) |
| genus Howardella id.2000 | *PLD6* | GCST90302727 | Simple mode | 0.85 | 1.00 (0.97, 1.02) |
| genus Howardella id.2000 | *PLD6* | GCST90302727 | Weighted mode | 0.07 | 0.98 (0.97, 1.00) |
| genus Ruminococcus gnavus group | *TFCP2L1* | GCST90302735 | MR Egger | 0.30 | 1.11 (0.94, 1.32) |
| genus Ruminococcus gnavus group | *TFCP2L1* | GCST90302735 | Weighted median | 0.06 | 1.05 (1.00, 1.11) |
| genus Ruminococcus gnavus group | *TFCP2L1* | GCST90302735 | Inverse variance weighted | 6.36 × 10^-4^ | 1.05 (1.00, 1.10) |
| genus Ruminococcus gnavus group | *TFCP2L1* | GCST90302735 | Simple mode | 0.26 | 1.06 (0.97, 1.16) |
| genus Ruminococcus gnavus group | *TFCP2L1* | GCST90302735 | Weighted mode | 0.15 | 1.06 (1.00, 1.12) |
| genus Ruminococcus gnavus group | *TRIM33* | GCST90302735 | MR Egger | 0.89 | 1.02 (0.85, 1.22) |
| genus Ruminococcus gnavus group | *TRIM33* | GCST90302735 | Weighted median | 0.18 | 1.06 (0.97, 1.16) |
| genus Ruminococcus gnavus group | *TRIM33* | GCST90302735 | Inverse variance weighted | 0.03 | 1.08 (1.01, 1.17) |
| genus Ruminococcus gnavus group | *TRIM33* | GCST90302735 | Simple mode | 0.16 | 1.14 (0.99, 1.32) |
| genus Ruminococcus gnavus group | *TRIM33* | GCST90302735 | Weighted mode | 0.43 | 1.05 (0.95, 1.15) |
| genus Senegalimassilia | *FBN2* | GCST90302795 | MR Egger | 0.40 | 1.01 (0.99, 1.03) |
| genus Senegalimassilia | *FBN2* | GCST90302795 | Weighted median | 0.07 | 1.01 (1.00, 1.03) |
| genus Senegalimassilia | *FBN2* | GCST90302795 | Inverse variance weighted | 0.04 | 1.01 (1.00, 1.02) |
| genus Senegalimassilia | *FBN2* | GCST90302795 | Simple mode | 0.27 | 1.02 (0.99, 1.05) |
| genus Senegalimassilia | *FBN2* | GCST90302795 | Weighted mode | 0.33 | 1.01 (0.99, 1.02) |
| genus Senegalimassilia | *GALT* | GCST90302795 | MR Egger | 0.23 | 0.98 (0.94, 1.01) |
| genus Senegalimassilia | *GALT* | GCST90302795 | Weighted median | 0.03 | 0.97 (0.94, 1.00) |
| genus Senegalimassilia | *GALT* | GCST90302795 | Inverse variance weighted | 6.36 × 10^-4^ | 0.96 (0.94, 0.98) |
| genus Senegalimassilia | *GALT* | GCST90302795 | Simple mode | 0.04 | 0.94 (0.88, 0.99) |
| genus Senegalimassilia | *GALT* | GCST90302795 | Weighted mode | 0.03 | 0.97 (0.94, 0.99) |
| genus Senegalimassilia | *ST6GALNAC1* | GCST90302795 | MR Egger | 0.56 | 0.99 (0.96, 1.02) |
| genus Senegalimassilia | *ST6GALNAC1* | GCST90302795 | Weighted median | 0.20 | 0.98 (0.96, 1.01) |
| genus Senegalimassilia | *ST6GALNAC1* | GCST90302795 | Inverse variance weighted | 0.02 | 0.98 (0.96, 1.00) |
| genus Senegalimassilia | *ST6GALNAC1* | GCST90302795 | Simple mode | 0.08 | 0.96 (0.92, 1.00) |
| genus Senegalimassilia | *ST6GALNAC1* | GCST90302795 | Weighted mode | 0.10 | 0.98 (0.95, 1.00) |
| genus Tyzzerella3 | *ABCA7* | GCST90302660 | MR Egger | 0.19 | 1.02 (0.99, 1.05) |
| genus Tyzzerella3 | *ABCA7* | GCST90302660 | Weighted median | 0.01 | 1.03 (1.01, 1.06) |
| genus Tyzzerella3 | *ABCA7* | GCST90302660 | Inverse variance weighted | 0.01 | 1.02 (1.01, 1.04) |
| genus Tyzzerella3 | *ABCA7* | GCST90302660 | Simple mode | 0.32 | 1.03 (0.98, 1.08) |
| genus Tyzzerella3 | *ABCA7* | GCST90302660 | Weighted mode | 0.04 | 1.04 (1.00, 1.07) |
| genus Tyzzerella3 | *CNN2* | GCST90302660 | MR Egger | 0.01 | 1.03 (1.01, 1.05) |
| genus Tyzzerella3 | *CNN2* | GCST90302660 | Weighted median | 0.02 | 1.02 (1.00, 1.04) |
| genus Tyzzerella3 | *CNN2* | GCST90302660 | Inverse variance weighted | 0.01 | 1.02 (1.00, 1.03) |
| genus Tyzzerella3 | *CNN2* | GCST90302660 | Simple mode | 0.25 | 1.02 (0.99, 1.05) |
| genus Tyzzerella3 | *CNN2* | GCST90302660 | Weighted mode | 0.02 | 1.02 (1.00, 1.04) |
| genus Tyzzerella3 | *FARS2* | GCST90302660 | MR Egger | 0.15 | 1.03 (0.99, 1.06) |
| genus Tyzzerella3 | *FARS2* | GCST90302660 | Weighted median | 0.03 | 1.03 (1.00, 1.06) |
| genus Tyzzerella3 | *FARS2* | GCST90302660 | Inverse variance weighted | 0.01 | 1.03 (1.01, 1.05) |
| genus Tyzzerella3 | *FARS2* | GCST90302660 | Simple mode | 0.46 | 1.02 (0.97, 1.06) |
| genus Tyzzerella3 | *FARS2* | GCST90302660 | Weighted mode | 0.15 | 1.02 (0.99, 1.06) |
| genus Tyzzerella3 | *POLR2E* | GCST90302660 | MR Egger | 0.32 | 1.02 (0.98, 1.07) |
| genus Tyzzerella3 | *POLR2E* | GCST90302660 | Weighted median | 0.06 | 1.03 (1.00, 1.06) |
| genus Tyzzerella3 | *POLR2E* | GCST90302660 | Inverse variance weighted | 4.18 × 10^-4^ | 1.04 (1.02, 1.06) |
| genus Tyzzerella3 | *POLR2E* | GCST90302660 | Simple mode | 0.22 | 1.04 (0.98, 1.10) |
| genus Tyzzerella3 | *POLR2E* | GCST90302660 | Weighted mode | 0.19 | 1.02 (0.99, 1.06) |
| genus Tyzzerella3 | *VRK2* | GCST90302660 | MR Egger | 0.42 | 1.02 (0.97, 1.07) |
| genus Tyzzerella3 | *VRK2* | GCST90302660 | Weighted median | 0.30 | 1.02 (0.98, 1.06) |
| genus Tyzzerella3 | *VRK2* | GCST90302660 | Inverse variance weighted | 0.04 | 1.03 (1.00, 1.05) |
| genus Tyzzerella3 | *VRK2* | GCST90302660 | Simple mode | 0.46 | 0.97 (0.90, 1.05) |
| genus Tyzzerella3 | *VRK2* | GCST90302660 | Weighted mode | 0.45 | 1.02 (0.98, 1.06) |
| genus Veillonella | *ACCS* | GCST90302696 | MR Egger | 0.10 | 0.99 (0.98, 1.00) |
| genus Veillonella | *ACCS* | GCST90302696 | Weighted median | 2.45 × 10^-3^ | 0.98 (0.97, 0.99) |
| genus Veillonella | *ACCS* | GCST90302696 | Inverse variance weighted | 1.53 × 10^-4^ | 0.99 (0.98, 0.99) |
| genus Veillonella | *ACCS* | GCST90302696 | Simple mode | 0.14 | 0.99 (0.97, 1.00) |
| genus Veillonella | *ACCS* | GCST90302696 | Weighted mode | 0.01 | 0.99 (0.97, 1.00) |
| genus Veillonella | *KIAA0930* | GCST90302696 | MR Egger | 0.11 | 1.05 (0.99, 1.11) |
| genus Veillonella | *KIAA0930* | GCST90302696 | Weighted median | 0.09 | 1.03 (1.00, 1.07) |
| genus Veillonella | *KIAA0930* | GCST90302696 | Inverse variance weighted | 0.03 | 1.03 (1.00, 1.06) |
| genus Veillonella | *KIAA0930* | GCST90302696 | Simple mode | 0.24 | 1.03 (0.98, 1.09) |
| genus Veillonella | *KIAA0930* | GCST90302696 | Weighted mode | 0.16 | 1.03 (0.99, 1.08) |
| genus Veillonella | *NAP1L4* | GCST90302696 | MR Egger | 0.99 | 1.00 (0.95, 1.06) |
| genus Veillonella | *NAP1L4* | GCST90302696 | Weighted median | 0.15 | 1.03 (0.99, 1.06) |
| genus Veillonella | *NAP1L4* | GCST90302696 | Inverse variance weighted | 0.03 | 1.03 (1.00, 1.06) |
| genus Veillonella | *NAP1L4* | GCST90302696 | Simple mode | 0.43 | 1.02 (0.97, 1.08) |
| genus Veillonella | *NAP1L4* | GCST90302696 | Weighted mode | 0.28 | 1.02 (0.98, 1.07) |
| genus Veillonella | *SLC22A18* | GCST90302696 | MR Egger | 0.42 | 1.01 (0.98, 1.04) |
| genus Veillonella | *SLC22A18* | GCST90302696 | Weighted median | 0.05 | 1.02 (1.00, 1.04) |
| genus Veillonella | *SLC22A18* | GCST90302696 | Inverse variance weighted | 0.04 | 1.02 (1.00, 1.04) |
| genus Veillonella | *SLC22A18* | GCST90302696 | Simple mode | 0.37 | 1.02 (0.97, 1.08) |
| genus Veillonella | *SLC22A18* | GCST90302696 | Weighted mode | 0.08 | 1.02 (1.00, 1.04) |
| genus Veillonella | *SLC22A18AS* | GCST90302696 | MR Egger | 0.05 | 1.03 (1.00, 1.05) |
| genus Veillonella | *SLC22A18AS* | GCST90302696 | Weighted median | 0.37 | 1.01 (0.99, 1.03) |
| genus Veillonella | *SLC22A18AS* | GCST90302696 | Inverse variance weighted | 0.01 | 1.02 (1.00, 1.03) |
| genus Veillonella | *SLC22A18AS* | GCST90302696 | Simple mode | 0.05 | 1.04 (1.00, 1.08) |
| genus Veillonella | *SLC22A18AS* | GCST90302696 | Weighted mode | 0.21 | 1.02 (0.99, 1.04) |
| genus Veillonella | *TOR3A* | GCST90302696 | MR Egger | 0.27 | 1.03 (0.98, 1.09) |
| genus Veillonella | *TOR3A* | GCST90302696 | Weighted median | 0.09 | 1.03 (1.00, 1.06) |
| genus Veillonella | *TOR3A* | GCST90302696 | Inverse variance weighted | 2.58× 10^-3^ | 1.03 (1.01, 1.06) |
| genus Veillonella | *TOR3A* | GCST90302696 | Simple mode | 0.23 | 1.03 (0.98, 1.08) |
| genus Veillonella | *TOR3A* | GCST90302696 | Weighted mode | 0.23 | 1.02 (0.99, 1.06) |
| order Desulfovibrionales | *ACSS2* | GCST90302676 | MR Egger | 0.54 | 0.83 (0.54, 1.26) |
| order Desulfovibrionales | *ACSS2* | GCST90302676 | Weighted median | 1.14 × 10^-3^ | 0.83 (0.74, 0.93) |
| order Desulfovibrionales | *ACSS2* | GCST90302676 | Inverse variance weighted | 1.24 ×10^-4^ | 0.83 (0.75, 0.91) |
| order Desulfovibrionales | *ACSS2* | GCST90302676 | Simple mode | 0.12 | 0.83 (0.73, 0.95) |
| order Desulfovibrionales | *ACSS2* | GCST90302676 | Weighted mode | 0.12 | 0.83 (0.72, 0.95) |
| order Desulfovibrionales | *CPNE1* | GCST90302676 | MR Egger | 0.01 | 0.98 (0.97, 0.99) |
| order Desulfovibrionales | *CPNE1* | GCST90302676 | Weighted median | 0.01 | 0.98 (0.97, 1.00) |
| order Desulfovibrionales | *CPNE1* | GCST90302676 | Inverse variance weighted | 6.51 × 10^-5^ | 0.98 (0.97, 0.99) |
| order Desulfovibrionales | *CPNE1* | GCST90302676 | Simple mode | 0.04 | 0.97 (0.95, 1.00) |
| order Desulfovibrionales | *CPNE1* | GCST90302676 | Weighted mode | 1.20 × 10^-3^ | 0.98 (0.97, 0.99) |
| order Desulfovibrionales | *EDEM2* | GCST90302676 | MR Egger | 0.31 | 0.98 (0.94, 1.02) |
| order Desulfovibrionales | *EDEM2* | GCST90302676 | Weighted median | 3.17 × 10^-3^ | 0.96 (0.93, 0.99) |
| order Desulfovibrionales | *EDEM2* | GCST90302676 | Inverse variance weighted | 3.74 × 10^-5^ | 0.95 (0.93, 0.97) |
| order Desulfovibrionales | *EDEM2* | GCST90302676 | Simple mode | 0.07 | 0.94 (0.89, 1.00) |
| order Desulfovibrionales | *EDEM2* | GCST90302676 | Weighted mode | 0.01 | 0.96 (0.93, 0.99) |
| order Desulfovibrionales | *EIF2S2* | GCST90302676 | MR Egger | 0.64 | 1.02 (0.95, 1.08) |
| order Desulfovibrionales | *EIF2S2* | GCST90302676 | Weighted median | 3.75 × 10^-3^ | 1.03 (1.00, 1.06) |
| order Desulfovibrionales | *EIF2S2* | GCST90302676 | Inverse variance weighted | 3.75 × 10^-3^ | 1.04 (1.01, 1.06) |
| order Desulfovibrionales | *EIF2S2* | GCST90302676 | Simple mode | 0.07 | 1.05 (1.00, 1.11) |
| order Desulfovibrionales | *EIF2S2* | GCST90302676 | Weighted mode | 0.09 | 1.03 (1.00, 1.06) |
| order Desulfovibrionales | *EIF6* | GCST90302676 | MR Egger | 0.29 | 1.01 (0.99, 1.04) |
| order Desulfovibrionales | *EIF6* | GCST90302676 | Weighted median | 0.02 | 1.02 (1.00, 1.04) |
| order Desulfovibrionales | *EIF6* | GCST90302676 | Inverse variance weighted | 2.40 × 10^-3^ | 1.03 (1.01, 1.04) |
| order Desulfovibrionales | *EIF6* | GCST90302676 | Simple mode | 0.19 | 1.02 (0.99, 1.05) |
| order Desulfovibrionales | *EIF6* | GCST90302676 | Weighted mode | 0.06 | 1.02 (1.00, 1.04) |
| order Desulfovibrionales | *ITCH* | GCST90302676 | MR Egger | 0.76 | 1.01 (0.93, 1.11) |
| order Desulfovibrionales | *ITCH* | GCST90302676 | Weighted median | 0.01 | 1.07 (1.01, 1.12) |
| order Desulfovibrionales | *ITCH* | GCST90302676 | Inverse variance weighted | 1.90 × 10^-5^ | 1.10 (1.05, 1.15) |
| order Desulfovibrionales | *ITCH* | GCST90302676 | Simple mode | 0.26 | 1.06 (0.96, 1.17) |
| order Desulfovibrionales | *ITCH* | GCST90302676 | Weighted mode | 0.02 | 1.06 (1.01, 1.11) |
| order Desulfovibrionales | *MAP1LC3A* | GCST90302676 | MR Egger | 0.43 | 0.99 (0.96, 1.02) |
| order Desulfovibrionales | *MAP1LC3A* | GCST90302676 | Weighted median | 0.19 | 1.01 (0.99, 1.04) |
| order Desulfovibrionales | *MAP1LC3A* | GCST90302676 | Inverse variance weighted | 0.01 | 1.03 (1.01, 1.05) |
| order Desulfovibrionales | *MAP1LC3A* | GCST90302676 | Simple mode | 0.16 | 1.03 (0.99, 1.07) |
| order Desulfovibrionales | *MAP1LC3A* | GCST90302676 | Weighted mode | 0.12 | 1.02 (1.00, 1.04) |
| order Desulfovibrionales | *MAP1LC3A* | GCST90302676 | MR-PRESSO | 0.01 | 0.00 (0.00, 0.00) |
| order Desulfovibrionales | *MMP24* | GCST90302676 | MR Egger | 0.86 | 1.01 (0.90, 1.13) |
| order Desulfovibrionales | *MMP24* | GCST90302676 | Weighted median | 1.66 × 10^-3^ | 0.92 (0.88, 0.97) |
| order Desulfovibrionales | *MMP24* | GCST90302676 | Inverse variance weighted | 1.53 × 10^-3^ | 0.92 (0.88, 0.97) |
| order Desulfovibrionales | *MMP24* | GCST90302676 | Simple mode | 0.07 | 0.92 (0.86, 0.99) |
| order Desulfovibrionales | *MMP24* | GCST90302676 | Weighted mode | 0.02 | 0.92 (0.88, 0.97) |
| order Desulfovibrionales | *MYH7B* | GCST90302676 | MR Egger | 0.38 | 1.07 (0.93, 1.22) |
| order Desulfovibrionales | *MYH7B* | GCST90302676 | Weighted median | 7.69 × 10^-5^ | 1.13 (1.06, 1.20) |
| order Desulfovibrionales | *MYH7B* | GCST90302676 | Inverse variance weighted | 4.03 × 10^-9^ | 1.15 (1.10, 1.20) |
| order Desulfovibrionales | *MYH7B* | GCST90302676 | Simple mode | 0.02 | 1.12 (1.04, 1.22) |
| order Desulfovibrionales | *MYH7B* | GCST90302676 | Weighted mode | 0.01 | 1.13 (1.04, 1.21) |
| order Desulfovibrionales | *PIGU* | GCST90302676 | MR Egger | 0.45 | 1.09 (0.89, 1.34) |
| order Desulfovibrionales | *PIGU* | GCST90302676 | Weighted median | 0.19 | 1.06 (0.97, 1.15) |
| order Desulfovibrionales | *PIGU* | GCST90302676 | Inverse variance weighted | 0.04 | 1.08 (1.01, 1.15) |
| order Desulfovibrionales | *PIGU* | GCST90302676 | Simple mode | 0.49 | 1.05 (0.93, 1.18) |
| order Desulfovibrionales | *PIGU* | GCST90302676 | Weighted mode | 0.46 | 1.05 (0.93, 1.18) |
| order Desulfovibrionales | *PROCR* | GCST90302676 | MR Egger | 0.91 | 1.01 (0.87, 1.17) |
| order Desulfovibrionales | *PROCR* | GCST90302676 | Weighted median | 8.60 × 10^-4^ | 0.90 (0.84, 0.96) |
| order Desulfovibrionales | *PROCR* | GCST90302676 | Inverse variance weighted | 4.89 ×10^-5^ | 0.89 (0.84, 0.94) |
| order Desulfovibrionales | *PROCR* | GCST90302676 | Simple mode | 0.08 | 0.89 (0.81, 0.99) |
| order Desulfovibrionales | *PROCR* | GCST90302676 | Weighted mode | 0.03 | 0.89 (0.83, 0.96) |
| order Desulfovibrionales | *MAP1LC3A* | GCST90302677 | MR Egger | 0.39 | 0.99 (0.96, 1.01) |
| order Desulfovibrionales | *MAP1LC3A* | GCST90302677 | Weighted median | 0.21 | 1.01 (0.99, 1.04) |
| order Desulfovibrionales | *MAP1LC3A* | GCST90302677 | Inverse variance weighted | 1.11 × 10^-3^ | 1.03 (1.01, 1.05) |
| order Desulfovibrionales | *MAP1LC3A* | GCST90302677 | Simple mode | 0.30 | 1.03 (0.98, 1.08) |
| order Desulfovibrionales | *MAP1LC3A* | GCST90302677 | Weighted mode | 0.15 | 1.02 (1.00, 1.04) |
| order Desulfovibrionales | *MAP1LC3A* | GCST90302677 | MR-PRESSO | 3.21 × 10^-3^ | 0.00 (0.00, 0.00) |
| order Desulfovibrionales | *MMP24* | GCST90302677 | MR Egger | 0.94 | 0.99 (0.87, 1.14) |
| order Desulfovibrionales | *MMP24* | GCST90302677 | Weighted median | 0.01 | 0.93 (0.88, 0.98) |
| order Desulfovibrionales | *MMP24* | GCST90302677 | Inverse variance weighted | 0.02 | 0.94 (0.89, 0.99) |
| order Desulfovibrionales | *MMP24* | GCST90302677 | Simple mode | 0.16 | 0.93 (0.86, 1.01) |
| order Desulfovibrionales | *MMP24* | GCST90302677 | Weighted mode | 5.51 × 10^-5^ | 0.93 (0.88, 0.99) |
| order Desulfovibrionales | *MYH7B* | GCST90302677 | MR Egger | 0.43 | 1.06 (0.92, 1.23) |
| order Desulfovibrionales | *MYH7B* | GCST90302677 | Weighted median | 5.51 × 10^-5^ | 1.14 (1.07, 1.21) |
| order Desulfovibrionales | *MYH7B* | GCST90302677 | Inverse variance weighted | 9.38 × 10^-9^ | 1.15 (1.10, 1.21) |
| order Desulfovibrionales | *MYH7B* | GCST90302677 | Simple mode | 0.03 | 1.14 (1.03, 1.25) |
| order Desulfovibrionales | *MYH7B* | GCST90302677 | Weighted mode | 0.01 | 1.13 (1.05, 1.23) |
| order Desulfovibrionales | *PROCR* | GCST90302677 | MR Egger | 0.75 | 0.97 (0.84, 1.14) |
| order Desulfovibrionales | *PROCR* | GCST90302677 | Weighted median | 0.01 | 0.91 (0.85, 0.97) |
| order Desulfovibrionales | *PROCR* | GCST90302677 | Inverse variance weighted | 8.71 × 10^-5^ | 0.89 (0.85, 0.95) |
| order Desulfovibrionales | *PROCR* | GCST90302677 | Simple mode | 0.09 | 0.91 (0.83, 1.00) |
| order Desulfovibrionales | *PROCR* | GCST90302677 | Weighted mode | 1.04 × 10^-3^ | 0.91 (0.85, 0.98) |
| order Desulfovibrionales | *ACSS2* | GCST90302677 | MR Egger | 0.69 | 0.89 (0.57, 1.38) |
| order Desulfovibrionales | *ACSS2* | GCST90302677 | Weighted median | 0.01 | 0.85 (0.75, 0.96) |
| order Desulfovibrionales | *ACSS2* | GCST90302677 | Inverse variance weighted | 1.04 × 10^-3^ | 0.84 (0.76, 0.93) |
| order Desulfovibrionales | *ACSS2* | GCST90302677 | Simple mode | 0.14 | 0.84 (0.72, 0.97) |
| order Desulfovibrionales | *ACSS2* | GCST90302677 | Weighted mode | 0.18 | 0.86 (0.74, 1.00) |
| order Desulfovibrionales | *CPNE1* | GCST90302677 | MR Egger | 1.32 × 10^-3^ | 0.98 (0.96, 0.99) |
| order Desulfovibrionales | *CPNE1* | GCST90302677 | Weighted median | 8.66 × 10^-5^ | 0.98 (0.96, 0.99) |
| order Desulfovibrionales | *CPNE1* | GCST90302677 | Inverse variance weighted | 2.68 × 10^-6^ | 0.98 (0.97, 0.99) |
| order Desulfovibrionales | *CPNE1* | GCST90302677 | Simple mode | 0.08 | 0.98 (0.96, 1.00) |
| order Desulfovibrionales | *CPNE1* | GCST90302677 | Weighted mode | 2.80 × 10^-4^ | 0.98 (0.97, 0.99) |
| order Desulfovibrionales | *EDEM2* | GCST90302677 | MR Egger | 0.31 | 0.98 (0.94, 1.02) |
| order Desulfovibrionales | *EDEM2* | GCST90302677 | Weighted median | 0.01 | 0.96 (0.94, 0.99) |
| order Desulfovibrionales | *EDEM2* | GCST90302677 | Inverse variance weighted | 1.83 × 10^-7^ | 0.94 (0.92, 0.96) |
| order Desulfovibrionales | *EDEM2* | GCST90302677 | Simple mode | 0.01 | 0.92 (0.87, 0.98) |
| order Desulfovibrionales | *EDEM2* | GCST90302677 | Weighted mode | 3.96 × 10^-3^ | 0.95 (0.93, 0.98) |
| order Desulfovibrionales | *EIF2S2* | GCST90302677 | MR Egger | 0.29 | 1.03 (0.97, 1.09) |
| order Desulfovibrionales | *EIF2S2* | GCST90302677 | Weighted median | 0.01 | 1.04 (1.01, 1.08) |
| order Desulfovibrionales | *EIF2S2* | GCST90302677 | Inverse variance weighted | 1.20 × 10^-5^ | 1.05 (1.03, 1.07) |
| order Desulfovibrionales | *EIF2S2* | GCST90302677 | Simple mode | 0.04 | 1.06 (1.00, 1.12) |
| order Desulfovibrionales | *EIF2S2* | GCST90302677 | Weighted mode | 0.01 | 1.05 (1.01, 1.08) |
| order Desulfovibrionales | *EIF6* | GCST90302677 | MR Egger | 0.18 | 1.02 (0.99, 1.05) |
| order Desulfovibrionales | *EIF6* | GCST90302677 | Weighted median | 0.03 | 1.02 (1.00, 1.04) |
| order Desulfovibrionales | *EIF6* | GCST90302677 | Inverse variance weighted | 0.01 | 1.02 (1.01, 1.04) |
| order Desulfovibrionales | *EIF6* | GCST90302677 | Simple mode | 0.06 | 1.03 (1.00, 1.07) |
| order Desulfovibrionales | *EIF6* | GCST90302677 | Weighted mode | 0.03 | 1.02 (1.00, 1.04) |
| order Desulfovibrionales | *GSS* | GCST90302677 | MR Egger | 0.62 | 1.07 (0.85, 1.34) |
| order Desulfovibrionales | *GSS* | GCST90302677 | Weighted median | 0.15 | 0.91 (0.80, 1.03) |
| order Desulfovibrionales | *GSS* | GCST90302677 | Inverse variance weighted | 0.04 | 0.87 (0.76, 1.00) |
| order Desulfovibrionales | *GSS* | GCST90302677 | Simple mode | 0.32 | 0.91 (0.78, 1.06) |
| order Desulfovibrionales | *GSS* | GCST90302677 | Weighted mode | 0.30 | 0.91 (0.79, 1.05) |
| order Desulfovibrionales | *HP* | GCST90302677 | MR Egger | 0.10 | 0.99 (0.97, 1.00) |
| order Desulfovibrionales | *HP* | GCST90302677 | Weighted median | 0.28 | 0.99 (0.98, 1.01) |
| order Desulfovibrionales | *HP* | GCST90302677 | Inverse variance weighted | 0.01 | 0.99 (0.98, 1.00) |
| order Desulfovibrionales | *HP* | GCST90302677 | Simple mode | 0.38 | 0.99 (0.96, 1.02) |
| order Desulfovibrionales | *HP* | GCST90302677 | Weighted mode | 0.26 | 0.99 (0.98, 1.01) |
| order Desulfovibrionales | *IMPAD1* | GCST90302677 | MR Egger | 0.46 | 1.08 (0.90, 1.30) |
| order Desulfovibrionales | *IMPAD1* | GCST90302677 | Weighted median | 0.01 | 1.10 (1.02, 1.18) |
| order Desulfovibrionales | *IMPAD1* | GCST90302677 | Inverse variance weighted | 0.05 | 1.08 (1.00, 1.17) |
| order Desulfovibrionales | *IMPAD1* | GCST90302677 | Simple mode | 0.14 | 1.11 (0.98, 1.26) |
| order Desulfovibrionales | *IMPAD1* | GCST90302677 | Weighted mode | 0.05 | 1.11 (1.02, 1.21) |
| order Desulfovibrionales | *ITCH* | GCST90302677 | MR Egger | 0.63 | 1.02 (0.94, 1.12) |
| order Desulfovibrionales | *ITCH* | GCST90302677 | Weighted median | 0.01 | 1.07 (1.02, 1.12) |
| order Desulfovibrionales | *ITCH* | GCST90302677 | Inverse variance weighted | 6.55 × 10^-6^ | 1.10 (1.06, 1.15) |
| order Desulfovibrionales | *ITCH* | GCST90302677 | Simple mode | 0.02 | 1.17 (1.05, 1.30) |
| order Desulfovibrionales | *ITCH* | GCST90302677 | Weighted mode | 0.03 | 1.07 (1.02, 1.13) |
| order Desulfovibrionales | *ACSS2* | GCST90302717 | MR Egger | 0.86 | 0.95 (0.62, 1.47) |
| order Desulfovibrionales | *ACSS2* | GCST90302717 | Weighted median | 0.01 | 0.85 (0.75, 0.96) |
| order Desulfovibrionales | *ACSS2* | GCST90302717 | Inverse variance weighted | 3.18 × 10^-4^ | 0.84 (0.76, 0.92) |
| order Desulfovibrionales | *ACSS2* | GCST90302717 | Simple mode | 0.20 | 0.86 (0.74, 1.01) |
| order Desulfovibrionales | *ACSS2* | GCST90302717 | Weighted mode | 0.25 | 0.87 (0.74, 1.03) |
| order Desulfovibrionales | *CPNE1* | GCST90302717 | MR Egger | 0.01 | 0.98 (0.97, 1.00) |
| order Desulfovibrionales | *CPNE1* | GCST90302717 | Weighted median | 4.76 × 10^-3^ | 0.98 (0.97, 1.00) |
| order Desulfovibrionales | *CPNE1* | GCST90302717 | Inverse variance weighted | 1.19 × 10^-3^ | 0.99 (0.98, 0.99) |
| order Desulfovibrionales | *CPNE1* | GCST90302717 | Simple mode | 0.28 | 0.99 (0.97, 1.01) |
| order Desulfovibrionales | *CPNE1* | GCST90302717 | Weighted mode | 0.01 | 0.99 (0.98, 1.00) |
| order Desulfovibrionales | *ECHDC3* | GCST90302717 | MR Egger | 0.59 | 0.99 (0.97, 1.02) |
| order Desulfovibrionales | *ECHDC3* | GCST90302717 | Weighted median | 0.06 | 0.98 (0.96, 1.00) |
| order Desulfovibrionales | *ECHDC3* | GCST90302717 | Inverse variance weighted | 2.67 × 10^-3^ | 0.98 (0.97, 0.99) |
| order Desulfovibrionales | *ECHDC3* | GCST90302717 | Simple mode | 0.16 | 0.97 (0.94, 1.01) |
| order Desulfovibrionales | *ECHDC3* | GCST90302717 | Weighted mode | 0.09 | 0.98 (0.96, 1.00) |
| order Desulfovibrionales | *EDEM2* | GCST90302717 | MR Egger | 0.35 | 0.97 (0.92, 1.03) |
| order Desulfovibrionales | *EDEM2* | GCST90302717 | Weighted median | 0.01 | 0.96 (0.94, 0.99) |
| order Desulfovibrionales | *EDEM2* | GCST90302717 | Inverse variance weighted | 0.01 | 0.96 (0.93, 0.99) |
| order Desulfovibrionales | *EDEM2* | GCST90302717 | Simple mode | 0.10 | 0.96 (0.91, 1.01) |
| order Desulfovibrionales | *EDEM2* | GCST90302717 | Weighted mode | 0.02 | 0.96 (0.94, 0.99) |
| order Desulfovibrionales | *EIF6* | GCST90302717 | MR Egger | 0.30 | 1.02 (0.99, 1.05) |
| order Desulfovibrionales | *EIF6* | GCST90302717 | Weighted median | 0.02 | 1.02 (1.00, 1.05) |
| order Desulfovibrionales | *EIF6* | GCST90302717 | Inverse variance weighted | 0.01 | 1.03 (1.01, 1.05) |
| order Desulfovibrionales | *EIF6* | GCST90302717 | Simple mode | 0.19 | 1.02 (0.99, 1.05) |
| order Desulfovibrionales | *EIF6* | GCST90302717 | Weighted mode | 0.03 | 1.02 (1.00, 1.04) |
| order Desulfovibrionales | *HP* | GCST90302717 | MR Egger | 0.28 | 0.99 (0.98, 1.01) |
| order Desulfovibrionales | *HP* | GCST90302717 | Weighted median | 0.27 | 0.99 (0.98, 1.01) |
| order Desulfovibrionales | *HP* | GCST90302717 | Inverse variance weighted | 3.66 × 10^-3^ | 0.99 (0.98, 1.00) |
| order Desulfovibrionales | *HP* | GCST90302717 | Simple mode | 0.21 | 0.98 (0.96, 1.01) |
| order Desulfovibrionales | *HP* | GCST90302717 | Weighted mode | 0.35 | 0.99 (0.98, 1.01) |
| order Desulfovibrionales | *ITCH* | GCST90302717 | MR Egger | 0.76 | 1.02 (0.91, 1.13) |
| order Desulfovibrionales | *ITCH* | GCST90302717 | Weighted median | 0.03 | 1.06 (1.00, 1.11) |
| order Desulfovibrionales | *ITCH* | GCST90302717 | Inverse variance weighted | 2.99 × 10^-3^ | 1.07 (1.02, 1.12) |
| order Desulfovibrionales | *ITCH* | GCST90302717 | Simple mode | 0.21 | 1.08 (0.97, 1.20) |
| order Desulfovibrionales | *ITCH* | GCST90302717 | Weighted mode | 0.08 | 1.06 (1.00, 1.12) |
| order Desulfovibrionales | *MMP24* | GCST90302717 | MR Egger | 0.49 | 1.05 (0.93, 1.18) |
| order Desulfovibrionales | *MMP24* | GCST90302717 | Weighted median | 0.03 | 0.95 (0.90, 1.00) |
| order Desulfovibrionales | *MMP24* | GCST90302717 | Inverse variance weighted | 0.02 | 0.93 (0.88, 0.99) |
| order Desulfovibrionales | *MMP24* | GCST90302717 | Simple mode | 0.12 | 0.94 (0.87, 1.00) |
| order Desulfovibrionales | *MMP24* | GCST90302717 | Weighted mode | 0.09 | 0.94 (0.89, 1.00) |
| order Desulfovibrionales | *MYH7B* | GCST90302717 | MR Egger | 0.43 | 1.07 (0.91, 1.26) |
| order Desulfovibrionales | *MYH7B* | GCST90302717 | Weighted median | 5.88 × 10^-4^ | 1.11 (1.05, 1.18) |
| order Desulfovibrionales | *MYH7B* | GCST90302717 | Inverse variance weighted | 8.13 × 10^-6^ | 1.13 (1.07, 1.19) |
| order Desulfovibrionales | *MYH7B* | GCST90302717 | Simple mode | 0.03 | 1.11 (1.02, 1.21) |
| order Desulfovibrionales | *MYH7B* | GCST90302717 | Weighted mode | 0.02 | 1.11 (1.03, 1.20) |
| order Desulfovibrionales | *PIGU* | GCST90302717 | MR Egger | 0.30 | 1.15 (0.93, 1.43) |
| order Desulfovibrionales | *PIGU* | GCST90302717 | Weighted median | 2.82 × 10^-4^ | 1.17 (1.08, 1.28) |
| order Desulfovibrionales | *PIGU* | GCST90302717 | Inverse variance weighted | 1.84 × 10^-4^ | 1.14 (1.06, 1.22) |
| order Desulfovibrionales | *PIGU* | GCST90302717 | Simple mode | 0.09 | 1.17 (1.02, 1.33) |
| order Desulfovibrionales | *PIGU* | GCST90302717 | Weighted mode | 0.04 | 1.18 (1.06, 1.31) |
| order Desulfovibrionales | *PROCR* | GCST90302717 | MR Egger | 0.83 | 0.98 (0.85, 1.14) |
| order Desulfovibrionales | *PROCR* | GCST90302717 | Weighted median | 0.01 | 0.92 (0.86, 0.98) |
| order Desulfovibrionales | *PROCR* | GCST90302717 | Inverse variance weighted | 9.70 × 10^-5^ | 0.90 (0.85, 0.95) |
| order Desulfovibrionales | *PROCR* | GCST90302717 | Simple mode | 0.11 | 0.91 (0.83, 1.00) |
| order Desulfovibrionales | *PROCR* | GCST90302717 | Weighted mode | 0.05 | 0.91 (0.86, 0.98) |
| order Desulfovibrionales | *ZNF19* | GCST90302717 | MR Egger | 0.42 | 0.83 (0.57, 1.20) |
| order Desulfovibrionales | *ZNF19* | GCST90302717 | Weighted median | 0.12 | 1.11 (0.97, 1.26) |
| order Desulfovibrionales | *ZNF19* | GCST90302717 | Inverse variance weighted | 0.02 | 1.18 (1.02, 1.36) |
| order Desulfovibrionales | *ZNF19* | GCST90302717 | Simple mode | 0.42 | 1.10 (0.90, 1.36) |
| order Desulfovibrionales | *ZNF19* | GCST90302717 | Weighted mode | 0.42 | 1.08 (0.92, 1.26) |
| order Desulfovibrionales | *ZNF23* | GCST90302717 | MR Egger | 0.20 | 1.03 (0.99, 1.08) |
| order Desulfovibrionales | *ZNF23* | GCST90302717 | Weighted median | 0.49 | 1.01 (0.98, 1.04) |
| order Desulfovibrionales | *ZNF23* | GCST90302717 | Inverse variance weighted | 0.04 | 1.02 (1.00, 1.04) |
| order Desulfovibrionales | *ZNF23* | GCST90302717 | Simple mode | 0.72 | 1.01 (0.96, 1.06) |
| order Desulfovibrionales | *ZNF23* | GCST90302717 | Weighted mode | 0.44 | 1.01 (0.98, 1.04) |
| family Desulfovibrionaceae | *ACSS2* | GCST90302717 | MR Egger | 0.86 | 0.95 (0.62, 1.47) |
| family Desulfovibrionaceae | *ACSS2* | GCST90302717 | Weighted median | 0.01 | 0.85 (0.75, 0.96) |
| family Desulfovibrionaceae | *ACSS2* | GCST90302717 | Inverse variance weighted | 3.18 × 10^-4^ | 0.84 (0.76, 0.92) |
| family Desulfovibrionaceae | *ACSS2* | GCST90302717 | Simple mode | 0.22 | 0.86 (0.73, 1.01) |
| family Desulfovibrionaceae | *ACSS2* | GCST90302717 | Weighted mode | 0.23 | 0.87 (0.74, 1.02) |
| family Desulfovibrionaceae | *CPNE1* | GCST90302717 | MR Egger | 0.01 | 0.98 (0.97, 1.00) |
| family Desulfovibrionaceae | *CPNE1* | GCST90302717 | Weighted median | 4.06 × 10^-3^ | 0.98 (0.97, 0.99) |
| family Desulfovibrionaceae | *CPNE1* | GCST90302717 | Inverse variance weighted | 1.19 × 10^-3^ | 0.99 (0.98, 0.99) |
| family Desulfovibrionaceae | *CPNE1* | GCST90302717 | Simple mode | 0.25 | 0.99 (0.97, 1.01) |
| family Desulfovibrionaceae | *CPNE1* | GCST90302717 | Weighted mode | 0.01 | 0.99 (0.98, 1.00) |
| family Desulfovibrionaceae | *ECHDC3* | GCST90302717 | MR Egger | 0.59 | 0.99 (0.97, 1.02) |
| family Desulfovibrionaceae | *ECHDC3* | GCST90302717 | Weighted median | 0.05 | 0.98 (0.96, 1.00) |
| family Desulfovibrionaceae | *ECHDC3* | GCST90302717 | Inverse variance weighted | 2.67 × 10^-3^ | 0.98 (0.97, 0.99) |
| family Desulfovibrionaceae | *ECHDC3* | GCST90302717 | Simple mode | 0.17 | 0.97 (0.94, 1.01) |
| family Desulfovibrionaceae | *ECHDC3* | GCST90302717 | Weighted mode | 0.09 | 0.98 (0.96, 1.00) |
| family Desulfovibrionaceae | *EDEM2* | GCST90302717 | MR Egger | 0.35 | 0.97 (0.92, 1.03) |
| family Desulfovibrionaceae | *EDEM2* | GCST90302717 | Weighted median | 0.01 | 0.96 (0.94, 0.99) |
| family Desulfovibrionaceae | *EDEM2* | GCST90302717 | Inverse variance weighted | 0.01 | 0.96 (0.93, 0.99) |
| family Desulfovibrionaceae | *EDEM2* | GCST90302717 | Simple mode | 0.11 | 0.96 (0.91, 1.01) |
| family Desulfovibrionaceae | *EDEM2* | GCST90302717 | Weighted mode | 0.03 | 0.96 (0.94, 0.99) |
| family Desulfovibrionaceae | *EIF6* | GCST90302717 | MR Egger | 0.30 | 1.02 (0.99, 1.05) |
| family Desulfovibrionaceae | *EIF6* | GCST90302717 | Weighted median | 0.02 | 1.02 (1.00, 1.05) |
| family Desulfovibrionaceae | *EIF6* | GCST90302717 | Inverse variance weighted | 0.01 | 1.03 (1.01, 1.05) |
| family Desulfovibrionaceae | *EIF6* | GCST90302717 | Simple mode | 0.17 | 1.02 (0.99, 1.05) |
| family Desulfovibrionaceae | *EIF6* | GCST90302717 | Weighted mode | 0.03 | 1.02 (1.00, 1.04) |
| family Desulfovibrionaceae | *HP* | GCST90302717 | MR Egger | 0.28 | 0.99 (0.98, 1.01) |
| family Desulfovibrionaceae | *HP* | GCST90302717 | Weighted median | 0.28 | 0.99 (0.98, 1.01) |
| family Desulfovibrionaceae | *HP* | GCST90302717 | Inverse variance weighted | 3.66 × 10^-3^ | 0.99 (0.98, 1.00) |
| family Desulfovibrionaceae | *HP* | GCST90302717 | Simple mode | 0.22 | 0.98 (0.96, 1.01) |
| family Desulfovibrionaceae | *HP* | GCST90302717 | Weighted mode | 0.38 | 0.99 (0.98, 1.01) |
| family Desulfovibrionaceae | *ITCH* | GCST90302717 | MR Egger | 0.76 | 1.02 (0.91, 1.13) |
| family Desulfovibrionaceae | *ITCH* | GCST90302717 | Weighted median | 0.03 | 1.06 (1.00, 1.11) |
| family Desulfovibrionaceae | *ITCH* | GCST90302717 | Inverse variance weighted | 2.99 × 10^-3^ | 1.07 (1.02, 1.12) |
| family Desulfovibrionaceae | *ITCH* | GCST90302717 | Simple mode | 0.21 | 1.08 (0.97, 1.20) |
| family Desulfovibrionaceae | *ITCH* | GCST90302717 | Weighted mode | 0.07 | 1.06 (1.00, 1.11) |
| family Desulfovibrionaceae | *MMP24* | GCST90302717 | MR Egger | 0.49 | 1.05 (0.93, 1.18) |
| family Desulfovibrionaceae | *MMP24* | GCST90302717 | Weighted median | 0.03 | 0.95 (0.90, 1.00) |
| family Desulfovibrionaceae | *MMP24* | GCST90302717 | Inverse variance weighted | 0.02 | 0.93 (0.88, 0.99) |
| family Desulfovibrionaceae | *MMP24* | GCST90302717 | Simple mode | 0.13 | 0.94 (0.87, 1.01) |
| family Desulfovibrionaceae | *MMP24* | GCST90302717 | Weighted mode | 0.08 | 0.94 (0.90, 1.00) |
| family Desulfovibrionaceae | *MYH7B* | GCST90302717 | MR Egger | 0.43 | 1.07 (0.91, 1.26) |
| family Desulfovibrionaceae | *MYH7B* | GCST90302717 | Weighted median | 8.01 × 10^-4^ | 1.11 (1.05, 1.18) |
| family Desulfovibrionaceae | *MYH7B* | GCST90302717 | Inverse variance weighted | 8.13 × 10^-6^ | 1.13 (1.07, 1.19) |
| family Desulfovibrionaceae | *MYH7B* | GCST90302717 | Simple mode | 0.03 | 1.11 (1.03, 1.21) |
| family Desulfovibrionaceae | *MYH7B* | GCST90302717 | Weighted mode | 0.02 | 1.11 (1.04, 1.20) |
| family Desulfovibrionaceae | *PIGU* | GCST90302717 | MR Egger | 0.30 | 1.15 (0.93, 1.43) |
| family Desulfovibrionaceae | *PIGU* | GCST90302717 | Weighted median | 1.95 × 10^-4^ | 1.17 (1.08, 1.28) |
| family Desulfovibrionaceae | *PIGU* | GCST90302717 | Inverse variance weighted | 1.84 × 10^-4^ | 1.14 (1.06, 1.22) |
| family Desulfovibrionaceae | *PIGU* | GCST90302717 | Simple mode | 0.09 | 1.17 (1.02, 1.33) |
| family Desulfovibrionaceae | *PIGU* | GCST90302717 | Weighted mode | 0.03 | 1.18 (1.07, 1.31) |
| family Desulfovibrionaceae | *PROCR* | GCST90302717 | MR Egger | 0.83 | 0.98 (0.85, 1.14) |
| family Desulfovibrionaceae | *PROCR* | GCST90302717 | Weighted median | 0.01 | 0.92 (0.86, 0.98) |
| family Desulfovibrionaceae | *PROCR* | GCST90302717 | Inverse variance weighted | 9.70 × 10^-5^ | 0.90 (0.85, 0.95) |
| family Desulfovibrionaceae | *PROCR* | GCST90302717 | Simple mode | 0.13 | 0.91 (0.83, 1.01) |
| family Desulfovibrionaceae | *PROCR* | GCST90302717 | Weighted mode | 0.06 | 0.91 (0.85, 0.98) |
| family Desulfovibrionaceae | *ZNF19* | GCST90302717 | MR Egger | 0.42 | 0.83 (0.57, 1.20) |
| family Desulfovibrionaceae | *ZNF19* | GCST90302717 | Weighted median | 0.14 | 1.11 (0.97, 1.27) |
| family Desulfovibrionaceae | *ZNF19* | GCST90302717 | Inverse variance weighted | 0.02 | 1.18 (1.02, 1.36) |
| family Desulfovibrionaceae | *ZNF19* | GCST90302717 | Simple mode | 0.46 | 1.10 (0.88, 1.38) |
| family Desulfovibrionaceae | *ZNF19* | GCST90302717 | Weighted mode | 0.47 | 1.08 (0.90, 1.28) |
| family Desulfovibrionaceae | *ZNF23* | GCST90302717 | MR Egger | 0.20 | 1.03 (0.99, 1.08) |
| family Desulfovibrionaceae | *ZNF23* | GCST90302717 | Weighted median | 0.48 | 1.01 (0.98, 1.04) |
| family Desulfovibrionaceae | *ZNF23* | GCST90302717 | Inverse variance weighted | 0.04 | 1.02 (1.00, 1.04) |
| family Desulfovibrionaceae | *ZNF23* | GCST90302717 | Simple mode | 0.72 | 1.01 (0.96, 1.06) |
| family Desulfovibrionaceae | *ZNF23* | GCST90302717 | Weighted mode | 0.48 | 1.01 (0.98, 1.04) |
