# Supplementary material for: Gut Microbiota and White Matter Integrity: A Two-Sample Mendelian Randomization Analysis
Source: eNeuro. 2025 Aug 29;12(9):ENEURO.0586-24.2025. doi: 10.1523/ENEURO.0586-24.2025 (PMC12418065; doi:10.1523/ENEURO.0586-24.2025)
Supplement: Figure 6-3 — Tests for heterogeneity and pleiotropy in the causal effect of bacterial taxa-mapped genes and their association with white matter connectivity. Download Figure 6-3, DOC file. [file eneuro-12-ENEURO.0586-24.2025-s012.doc]

Figure 6-3

Tests for heterogeneity and pleiotropy in the causal effect of bacterial taxa-mapped genes and their association with white matter connectivity

| Mendelian randomization | | | Sensitivity analysis | |
| --- | --- | --- | --- | --- |
| Gut microbiota | Exposure | Outcome | *p* (Heterogeneity test) | *p* (Pleiotropy test) |
| order Rhodospirillales | *DOCK10* | GCST90302666 | 0.06 | 0.46 |
| family Rhodospirillaceae | *CNR1* | GCST90302666 | 0.85 | 0.66 |
| family Rhodospirillaceae | *DOCK10* | GCST90302666 | 0.06 | 0.46 |
| family Rhodospirillaceae | *RRAGD* | GCST90302666 | 0.29 | 0.26 |
| family Rhodospirillaceae | *ZZZ3* | GCST90302666 | 0.50 | 0.42 |
| genus Escherichia Shigella | *ADAMTS13* | GCST90302698 | 0.59 | 0.19 |
| genus Escherichia Shigella | *ANKRD34B* | GCST90302698 | 0.75 | 0.12 |
| genus Escherichia Shigella | *CACFD1* | GCST90302698 | 0.13 | 0.95 |
| genus Escherichia Shigella | *DHFR* | GCST90302698 | 0.81 | 0.84 |
| genus Escherichia Shigella | *FAM151B* | GCST90302698 | 0.87 | 0.96 |
| genus Escherichia Shigella | *MED22* | GCST90302698 | 0.81 | 0.89 |
| genus Escherichia Shigella | *MSH3* | GCST90302698 | 0.18 | 0.33 |
| genus Escherichia Shigella | *NAA60* | GCST90302698 | 0.41 | 1.00 |
| genus Escherichia Shigella | *SURF6* | GCST90302698 | 0.41 | 0.19 |
| genus Escherichia Shigella | *TRNT1* | GCST90302698 | 0.69 | 0.43 |
| genus Howardella id.2000 | *COPS3* | GCST90302727 | 0.32 | 0.81 |
| genus Howardella id.2000 | *PLD6* | GCST90302727 | 0.58 | 0.11 |
| genus Ruminococcus gnavus group | *TFCP2L1* | GCST90302735 | 0.55 | 0.53 |
| genus Ruminococcus gnavus group | *TRIM33* | GCST90302735 | 0.61 | 0.52 |
| genus Senegalimassilia | *FBN2* | GCST90302795 | 0.38 | 0.65 |
| genus Senegalimassilia | *GALT* | GCST90302795 | 0.04 | 0.15 |
| genus Senegalimassilia | *ST6GALNAC1* | GCST90302795 | 0.31 | 0.48 |
| genus Tyzzerella3 | *ABCA7* | GCST90302660 | 0.45 | 0.88 |
| genus Tyzzerella3 | *CNN2* | GCST90302660 | 0.78 | 0.20 |
| genus Tyzzerella3 | *FARS2* | GCST90302660 | 0.91 | 0.90 |
| genus Tyzzerella3 | *POLR2E* | GCST90302660 | 0.78 | 0.45 |
| genus Tyzzerella3 | *VRK2* | GCST90302660 | 0.47 | 0.80 |
| genus Veillonella | *ACCS* | GCST90302696 | 0.43 | 0.35 |
| genus Veillonella | *KIAA0930* | GCST90302696 | 0.96 | 0.42 |
| genus Veillonella | *NAP1L4* | GCST90302696 | 0.49 | 0.25 |
| genus Veillonella | *SLC22A18* | GCST90302696 | 0.43 | 0.65 |
| genus Veillonella | *SLC22A18AS* | GCST90302696 | 0.72 | 0.41 |
| genus Veillonella | *TOR3A* | GCST90302696 | 0.70 | 0.97 |
| order Desulfovibrionales | *ACSS2* | GCST90302676 | 1.00 | 1.00 |
| order Desulfovibrionales | *CPNE1* | GCST90302676 | 0.22 | 0.96 |
| order Desulfovibrionales | *EDEM2* | GCST90302676 | 0.18 | 0.16 |
| order Desulfovibrionales | *EIF2S2* | GCST90302676 | 0.14 | 0.50 |
| order Desulfovibrionales | *EIF6* | GCST90302676 | 0.20 | 0.32 |
| order Desulfovibrionales | *ITCH* | GCST90302676 | 0.16 | 0.08 |
| order Desulfovibrionales | *MAP1LC3A* | GCST90302676 | 0.01 | 2.90× 10^-3^ |
| order Desulfovibrionales | *MMP24* | GCST90302676 | 0.17 | 0.15 |
| order Desulfovibrionales | *MYH7B* | GCST90302676 | 0.63 | 0.30 |
| order Desulfovibrionales | *PIGU* | GCST90302676 | 0.83 | 0.87 |
| order Desulfovibrionales | *PROCR* | GCST90302676 | 0.35 | 0.14 |
| order Desulfovibrionales | *MAP1LC3A* | GCST90302677 | 0.04 | 2.90× 10^-4^ |
| order Desulfovibrionales | *MMP24* | GCST90302677 | 0.19 | 0.40 |
| order Desulfovibrionales | *MYH7B* | GCST90302677 | 0.87 | 0.29 |
| order Desulfovibrionales | *PROCR* | GCST90302677 | 0.84 | 0.30 |
| order Desulfovibrionales | *ACSS2* | GCST90302677 | 0.69 | 0.86 |
| order Desulfovibrionales | *CPNE1* | GCST90302677 | 0.19 | 0.77 |
| order Desulfovibrionales | *EDEM2* | GCST90302677 | 0.44 | 0.06 |
| order Desulfovibrionales | *EIF2S2* | GCST90302677 | 0.41 | 0.54 |
| order Desulfovibrionales | *EIF6* | GCST90302677 | 0.19 | 0.84 |
| order Desulfovibrionales | *GSS* | GCST90302677 | 0.15 | 0.18 |
| order Desulfovibrionales | *HP* | GCST90302677 | 0.53 | 0.84 |
| order Desulfovibrionales | *IMPAD1* | GCST90302677 | 0.06 | 0.99 |
| order Desulfovibrionales | *ITCH* | GCST90302677 | 0.28 | 0.09 |
| order Desulfovibrionales | *ACSS2* | GCST90302717 | 0.50 | 0.65 |
| order Desulfovibrionales | *CPNE1* | GCST90302717 | 0.64 | 0.49 |
| order Desulfovibrionales | *ECHDC3* | GCST90302717 | 0.91 | 0.11 |
| order Desulfovibrionales | *EDEM2* | GCST90302717 | 0.01 | 0.69 |
| order Desulfovibrionales | *EIF6* | GCST90302717 | 0.01 | 0.56 |
| order Desulfovibrionales | *HP* | GCST90302717 | 0.82 | 0.55 |
| order Desulfovibrionales | *ITCH* | GCST90302717 | 0.08 | 0.29 |
| order Desulfovibrionales | *MMP24* | GCST90302717 | 0.09 | 0.10 |
| order Desulfovibrionales | *MYH7B* | GCST90302717 | 0.25 | 0.54 |
| order Desulfovibrionales | *PIGU* | GCST90302717 | 0.51 | 0.94 |
| order Desulfovibrionales | *PROCR* | GCST90302717 | 0.76 | 0.27 |
| order Desulfovibrionales | *ZNF19* | GCST90302717 | 0.11 | 0.19 |
| order Desulfovibrionales | *ZNF23* | GCST90302717 | 0.28 | 0.73 |
| family Desulfovibrionaceae | *ACSS2* | GCST90302717 | 0.50 | 0.65 |
| family Desulfovibrionaceae | *CPNE1* | GCST90302717 | 0.64 | 0.49 |
| family Desulfovibrionaceae | *ECHDC3* | GCST90302717 | 0.91 | 0.11 |
| family Desulfovibrionaceae | *EDEM2* | GCST90302717 | 0.01 | 0.69 |
| family Desulfovibrionaceae | *EIF6* | GCST90302717 | 0.01 | 0.56 |
| family Desulfovibrionaceae | *HP* | GCST90302717 | 0.82 | 0.55 |
| family Desulfovibrionaceae | *ITCH* | GCST90302717 | 0.08 | 0.29 |
| family Desulfovibrionaceae | *MMP24* | GCST90302717 | 0.09 | 0.10 |
| family Desulfovibrionaceae | *MYH7B* | GCST90302717 | 0.25 | 0.54 |
| family Desulfovibrionaceae | *PIGU* | GCST90302717 | 0.51 | 0.94 |
| family Desulfovibrionaceae | *PROCR* | GCST90302717 | 0.76 | 0.27 |
| family Desulfovibrionaceae | *ZNF19* | GCST90302717 | 0.11 | 0.19 |
| family Desulfovibrionaceae | *ZNF23* | GCST90302717 | 0.28 | 0.73 |
