# Supplementary material for: Gut Microbiota and White Matter Integrity: A Two-Sample Mendelian Randomization Analysis
Source: eNeuro. 2025 Aug 29;12(9):ENEURO.0586-24.2025. doi: 10.1523/ENEURO.0586-24.2025 (PMC12418065; doi:10.1523/ENEURO.0586-24.2025)
Supplement: Figure 6-4 — SMR analysis of bacterial taxa-mapped genes and their association with white matter connectivity. Download Figure 6-4, DOC file. [file eneuro-12-ENEURO.0586-24.2025-s013.doc]

Figure 6-4

SMR analysis of bacterial taxa-mapped genes and their association with white matter connectivity

| Exposure | Outcome | topSNP | *p*_SMR | *p*_HEIDI |
| --- | --- | --- | --- | --- |
| *CPNE1* | GCST90302677 | rs17093027 | 3.60 × 10^-3^ | 0.37 |
| *EDEM2* | GCST90302677 | rs7353271 | 0.05 | 0.51 |
| *EIF2S2* | GCST90302677 | rs6142101 | 0.04 | 0.09 |
| *ITCH* | GCST90302677 | rs1205344 | 0.03 | 0.98 |
| *MAP1LC3A* | GCST90302677 | rs6059919 | 0.31 | 0.51 |
| *MYH7B* | GCST90302677 | rs6120825 | 0.03 | 0.58 |
| *PROCR* | GCST90302677 | rs2273684 | 0.04 | 0.49 |
| *POLR2E* | GCST90302660 | rs11084882 | 0.48 | 0.10 |
| *ACSS2* | GCST90302717 | rs6060257 | 0.18 | 0.19 |
| *CPNE1* | GCST90302717 | rs17093027 | 0.03 | 1.00 |
| *MYH7B* | GCST90302717 | rs6120825 | 0.04 | 0.52 |
| *PIGU* | GCST90302717 | rs6088552 | 2.67× 10^-3^ | 0.95 |
| *PROCR* | GCST90302717 | rs2273684 | 0.04 | 0.80 |
| *FAM151B* | GCST90302698 | rs1677650 | 0.61 | 0.27 |
| *MED22* | GCST90302698 | rs120858 | 1.84× 10^-3^ | 0.84 |
| *SURF6* | GCST90302698 | rs12335 | 1.24× 10^-3^ | 0.79 |
| *ACSS2* | GCST90302676 | rs6060257 | 4.75× 10^-3^ | 0.20 |
| *CPNE1* | GCST90302676 | rs17093027 | 4.75×10^-3^ | 0.19 |
| *EDEM2* | GCST90302676 | rs7353271 | 0.02 | 0.57 |
| *ITCH* | GCST90302676 | rs1205344 | 0.02 | 0.91 |
| *MYH7B* | GCST90302676 | rs6120825 | 0.01 | 0.86 |
| *PROCR* | GCST90302676 | rs2273684 | 0.01 | 0.89 |
| *DOCK10* | GCST90302666 | rs6712372 | 1.25× 10^-3^ | 0.08 |
| *RRAGD* | GCST90302666 | rs9451216 | 0.06 | 0.83 |
| *ZZZ3* | GCST90302666 | rs9787306 | 0.32 | 0.46 |
| *ACCS* | GCST90302696 | rs2074038 | 0.79 | 0.12 |
| *COPS3* | GCST90302727 | rs4985761 | 0.05 | 0.82 |
| *GALT* | GCST90302795 | rs2070074 | 0.26 | 0.19 |
